# The Effect of Cluster Size on the Intra-Cluster Ionic Polymerization Process

**DOI:** 10.3390/molecules26164782

**Published:** 2021-08-07

**Authors:** Estefania Rossich Molina, Tamar Stein

**Affiliations:** Fritz Haber Research Center for Molecular Dynamics, The Hebrew University of Jerusalem, Jerusalem 9190401, Israel; erossich.molina@gmail.com

**Keywords:** acetylene, polycyclic aromatic hydrocarbons, interstellar medium, molecular growth, benzene, ab-initio molecular dynamics, van der Waals clusters, ion–molecule reactions’ astrochemistry

## Abstract

Polyaromatic hydrocarbons (PAHs) are widespread in the interstellar medium (ISM). The abundance and relevance of PAHs call for a clear understanding of their formation mechanisms, which, to date, have not been completely deciphered. Of particular interest is the formation of benzene, the basic building block of PAHs. It has been shown that the ionization of neutral clusters can lead to an intra-cluster ionic polymerization process that results in molecular growth. *Ab-initio* molecular dynamics (AIMD) studies in clusters consisting of 3–6 units of acetylene modeling ionization events under ISM conditions have shown maximum aggregation of three acetylene molecules forming bonded C_6_H_6_^+^ species; the larger the number of acetylene molecules, the higher the production of C_6_H_6_^+^. These results lead to the question of whether clusters larger than those studied thus far promote aggregation beyond three acetylene units and whether larger clusters can result in higher C_6_H_6_^+^ production. In this study, we report results from AIMD simulations modeling the ionization of 10 and 20 acetylene clusters. The simulations show aggregation of up to four acetylene units producing bonded C_8_H_8_^+^. Interestingly, C_8_H_8_^+^ bicyclic species were identified, setting a precedent for their astrochemical identification. Comparable reactivity rates were shown with 10 and 20 acetylene clusters.

## 1. Introduction

Polyaromatic hydrocarbons (PAHs) are widespread in the interstellar medium (ISM) [1,2,3]. They are believed to be ubiquitous and abundant in space, with interstellar IR spectra providing evidence of their presence in different areas by identifying their characteristic features in the spectra [4,5,6,7]. PAHs serve as a bridge between small organic molecules and large carbonaceous materials [8] and thus play an important role in the chemical evolution of the ISM.

Routes for the formation of PAHs and other organic molecules known to be present in the interstellar medium are not thoroughly understood. Various mechanisms for PAH formation and growth have been suggested on the basis of, for example, radical reactions [9,10,11,12,13,14,15]. One such example is the well-known hydrogen abstraction C_2_H_2_ addition (HACA) mechanism, which is based on two steps: the first is the formation of the aryl radical, and the second is the addition of acetylene (C_2_H_2_). The repetition of these two steps results in PAH formation. Other studies suggest the formation of naphthalene via the reaction of the phenyl radical and vinylacetylene or, alternatively, via the formation of naphthalene ions through ion–molecule reactions between benzene cations (C_6_H_6_)^+^ and ethynyl radicals [16]. The formation of benzene, the basic building block of PAHs, has drawn a great deal of attention. Different pathways for its formation have been proposed, depending on the physical and chemical conditions [17,18,19,20]. Jones et al. suggested a barrierless route for benzene formation via the reaction of the ethynyl radical and 1,3-butadiene, which is relevant to areas such as the Taurus Molecular Cloud (TMC-1) [21]. In several astrochemical models, routes for the formation of benzene are based on ion–molecule reactions [15,22,23,24,25]. Recently, Zhao et al. showed that molecular growth processes can occur via the reaction between two stabilized propargyl radicals at high temperature and diluted environments. Such a reaction can lead to the formation of benzene molecules, among other species [26]. 

Numerous experiments have demonstrated that molecular growth can occur via the intra-cluster ionic polymerization process [27,28,29,30,31,32,33,34,35,36,37,38,39,40]. Acetylene clusters have been the starting point of multiple experiments and, upon ionization of these clusters, covalently bonded structures, such as benzene and cyclobutadiene cations, have been observed. In addition, molecular growth has been observed experimentally for a cluster of ethynylbenzene, in which ionization leads to the formation of larger covalently bonded structures [32]. 

To understand the intra-cluster ionization process at the molecular level, several studies performed ab-initio molecular dynamics (AIMD) simulations modeling different neutral cluster compositions. In agreement with experimental work, AIMD simulations have demonstrated that the molecular growth process occurs and that covalently bonded structures are formed [38,41,42,43]. It has been shown that, upon ionization, part of the cluster forms covalently bonded core structures, while the remaining molecules serve as spectators of the process. The role of spectator molecules is important for the growth process, as these molecules change the potential energy surfaces and thus play a catalytic role in the formation of various core structures. Moreover, spectator molecules enable stabilization routes, as they can dissipate the excess energy via evaporation [38,42]. For example, upon ionization of acetylene clusters containing up to six acetylene units, the formation of bonded C_4_H_4_^+^ and C_6_H_6_^+^ including benzene cations was also observed [38]. Ionization of small clusters (up to five units) containing acetylene and hydrogen cyanide also led to molecular growth, in which three and four units bonded to form structures on the potential energy surfaces of C_6_H_6_^+^, C_5_H_5_N^+^, C_7_H_7_N^+^, and C_8_H_8_^+^. Likewise, when the clusters are built from acetylene and cyanoacetylene units, building blocks that can be found in TMC-1, the ionization process leads to bonding between three and four units [41]. Many of the structures that are formed are aromatic structures and contain a nitrogen atom within the ring or as a side chain; thus, the structures are important from an astrobiological point of view. Among the formed structures we observed was the benzonitrile cation, which was recently identified in its neutral form in TMC-1 [44]. 

AIMD results from previous studies demonstrate the importance of the composition and size of the cluster in promoting aggregation to yield chemically bonded structures. In reference to pure acetylene clusters, in particular, it has been demonstrated that the larger clusters (five and six acetylene units) enable higher rates of bonded C_6_H_6_^+^ formation in comparison with the smaller ones (three and four acetylene units) [38]. Here, we focus on the study of pure acetylene clusters to understand the influence of a large number (10 and 20, significantly larger than the six clusters studied to date) of acetylene molecules in the aggregation process that produces (C_2_H_2_)_n_^+^ species following ionization of the van der Waals clusters. Specifically, our aim was to investigate the effect of cluster size on the percentage of C_6_H_6_^+^ produced in the larger clusters vs. the smaller ones. Additionally, we examined the extent of molecular growth in the larger clusters.

## 2. Results

**Figure 1:**
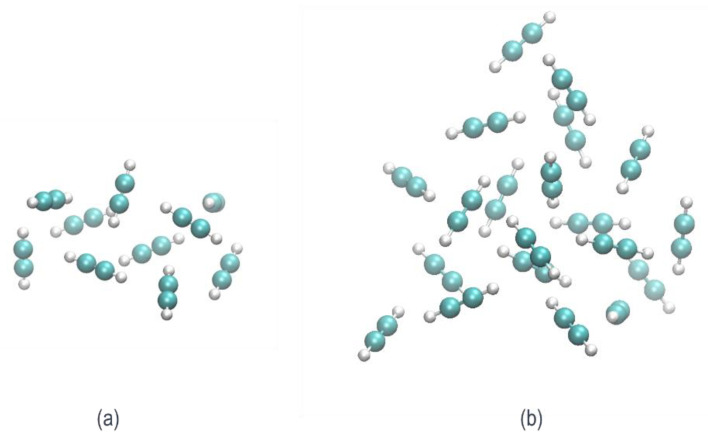
Pure neutral acetylene clusters consisting of (**a**) 10 units of acetylene (decamer) and (**b**) 20 units of acetylene (eicosamer).

In order to address the aforementioned objectives, we studied large van der Waals clusters containing 10 and 20 units of acetylene. We built 40 structures: 20 decamers and 20 eicosamers (10 and 20 acetylene units, respectively), considering different relative orientations of the acetylene molecules that maximize the CH-π interaction between hydrogen and the π clouds of neighboring acetylenes, and we then optimized them to obtain minima on the neutral potential energy surface (PES) [40]. Figure 1 shows examples of a decamer structure (Figure 1a) and an eicosamer structure (Figure 1b). The complete set of coordinates corresponding to the optimized AIMD starting structures are available in the Supplementary Materials. In order to model the ionization process, the optimized neutral structures were utilized as starting structures in the molecular dynamic simulations on the cationic PES. 

**Figure 2:**
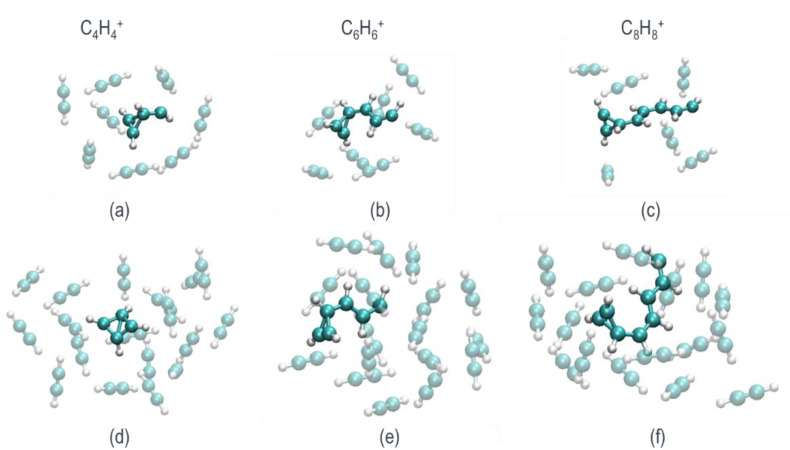
(**a**–**c**) are decamer clusters optimized on the cationic PES with C_4_H_4_^+^, C_6_H_6_^+^, and C_8_H_8_^+^ core-bonded structures, respectively; (**d**–**f**) correspond to the eicosamer clusters that also show C_4_H_4_^+^, C_6_H_6_^+^, and C_8_H_8_^+^ core-bonded structures.

The neutral cluster geometries are nonoptimal after vertical ionization, and they relax on the cationic PES, yielding bonded structures. Figure 2 presents the structures after optimization on the cationic PES. In both cases (decamer and eicosamer), the clusters relax to core-bonded structures in the C_4_H_4_^+^, C_6_H_6_^+^, and C_8_H_8_^+^ PES (Figure 2a–c for the decamer and Figure 2d–f for the eicosamer) without any barriers. The core structures are solvated by additional nonbonded acetylene molecules. We note that the largest core structure that is formed upon optimization is a bonded C_8_H_8_^+^. The coordinates of all 40 optimized cationic isomers are reported in the SI.

### 2.1. AIMD Simulations

Following ionization and the formation of the core structures, the system contains a large amount of excess energy (~5 eV) that can be utilized to cross barriers on the PES. In order to study the evolution of the systems over time and the potential formation of different products upon ionization, we used AIMD. The results of the AIMD simulations shed light on the structures that can be formed at the end of this process and the relative probability of their formation. The structures of the most relevant mono- and bicyclic molecules obtained are presented, and a comparison is made between the decamers and eicosamers as well as smaller acetylene van der Waals clusters previously studied by means of AIMD under similar conditions [38]. We analyze the extent of the molecular growth as a function of the number of acetylene molecules in a cluster and conclude the relevance of the present results in understanding PAH formation routes and astrochemical identification of cyclic molecules.

### 2.2. AIMD of the Acetylene Decamer Clusters

**Figure 3:**
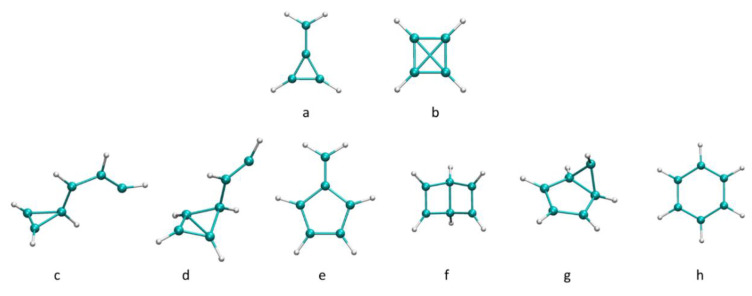
Optimized bonded C_4_H_4_^+^ and C_6_H_6_^+^ core structures. (**a**,**b**): isomers on the C_4_H_4_^+^ PES. (**c**–**h**): isomers on the C_6_H_6_^+^ PES.

For each of the 20 starting structures, 30 trajectories were run, totaling 600 trajectories. All trajectories were performed for 2.4 ps. For trajectories ending in structures where further potential rearrangements or growth were suspected, the simulation time was extended up to 4.8 ps. The resulting core structures are shown in Figure 3. From the 600 trajectories for this cluster size, we found that 56.5% of the trajectories led to a molecular product of the reaction of 2 units of acetylene to yield bonded C_4_H_4_^+^. The vast majority of the C_4_H_4_^+^ species produced corresponded to cyclobutadiene (Figure 3a), while a minor percentage was methylenecyclopropane (Figure 3b). In other trajectories, which represent 40.8% of the trajectories, three units of acetylene react to yield bonded C_6_H_6_^+^. Multiple C_6_H_6_^+^ products were identified. Among them were benzene (Figure 3h) and its conformational isomers fulvene (Figure 3e), dewar-benzene (Figure 3f), and benzvalene (Figure 3g), all of which are known to easily interconvert into benzene. In a previous study, these products were also identified in the products from the ionization of clusters up to hexamer, although in smaller percentages [38]. Interestingly, here we observed a higher percentage of the trajectories leading to bonded C_6_H_6_^+^, which reinforces the important role played by the cluster environment. In the remaining ~2.7% of the trajectories, we observed the aggregation of four acetylene molecules to yield bonded C_8_H_8_^+^. While other cluster compositions (namely, mixed acetylene with HCN and cyanoacetylene) enable bonding between four units [41,43], C_8_H_8_^+^ species were not obtained from the acetylene hexamers previously studied, clearly demonstrating that more than six acetylene units are required to observe aggregation beyond C_6_ structures from a pure acetylene cluster [38,42]. Again, this emphasizes the role of the cluster environment not only in the stabilization of the products but also in their formation.

**Figure 4:**
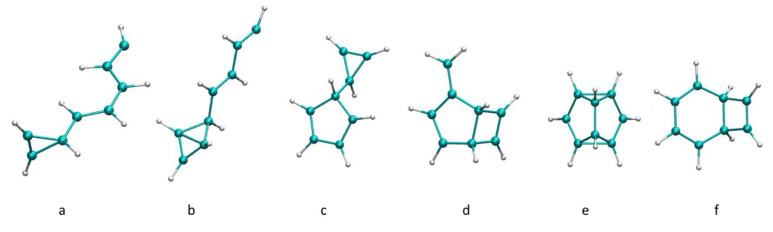
Optimized bonded C_8_H_8_^+^ core structures. (**a**–**f**): isomers on the C_8_H_8_^+^ PES.

**Figure 5:**
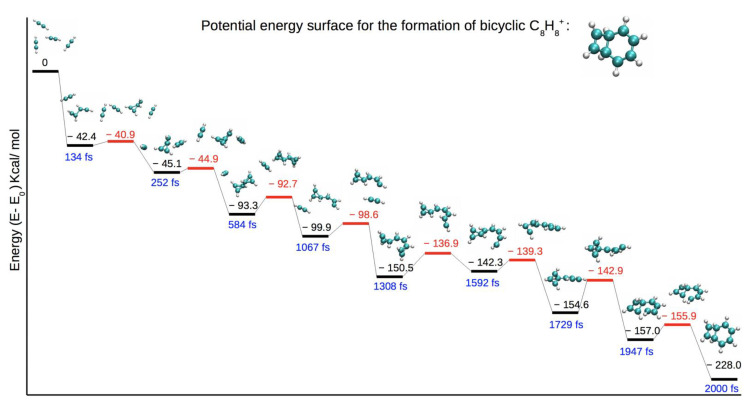
Potential energy surface for the production of bicyclic C_8_H_8_^+^ structure containing a four- and six-member ring. The minima energies are reported in black, and the transition state energies are in red. The corresponding time of each structure during the molecular dynamics trajectory is noted in blue.

The largest bonded structures obtained corresponded to the aggregation of four acetylene units to yield C_8_H_8_^+^ isomers, as shown in Figure 4. Among these products, we detected structures that contained a three-member (Figure 4a) or four-member (Figure 4b) ring, as observed in the C_4_H_4_^+^ and C_6_H_6_^+^ structures. Trajectories leading to C_8_H_8_^+^ were extended for an additional 2.4 ps. At these longer times, C_8_H_8_^+^ products further react, yielding bicyclic products with five- and three- (Figure 4c), five- and four- (Figure 4d), five- and five- (Figure 4e), and six-and four- (Figure 4f) member ring structures. In the literature, C_8_H_8_^+^ is attributed to a benzene cation complexed to an acetylene molecule, which we also observed in our simulations [33]. The PES for the formation of the structure in Figure 4f is presented in Figure 5, along with the corresponding time of occurrence of each structure during the simulation time. At the first stages of the simulation, two acetylene molecules were bonded together, and a three-member ring structure (Figure 5, 252 fs) was formed. This resembled the association product of acetylene and its cation observed by Bera et al. [45]. Afterward, an additional bond formed with a neighboring acetylene molecule, resulting in a bicyclic structure (Figure 5, 584 fs), which later reorganized into a three-member cyclic structure with a long chain (Figure 5, 1067 fs). A similar structure was also seen when all four acetylene molecules were bonded (Figure 5, 1592 fs). We observed a transition to a four-member ring (Figure 5, 1729 fs), which reorganized to produce the final product. 

A bicyclic C_8_H_8_^+^ molecule with five- and three-member rings (Figure 4c) was observed in two of the trajectories. One of them dissociated in a later stage into C_5_H_5_ and c-C_3_H_3_^+^, which was experimentally detected [46], and its direct observation in the ISM is only feasible via ro-vibrational transitions because of the absence of pure rotational transitions [46].

### 2.3. Comparison of the Growth Process in Different Cluster Sizes

**Figure 6:**
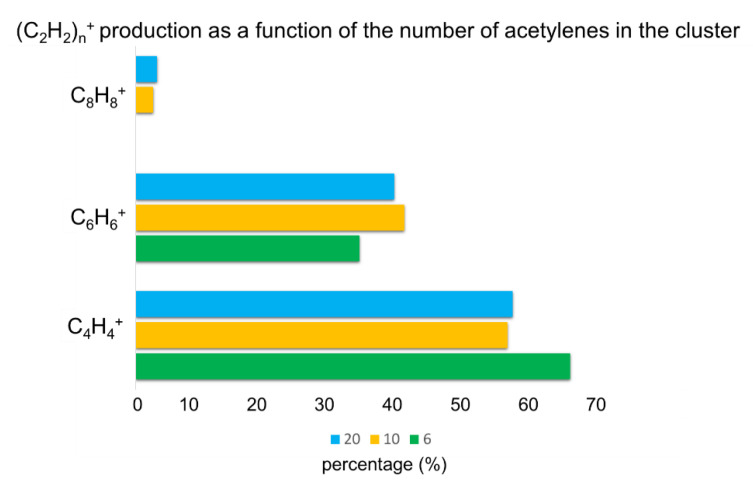
Histogram of the percentage of (C_2_H_2_)_n_^+^ *n* = 2–4 bonded structures obtained upon ionization of the clusters as a function of the number of acetylene molecules in them. Percentages for six acetylene molecules were taken from the literature [38]. The percentages for 10 and 20 acetylene molecules correspond to the results in the present work.

Starting from 20 eicosamer optimized structures, we ran a total of 600 trajectories at 2.4 ps. The results for this cluster size were similar to those obtained for the decamer clusters; no additional structures were found when the AIMD started from the eicosamer clusters. Figure 6 shows a comparison of the number of core structures obtained at the end of the AIMD simulations of different cluster sizes consisting of 6, 10, and 20 acetylene units. In the case of the hexamer clusters, it can be seen that the majority of trajectories formed C_4_H_4_^+^ core structures, while the rest of the trajectories formed C_6_H_6_^+^ core structures. No C_8_H_8_^+^ structure was observed. In the case of the decamer and eicosamer clusters, more C_6_H_6_^+^ was obtained with respect to the hexamer clusters. Additionally, these cluster sizes enabled the formation of C_8_H_8_^+^ structures that were not observed in the smaller clusters. In fact, a previous photoionization mass spectrometry study demonstrated that C_8_H_8_^+^ can be obtained from acetylene and is identified by a peak at *m/z* = 104 [38]. The percentages for the formation of different structures were similar for the decamers and eicosamers; no significant differences were observed between them.

**Figure 7:**
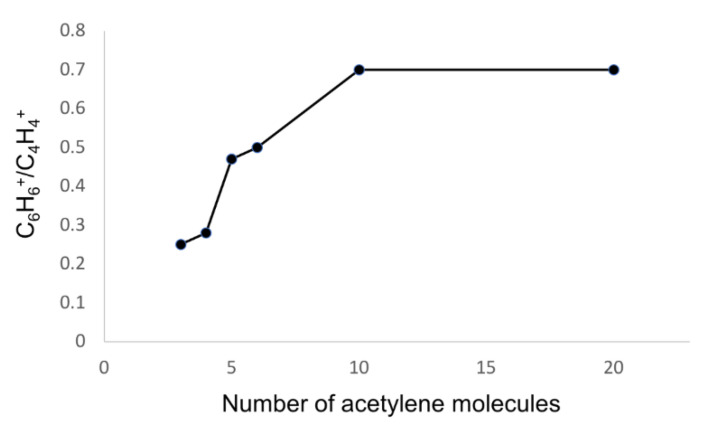
The C_6_H_6_^+^/C_4_H_4_^+^ ratio as a function of the number of acetylene units in the cluster.

The C_6_H_6_^+^/C_4_H_4_^+^ ratio is a way to express the capacity of molecular growth in acetylene clusters. A previous study found that the C_6_H_6_^+^/C_4_H_4_^+^ ratio was ~0.3 for acetylene dimers and trimers, and the C_6_H_6_^+^/C_4_H_4_^+^ ratio was ~0.5 for tetramers and pentamers [38]. Here, we found that the C_6_H_6_^+^/C_4_H_4_^+^ ratio was 0.7 for decamers and eicosamers. Figure 7 summarizes the trend of the C_6_H_6_^+^/C_4_H_4_^+^ ratio for pure acetylene clusters as a function of the number of acetylenes and is an adaptation of a figure from a previous work [38] in order to add the results from the present study. Again, the results demonstrate that a growing number of acetylene spectator molecules in a cluster promotes the molecular growth process by the process described above. As can be seen from the figure, decamers and eicosamers present comparable reactivity; eicosamers do not lead to further aggregation (e.g., C_10_H_10_^+^ and beyond) despite having double the number of acetylenes. This suggests that the addition of acetylene molecules to the large clusters’ environment (beyond the decamers studied here) does not affect the environment substantially and does not prompt additional growth. We note, however, that it is also plausible that clusters containing species other than acetylene can be involved in catalyzing the formation of structures larger than C_8_H_8_^+^. 

## 3. Materials and Methods

Every calculation in this manuscript was performed using Q-Chem 5.1 software [47]. Optimizations of neutral and cationic structures were accomplished with the wB97X-V functional [48] and cc-pVTZ basis set [49]. Using the same level of theory, we performed vibrational frequency calculations to categorize the structures as minima or saddle points on the PES. 

Starting from the optimized minima structures of isomers of clusters consisting of 10 or 20 units of acetylene, we ran AIMD simulations on the cationic PES utilizing the ωB97 functional [50] and 6–31G* basis set [51]. The initial velocities for the dynamics were the thermal ones corresponding to the randomly sampled temperatures in the range of 30–80 K. Each of the 30 trajectories for each isomer was run for ~2.4 ps (time step = 1.21fs) unless otherwise stated. Twenty different starting structures were considered per system size (decamer and eicosamer), giving a total of 1200 trajectories.

## 4. Conclusions

We studied ionic polymerization processes on large clusters containing 10 and 20 acetylene molecules in conditions relevant to the ISM by means of AIMD. Our results reinforce the observations of previous studies on clusters consisting of 3–6 acetylene units, i.e., an increased number of spectator acetylenes promotes molecular growth. We observed a larger C_6_H_6_^+^/C_4_H_4_^+^ ratio for decamers and eicosamers than what was previously found for acetylene clusters up to hexamers. Additionally, we observed C_8_H_8_^+^ structures that were not obtained from smaller clusters (consisting of 3–6 units of acetylene). Eicosamer clusters exhibit results similar to those of the decamer clusters, demonstrating that the additional 10 acetylene molecules do not significantly influence the environment to promote further growth. Moreover, we predict that the formation of mono- and bi-cyclic structures with formulas (C_2_H_2_)_n_^+^ n = 2–4 produced by ionic polymerization processes can serve as a guide for astronomers in search of new molecules in the ISM. 

## Data Availability

Data is contained within the article or Supplementary Material.

## Supplementary Materials

The following are available online. Section 1: Optimized neutral structures; Section 2: Optimized cationic structures.
