# Supplementary material for: The Effect of Cluster Size on the Intra-Cluster Ionic Polymerization Process
Source: Molecules. 2021 Aug 7;26(16):4782. doi: 10.3390/molecules26164782 (PMC8399435; doi:10.3390/molecules26164782)
Supplement: Supplementary file 1 [file molecules-26-04782-s001.zip › molecules-1325525-supplementary.pdf]

The effect of cluster size on the intra-cluster ionic polymerization process

Estefania Rossich Molina[1] and Tamar Stein[1]

[1]Fritz Haber Research Center for Molecular Dynamics, The Hebrew University of Jerusalem, Jerusalem, Israel, 9190401

*Corresponding author: tamar.stein@mail.huji.ac.il, Tel: +972-2-658-6108

Section 1 reports the cartesian coordinates of all optimized neutral structures for the decamers and eicosamers, used as starting structures in molecular dynamics simulations. “DEC” is used to report the decamers, while “EICO” is used to report the eicosamers.
In Section 2, the corresponding optimized cationic structures for each isomer are reported.
Indexes 1 to 20 were used to identify the correspondence between neutral and ionic structures at each cluster size.

Section 1: Optimized neutral structures

DEC 1

0 1

C	4.835087	3.147014	-0.256465
H	5.303762	4.092289	-0.408081
C	4.846693	-1.875739	-0.612386
H	4.035231	-2.485598	-0.946677
C	5.746639	-1.184900	-0.229840
H	6.546564	-0.562915	0.101253
C	4.313060	2.083314	-0.083370
H	3.841693	1.137080	0.074559
C	0.948010	3.319471	-0.636404
H	1.994525	3.311021	-0.418532
C	-0.224103	3.328092	-0.884626
H	-1.268863	3.332220	-1.109551
C	-0.736431	0.023267	-1.863018
H	-0.120168	0.768406	-2.315171
C	-1.427369	-0.797845	-1.329640
H	-2.037093	-1.524555	-0.838001
C	1.374503	-3.161393	-1.066281
H	0.902563	-2.228014	-1.289926
C	1.887753	-4.211454	-0.804214
H	2.335098	-5.151418	-0.574043
C	-2.236007	-3.021814	1.783366
H	-3.278070	-3.214912	1.901358
C	1.308713	0.060318	1.156867
H	0.492776	0.715846	0.938199
C	2.219161	-0.679316	1.402928
H	3.040584	-1.328734	1.607775
C	-1.068205	-2.799638	1.636418
H	-0.030251	-2.590388	1.494823
C	-4.602659	-0.823780	0.372177
H	-4.044960	-0.203094	1.041723
C	-5.214039	-1.517301	-0.389120
H	-5.765295	-2.127985	-1.067075
C	-3.707449	2.305613	-1.330393
H	-3.389738	1.291437	-1.446532
C	-4.053500	3.444896	-1.200364
H	-4.374368	4.456048	-1.093507
C	-2.259511	1.672577	1.914295
H	-2.543351	2.572144	1.416011
C	-1.944679	0.656562	2.464944
H	-1.658448	-0.257606	2.935852

DEC 2

0 1

C	-0.579571	-1.417488	1.283163

C	-0.959046	-1.884740	2.319095
H	-0.238436	-1.001427	0.358204
H	-1.298816	-2.305601	3.237865
C	-1.495412	3.125846	-2.941352
C	-0.558013	3.708979	-2.477116
H	-2.327427	2.596532	-3.347557
H	0.280115	4.214999	-2.048792
C	-3.436905	-3.549808	0.050490
C	-3.825352	-4.654909	-0.197889
H	-3.079161	-2.566636	0.267153
H	-4.181805	-5.635501	-0.415275
C	-1.470958	3.079495	0.944985
C	-1.117322	2.147683	1.609314
H	-1.773642	3.906452	0.343702
H	-0.784518	1.319073	2.194961
C	1.109593	0.661814	-1.440739
C	2.001416	0.177357	-0.802956
H	0.312913	1.107025	-1.997401
H	2.783717	-0.264972	-0.226039
C	0.590321	-2.969675	-2.059853
C	-0.244785	-3.633204	-1.514802
H	1.329088	-2.363927	-2.534407
H	-1.005897	-4.203487	-1.028253
C	2.215619	1.665337	2.935008
C	2.076942	0.481273	2.820647
H	2.330811	2.721967	3.024637
H	1.949386	-0.573131	2.708056
C	1.960680	3.893740	0.117328
C	2.213379	5.063974	0.079368
H	1.732475	2.849182	0.145654
H	2.444449	6.104037	0.051858
C	2.733436	-2.871748	0.957769
C	3.727664	-2.512234	1.520607
H	1.848829	-3.186414	0.446389
H	4.612166	-2.196929	2.025609
C	-2.474966	-0.701995	-1.992736
C	-2.483449	0.202047	-1.206627
H	-2.459191	-1.518171	-2.679132
H	-2.460970	1.002512	-0.497753

DEC 3

0 1

C	4.509700	0.740629	-0.287207
C	5.192664	1.442630	-0.976218
H	3.898770	0.115131	0.327297
H	5.805772	2.063399	-1.588552
C	0.925645	3.986242	-0.706973
C	1.979148	3.435361	-0.562218
H	-0.023353	4.456712	-0.833486
H	2.908138	2.924026	-0.428624
C	-0.117249	-2.291643	3.331652
C	0.172916	-3.394669	3.698242
H	-0.370745	-1.308861	2.994491
H	0.422866	-4.374065	4.037492
C	2.746610	-2.389817	-0.495864
C	2.366990	-2.489334	0.635453
H	3.089926	-2.286473	-1.500542
H	2.022389	-2.566827	1.643379
C	-2.557758	3.659047	-2.211223
C	-2.474499	2.872801	-1.311815
H	-2.639460	4.356242	-3.013286

H	-2.405472	2.162136	-0.516071
C	-4.661936	-0.776284	-2.065189
C	-3.547127	-0.399248	-2.289236
H	-5.658777	-1.098904	-1.869382
H	-2.550302	-0.062617	-2.479849
C	-2.199903	-3.358102	-0.797415
C	-1.493771	-3.499400	0.159639
H	-2.831008	-3.206778	-1.644365
H	-0.871348	-3.617617	1.018746
C	0.210193	0.601147	-1.979172
C	0.016306	-0.536257	-1.652940
H	0.386835	1.621838	-2.238351
H	-0.154723	-1.547092	-1.351402
C	-1.966290	0.883013	1.892786
C	-2.376280	-0.034293	1.239729
H	-1.587533	1.691985	2.474963
H	-2.731958	-0.857382	0.657712
C	1.895368	0.804887	2.721929
C	1.392894	0.738260	1.636403
H	2.347078	0.877103	3.684758
H	0.942722	0.673957	0.666420

DEC4

0 1

C	5.158334	0.587004	-0.729032
C	4.893038	1.438422	-1.528614
H	5.376300	-0.175512	-0.012091
H	4.651205	2.196090	-2.239093
C	2.047539	0.955619	1.161399
C	1.152658	0.513803	1.823318
H	2.847147	1.340463	0.566695
H	0.350268	0.121047	2.405906
C	-3.845548	0.673222	4.149946
C	-4.217383	0.170786	3.128905
H	-3.516407	1.113336	5.063148
H	-4.544040	-0.268058	2.210867
C	-2.185318	-0.818677	-2.499262
C	-1.452967	-0.037474	-3.035210
H	-2.841333	-1.505696	-2.013569
H	-0.794961	0.657020	-3.507754
C	4.164124	-2.051314	1.477391
C	5.266962	-2.442069	1.731736
H	3.184963	-1.692499	1.243245
H	6.243827	-2.796701	1.966897
C	-5.638305	-0.766349	-0.338886
H	-6.334237	-1.555571	-0.167586
C	-1.545511	2.180090	0.088686
H	-0.788639	2.084132	-0.660162
C	-2.375852	2.290411	0.945668
H	-3.104078	2.373376	1.721772
C	-4.862517	0.123253	-0.540547
H	-4.161161	0.909555	-0.720063
C	-1.710707	-1.419937	0.894655
H	-1.902922	-0.377033	0.752402
C	-1.479617	-2.584032	1.056767
H	-1.273964	-3.619679	1.203904
C	1.417284	-1.747090	-1.319997
H	0.414299	-1.707001	-0.952624
C	2.542684	-1.787367	-1.728762
H	3.547996	-1.819793	-2.082908
C	1.483611	1.791661	-2.266330
H	1.749018	0.782328	-2.024862

C	1.176908	2.919360	-2.531180
H	0.907957	3.924978	-2.762189

DEC 5

0 1

C	-3.916705	-1.040678	0.360137
C	-4.795146	-1.833321	0.171442
H	-3.131154	-0.333046	0.525023
H	-5.577843	-2.538860	0.009057
C	-0.485603	-3.170957	-2.067772
C	-1.577779	-2.722595	-1.865720
H	0.493630	-3.557542	-2.238879
H	-2.550046	-2.326403	-1.670049
C	-6.831795	2.778136	-0.012059
C	-6.373831	1.679039	0.113543
H	-7.246111	3.753686	-0.125901
H	-5.954224	0.701483	0.220112
C	-1.765670	2.087649	-0.361299
C	-2.895579	2.441790	-0.174179
H	-0.758520	1.769007	-0.532006
H	-3.909171	2.742908	-0.020769
C	-0.294890	-0.422591	1.846455
C	-0.559373	-1.499028	1.392489
H	-0.057348	0.544335	2.234380
H	-0.809270	-2.444086	0.965883
C	1.897602	1.096518	-1.323342
C	1.146783	0.169893	-1.438307
H	2.576004	1.913294	-1.220410
H	0.475407	-0.656856	-1.531777
C	3.080463	-2.650605	-0.957303
C	2.572014	-3.362881	-0.139663
H	3.540204	-1.995226	-1.665250
H	2.124607	-3.992649	0.595754
C	5.518343	0.260906	-2.501622
C	5.400070	0.125026	-1.318006
H	5.626805	0.387754	-3.554115
H	5.279130	0.003249	-0.263252
C	3.496915	-0.227737	1.782779
C	4.409175	0.202837	2.429418
H	2.686223	-0.611925	1.199872
H	5.221614	0.574111	3.011601
C	0.479943	3.337216	1.914839
C	1.498817	2.756035	2.155500
H	-0.432368	3.842037	1.690074
H	2.398676	2.220074	2.363322

DEC 6

0 1

C	3.160054	-3.947271	0.522252
C	2.771329	-5.079608	0.502263
H	3.512705	-2.938078	0.541302
H	2.431883	-6.090018	0.485962
C	3.051426	3.888497	-1.110632
C	3.491931	2.799168	-1.340241
H	2.661691	4.860585	-0.911139
H	3.884401	1.824442	-1.528327
C	4.657560	-0.313853	0.667925
C	3.472659	-0.257486	0.499294
H	5.711783	-0.338107	0.829017

H	2.415320	-0.189614	0.349794
C	0.326923	1.079938	-0.566423
C	-0.224028	0.067041	-0.239718
H	0.826607	1.980166	-0.851445
H	-0.716006	-0.836478	0.053191
C	-4.643133	2.918497	1.824507
C	-5.279271	2.400072	2.696266
H	-4.072302	3.365564	1.038527
H	-5.845342	1.945507	3.476645
C	-1.826817	-3.272069	0.688564
C	-0.657814	-3.526377	0.622864
H	-2.867721	-3.040843	0.735322
H	0.385356	-3.755585	0.570834
C	7.678955	2.326588	0.950035
C	6.534559	2.638074	0.789694
H	8.700682	2.059060	1.092416
H	5.514066	2.915713	0.642806
C	-3.665315	-0.727406	-1.024084
C	-4.060370	-0.025192	-0.137643
H	-3.316926	-1.369893	-1.801110
H	-4.408789	0.609512	0.649044
C	-4.320547	-4.584559	-1.509481
C	-5.212273	-3.791097	-1.422425
H	-3.522925	-5.288397	-1.578819
H	-5.999391	-3.076657	-1.341307
C	-2.507345	3.115588	-1.173529
C	-2.752464	4.285628	-1.245673
H	-2.288035	2.071672	-1.103850
H	-2.970703	5.326118	-1.321658

DEC 7

0 1

C	1.155365	-3.917894	1.847158
C	0.488662	-2.925470	1.916491
H	1.749730	-4.800930	1.780288
H	-0.094588	-2.030208	1.964725
C	3.918928	-3.451580	-0.935581
C	3.394197	-4.402230	-1.439738
H	4.372270	-2.596588	-0.485215
H	2.932370	-5.248948	-1.893922
C	3.688027	0.226640	0.705654
C	3.191971	-0.742988	1.204358
H	4.113354	1.096904	0.258707
H	2.730812	-1.605306	1.637307
C	1.508338	3.162321	-0.954072
C	1.352933	1.985412	-1.117884
H	1.625812	4.214956	-0.815879
H	1.207696	0.935333	-1.256415
C	-1.277465	0.554908	1.952417
C	-0.097476	0.736218	1.851434
H	-2.329723	0.394871	2.025168
H	0.954451	0.891663	1.748137
C	-0.446979	-1.174556	-1.377602
C	0.590346	-1.774348	-1.413979
H	-1.375523	-0.647389	-1.329274
H	1.511868	-2.315937	-1.434733
C	-3.361711	-4.680792	-0.072791
C	-2.299158	-4.141113	-0.188858
H	-4.300214	-5.174781	0.032246
H	-1.353251	-3.654379	-0.293139
C	-3.951087	0.326579	-0.498496
C	-3.919795	-0.865465	-0.382079

H	-3.963582	1.389862	-0.597921
H	-3.876402	-1.929270	-0.283466
C	-2.094499	3.450250	-0.123015
C	-3.061305	4.145119	-0.258630
H	-1.228536	2.833603	-0.000777
H	-3.923693	4.761674	-0.376492
C	1.114722	7.073543	-0.396760
C	0.120345	6.414372	-0.299584
H	1.996740	7.665311	-0.481549
H	-0.763953	5.820638	-0.216235

DEC 8

0 1

C	0.023693	3.466510	-1.484733
C	-0.172924	2.808976	-0.491464
H	0.201197	4.049527	-2.360998
H	-0.365238	2.223434	0.385764
C	4.488807	3.925441	0.826664
C	3.424537	3.659216	0.324779
H	5.426005	4.167761	1.275590
H	2.471950	3.437466	-0.109733
C	6.840754	0.519932	-1.754992
C	6.044166	1.244001	-1.210211
H	7.546986	-0.112687	-2.244399
H	5.345424	1.896770	-0.728304
C	4.735954	-2.106874	-0.013346
C	4.093650	-3.043546	0.395508
H	5.309264	-1.278109	-0.374363
H	3.501346	-3.860533	0.750077
C	-3.880479	0.060494	-1.633030
C	-3.037263	0.924272	-1.669934
H	-4.630248	-0.703176	-1.600512
H	-2.281431	1.681467	-1.697129
C	1.062775	-1.483806	-0.795372
C	-0.116755	-1.250117	-0.907861
H	2.106425	-1.700277	-0.688383
H	-1.153584	-1.013567	-1.022852
C	-6.518915	-2.679390	-1.877178
C	-6.528241	-2.438892	-0.695619
H	-6.516176	-2.901373	-2.921009
H	-6.539923	-2.232330	0.351515
C	0.846908	-4.678216	0.909073
C	1.265270	-5.639841	1.505389
H	0.490643	-3.818401	0.380340
H	1.617574	-6.497461	2.033179
C	-4.853212	2.267799	1.375347
C	-5.157909	2.822885	2.402197
H	-4.574238	1.774258	0.467282
H	-5.441421	3.318977	3.303223
C	-0.693538	0.313273	2.200535
C	-1.656985	1.001142	2.435308
H	0.142118	-0.307748	1.964374
H	-2.526348	1.595151	2.623089

DEC 9

0 1

C	-0.070735	3.650745	-1.780403
C	-0.224718	2.914596	-0.836220
H	0.069863	4.303739	-2.612781
H	-0.365716	2.260860	0.000584

C	4.346357	4.227992	0.610817
C	3.312213	3.900557	0.082831
H	5.257264	4.523276	1.081650
H	2.385039	3.628022	-0.376990
C	6.951875	0.497870	-1.099566
C	6.083600	1.273927	-0.784240
H	7.722648	-0.181745	-1.387072
H	5.323222	1.974128	-0.505052
C	4.539898	-2.016528	0.370339
C	3.811283	-2.933111	0.663846
H	5.185815	-1.203740	0.109891
H	3.144294	-3.732727	0.909393
C	-3.669906	0.033541	-1.354657
C	-3.123001	1.044048	-1.725295
H	-4.118317	-0.873548	-1.011313
H	-2.610203	1.929178	-2.034579
C	1.125182	-1.486795	-1.190180
C	-0.016963	-1.146595	-1.384885
H	2.135719	-1.784440	-1.000524
H	-1.023291	-0.827652	-1.556011
C	-3.305980	-3.416984	0.465640
C	-2.798875	-2.452966	0.984850
H	-3.746770	-4.282115	0.023106
H	-2.341463	-1.596934	1.436010
C	0.510570	-4.653227	0.631373
C	1.012512	-5.603262	1.180109
H	0.073311	-3.807020	0.144445
H	1.435389	-6.453752	1.666030
C	-4.512045	2.347986	1.609356
C	-4.671263	2.875519	2.682513
H	-4.360034	1.877407	0.660317
H	-4.829917	3.349820	3.625057
C	-0.362447	0.274337	1.855393
C	-1.300690	0.925124	2.248308
H	0.451092	-0.304773	1.475715
H	-2.140590	1.499689	2.578630

DEC 10

0 1

C	-2.271891	-1.172738	-2.080760
C	-1.214298	-0.706014	-1.764564
H	-3.219321	-1.578332	-2.364322
H	-0.274119	-0.291891	-1.470576
C	1.884670	0.979676	0.039766
C	1.098629	0.406925	0.737837
H	2.588991	1.492953	-0.573971
H	0.389903	-0.105769	1.352214
C	-1.730021	-1.145742	2.847517
C	-1.890934	-1.563199	1.736240
H	-1.591086	-0.787390	3.842051
H	-2.040459	-1.925921	0.741393
C	-5.403674	0.715317	-0.217727
C	-4.514299	0.612832	0.578976
H	-6.193120	0.809955	-0.932585
H	-3.716522	0.500682	1.279913
C	-4.308231	-4.107912	-0.357374
C	-4.709091	-2.996713	-0.160580
H	-3.950960	-5.098098	-0.524702
H	-5.050516	-1.997605	0.010487
C	-8.191516	2.145113	-2.078533
C	-8.391098	1.150828	-2.716354
H	-7.986254	3.029592	-1.514062

H	-8.554893	0.257502	-3.275644
C	-7.516537	4.543708	-4.645266
C	-6.922964	5.513914	-5.018748
H	-8.034755	3.673730	-4.305290
H	-6.397429	6.377898	-5.355741
C	-4.416043	2.895612	-3.440161
C	-3.638123	2.101630	-2.992244
H	-5.107513	3.612759	-3.826986
H	-2.953281	1.386851	-2.588451
C	-6.504377	5.324754	-0.993570
C	-5.809776	4.410581	-0.653246
H	-7.114587	6.141482	-1.304925
H	-5.190629	3.587556	-0.368955
C	-5.943103	-1.906214	-3.243061
C	-5.647604	-0.785490	-3.546500
H	-6.193011	-2.903664	-2.960457
H	-5.372353	0.217514	-3.796450

DEC 11

0 1

C	0.038294	1.664626	-0.063768
C	1.055945	1.267917	-0.555205
H	-0.883129	2.005146	0.357801
H	1.954395	0.914510	-1.008571
C	1.200264	-0.582225	-3.491721
C	2.079747	-0.027815	-4.085387
H	0.406021	-1.064608	-2.963471
H	2.861569	0.461436	-4.619576
C	-2.159768	-0.918318	2.253928
C	-1.043156	-0.526311	2.440382
H	-3.155959	-1.260163	2.065653
H	-0.047857	-0.176657	2.595981
C	-3.466627	2.304936	-0.386800
C	-3.653271	1.920386	0.732948
H	-3.283262	2.643104	-1.383284
H	-3.832585	1.575698	1.728975
C	-5.714986	-1.750099	1.242896
C	-5.115720	-1.519177	0.231756
H	-6.249435	-1.944856	2.144520
H	-4.583199	-1.302068	-0.669053
C	-2.320031	2.695803	-4.134471
C	-1.499665	2.141848	-3.459621
H	-3.044070	3.189001	-4.742468
H	-0.761372	1.659649	-2.854639
C	-8.395259	1.656761	0.745970
C	-7.285996	1.562058	0.304256
H	-9.385484	1.743752	1.130448
H	-6.294709	1.478583	-0.087960
C	-6.136758	0.141715	-2.796417
C	-5.013912	0.461837	-3.062673
H	-7.134334	-0.139318	-2.544761
H	-4.010905	0.750555	-3.291965
C	-5.689184	0.896298	3.525733
C	-4.760403	0.600889	4.221810
H	-6.506480	1.160050	2.888626
H	-3.928538	0.336535	4.834475
C	-1.838617	-1.204291	-1.373206
C	-2.238087	-1.596946	-2.432512
H	-1.489029	-0.858980	-0.423035
H	-2.601614	-1.945953	-3.372549

DEC 12

0 1

C	2.416750	3.022301	2.584205
C	2.507686	2.673992	3.726509
H	2.324253	3.320294	1.561345
H	2.587081	2.376581	4.747219
C	-1.050267	-0.155437	1.209615
C	-0.217476	0.514845	1.751081
H	-1.787547	-0.754112	0.717717
H	0.516046	1.122386	2.235124
C	-2.917954	-2.883549	-0.657910
C	-2.766822	-1.994862	-1.447105
H	-3.053095	-3.672781	0.050003
H	-2.622917	-1.190896	-2.135432
C	-1.883122	1.581355	-1.841520
C	-2.917532	1.738688	-2.425466
H	-0.960438	1.440641	-1.318693
H	-3.840719	1.874792	-2.941022
C	-3.474065	-5.369723	2.316928
C	-2.353665	-4.948448	2.293543
H	-4.470838	-5.746022	2.347476
H	-1.354945	-4.570390	2.257317
C	3.372426	-0.958394	0.373837
C	3.581235	-0.183070	1.262330
H	3.174306	-1.646480	-0.417123
H	3.757508	0.518151	2.047671
C	-1.461169	5.269717	-2.097437
C	-0.969792	6.361476	-2.106499
H	-1.890432	4.290433	-2.087061
H	-0.544016	7.338471	-2.116556
C	0.803062	-3.178795	1.107270
C	0.872657	-4.286730	0.656657
H	0.736788	-2.181269	1.488377
H	0.937245	-5.274043	0.259274
C	1.343260	3.290919	-1.154556
C	1.794553	2.227684	-0.833806
H	0.921936	4.228918	-1.445891
H	2.193223	1.279075	-0.540360
C	0.601826	-1.432561	-2.063300
C	1.001793	-0.540501	-2.755226
H	0.238258	-2.225705	-1.447000
H	1.350060	0.262489	-3.364503

DEC 13
0 1

C	-3.929926	-0.628092	-1.730362
C	-3.114600	-1.505128	-1.726938
H	-4.651165	0.157159	-1.738237
H	-2.374692	-2.276467	-1.716921
C	1.021953	0.216966	-1.327659
C	-0.161646	0.392676	-1.257599
H	2.075184	0.048844	-1.385950
H	-1.218371	0.538760	-1.201021
C	4.401290	1.147229	0.282380
C	3.622947	2.046345	0.426339
H	5.081192	0.335801	0.151593
H	2.922333	2.842809	0.553502
C	0.805244	3.907576	-1.059511
C	0.930503	5.039111	-0.687279
H	0.696163	2.889751	-1.372147
H	1.046252	6.047080	-0.360453
C	4.333008	-1.959934	-1.853546
C	3.546976	-2.333073	-1.030790
H	5.029360	-1.636748	-2.593078

H	2.838046	-2.658999	-0.300709
C	-0.173746	-3.155953	-0.320594
C	-0.129662	-4.199559	-0.907062
H	-0.211152	-2.222451	0.198944
H	-0.084377	-5.127763	-1.428980
C	-2.621677	3.188328	-0.329381
C	-2.877351	2.228409	0.340056
H	-2.381669	4.042858	-0.920764
H	-3.075637	1.362811	0.935129
C	-2.526074	-1.251706	1.773562
C	-1.610431	-0.602185	2.192403
H	-3.334893	-1.829016	1.386492
H	-0.788470	-0.021018	2.552518
C	1.050877	2.119536	3.097218
C	0.295397	2.492067	2.245506
H	1.727292	1.786448	3.851226
H	-0.382672	2.820704	1.487262
C	1.657960	-2.072428	2.520655
C	2.113824	-1.071795	2.045281
H	1.243091	-2.961647	2.937899
H	2.520726	-0.178762	1.619592

DEC 14
0 1

C	0.133098	1.341688	0.351361
C	1.308420	1.132469	0.454927
H	-0.910010	1.541738	0.256279
H	2.361302	0.958040	0.534866
C	1.944800	0.636456	-3.107754
C	2.554695	0.362329	-4.101388
H	1.413008	0.889456	-2.215721
H	3.095210	0.129745	-4.990367
C	1.563587	4.391610	2.073718
C	1.885291	5.543284	2.141058
H	1.287796	3.361337	1.999979
H	2.185440	6.564381	2.200900
C	4.674962	0.407001	-0.784699
C	5.100152	0.723779	0.290001
H	4.284991	0.145773	-1.745185
H	5.462271	1.014797	1.250638
C	5.284728	4.274282	1.177707
C	6.208133	4.363556	1.935430
H	4.460784	4.194502	0.499367
H	7.033755	4.448811	2.604242
C	2.840652	3.580297	-5.536978
C	3.178237	3.713317	-6.678310
H	2.547615	3.457357	-4.516266
H	3.467525	3.839405	-7.696356
C	6.524309	1.786455	-3.333087
C	5.919500	2.236927	-4.263484
H	7.050112	1.384217	-2.496747
H	5.367889	2.648038	-5.081164
C	-1.079483	4.803894	-0.287821
C	-1.399174	4.456316	-1.387970
H	-0.774172	5.110965	0.687994
H	-1.685949	4.152692	-2.369183
C	2.433480	4.034167	-1.505379
C	3.479009	3.601468	-1.901376
H	1.495264	4.405345	-1.153844
H	4.405927	3.204184	-2.257456
C	3.199713	0.891350	3.594007
C	3.648089	1.993433	3.458314
H	2.797757	-0.088477	3.715411

H	4.045851	2.974856	3.317279

DEC 15

0 1

C	-0.383005	4.727688	1.844644
C	-0.382027	3.767114	1.129426
H	-0.396910	5.582206	2.481486
H	-0.376285	2.911716	0.487745
C	-5.592004	0.137326	-0.223086
C	-6.134285	0.297529	0.832139
H	-5.097391	-0.005023	-1.159536
H	-6.624717	0.438683	1.767937
C	3.208457	3.529783	1.057908
C	4.222255	3.010198	0.686678
H	2.293711	3.984125	1.374339
H	5.114019	2.535224	0.343523
C	-2.271723	0.325598	1.204007
C	-1.106088	0.061743	1.296164
H	-3.312391	0.554011	1.117463
H	-0.062128	-0.161030	1.370855
C	2.601733	-0.193820	1.542418
C	2.357607	-1.362285	1.435358
H	2.827166	0.848389	1.622161
H	2.139024	-2.404852	1.339663
C	-2.495251	-3.200815	0.304434
C	-2.681926	-4.179034	-0.361537
H	-2.328068	-2.315603	0.882738
H	-2.854507	-5.054470	-0.945660
C	0.921103	-4.763507	0.749358
C	2.049274	-5.102301	0.963622
H	-0.087229	-4.465693	0.556112
H	3.051853	-5.411631	1.152096
C	5.408837	0.684676	-2.168582
C	4.565163	0.160216	-1.500178
H	6.157351	1.144137	-2.772261
H	3.809548	-0.302757	-0.902331
C	-3.032236	0.111655	-3.125574
C	-2.891117	-0.895823	-2.493391
H	-3.148623	1.007796	-3.691900
H	-2.766428	-1.793966	-1.926667
C	0.297974	1.146360	-1.669506
C	1.323615	1.746014	-1.514911
H	-0.617031	0.612737	-1.808561
H	2.240557	2.273621	-1.370066

DEC16

0 1

C	-0.938110	2.179200	0.797943
C	0.139332	1.851158	0.388076
H	-1.903646	2.472428	1.151596
H	1.103651	1.566729	0.028159
C	1.164724	3.060758	-2.724310
C	1.233340	2.075190	-3.401769
H	1.092252	3.936984	-2.115423
H	1.281252	1.199952	-4.008373
C	1.701725	3.246531	3.282604
C	2.608434	3.878340	3.745062
H	0.891661	2.692172	2.856465
H	3.412308	4.440372	4.162656
C	3.792990	1.910065	-0.468124
C	3.868221	1.757255	0.717418
H	3.703927	2.053397	-1.522407

H	3.925990	1.631730	1.775363
C	-3.714576	1.717026	-2.916402
C	-2.617498	1.900702	-2.472938
H	-4.690500	1.546155	-3.308507
H	-1.641220	2.068354	-2.071625
C	-2.271483	1.813996	4.463212
C	-1.922658	2.938948	4.247643
H	-2.573861	0.810308	4.656632
H	-1.611191	3.939128	4.036223
C	2.273707	5.188699	0.104189
C	1.767028	6.169697	-0.360022
H	2.725756	4.310904	0.512669
H	1.306838	7.037733	-0.774409
C	-1.787835	6.075481	2.341815
C	-0.602444	5.918317	2.418238
H	-2.843846	6.201086	2.255635
H	0.453901	5.767705	2.472131
C	-1.738164	5.225612	-2.370492
C	-1.481074	5.320916	-1.204432
H	-1.966971	5.120788	-3.406250
H	-1.251803	5.396997	-0.162862
C	-4.195864	3.687710	0.483830
C	-4.397928	3.523328	1.653149
H	-3.995108	3.816612	-0.557909
H	-4.563570	3.362296	2.694460

DEC 17

0 1

C	0.597204	0.893690	0.123884
C	1.743302	0.583182	0.285844
H	-0.425159	1.169677	-0.025508
H	2.768167	0.318284	0.416024
C	0.944188	2.660975	-3.006151
C	0.119854	3.220817	-3.670631
H	1.678802	2.160565	-2.412848
H	-0.613343	3.720272	-4.261942
C	0.621531	2.337947	3.441744
C	1.147688	3.342110	3.829386
H	0.149211	1.448109	3.085410
H	1.607266	4.245625	4.161588
C	4.343463	2.235841	-1.426873
C	4.727252	1.294088	-2.058950
H	3.985956	3.074182	-0.867877
H	5.073324	0.460029	-2.625164
C	-2.898941	0.942107	-1.294739
C	-2.185949	0.715509	-2.229423
H	-3.516126	1.148892	-0.449787
H	-1.540520	0.532203	-3.058327
C	-3.711657	4.040776	4.483964
C	-2.517860	3.949299	4.447178
H	-4.773183	4.130466	4.525667
H	-1.452768	3.861149	4.395001
C	2.535110	5.303010	-0.085294
C	2.055471	4.360722	0.478497
H	2.956386	6.143307	-0.588947
H	1.619304	3.523966	0.982374
C	-1.335681	3.943563	-0.188230
C	-1.863827	4.181191	0.860681
H	-0.859360	3.728182	-1.120736
H	-2.329338	4.406064	1.794996
C	-2.583048	0.766642	2.624132
C	-2.170390	-0.182204	2.020826

H	-2.942031	1.614102	3.167794
H	-1.803787	-1.022046	1.474694
C	-0.467136	6.874141	3.774454
C	-0.012038	6.465114	2.745134
H	-0.880487	7.239388	4.686567
H	0.386370	6.091999	1.826160

DEC 18

0 1

C	0.844917	-1.335036	0.047246
C	-0.144087	-1.472864	0.710259
H	1.730395	-1.203287	-0.539072
H	-1.023420	-1.600011	1.305063
C	-1.432645	-0.952341	-2.805596
C	-1.930960	-0.587843	-3.832125
H	-0.979276	-1.268064	-1.888867
H	-2.370312	-0.271747	-4.750881
C	2.317240	1.483528	1.590806
C	3.373561	1.983985	1.851711
H	1.380269	1.034346	1.343179
H	4.312502	2.440031	2.068804
C	-3.601787	-1.655814	2.016941
C	-3.168145	-2.005862	3.077265
H	-3.996704	-1.338873	1.073425
H	-2.780510	-2.319581	4.019798
C	4.937230	-2.719864	1.733488
C	4.345608	-1.683643	1.834595
H	5.458568	-3.646219	1.656090
H	3.818124	-0.757243	1.923858
C	5.068620	2.869402	-1.054533
C	5.203723	4.053027	-0.934961
H	4.936674	1.814388	-1.165281
H	5.330036	5.106400	-0.833153
C	3.501184	-0.204700	-2.296213
C	4.188510	-0.893891	-1.597659
H	2.879603	0.421370	-2.897817
H	4.784033	-1.505488	-0.956450
C	-7.003681	-0.620982	2.882154
C	-8.092921	-0.197091	2.624114
H	-6.028848	-0.995114	3.107783
H	-9.067033	0.176526	2.405753
C	-4.826909	-0.405242	-1.311694
C	-5.739288	-0.288850	-0.544358
H	-4.012474	-0.507063	-1.996303
H	-6.542984	-0.188868	0.152389
C	0.488758	2.046274	-2.077521
C	1.431781	2.604017	-1.593223
H	-0.350378	1.543818	-2.505741
H	2.276909	3.089452	-1.155866

DEC 19

0 1

C	1.111187	-0.497885	-0.890557
C	0.257322	0.306747	-1.137636
H	1.866721	-1.221464	-0.666369
H	-0.512667	1.021173	-1.338742
C	3.777117	1.898671	-1.337462
C	4.896254	2.262928	-1.116075
H	2.776787	1.566280	-1.516440
H	5.894362	2.586219	-0.927707
C	-2.469424	-1.508190	0.144044

C	-3.198937	-2.163174	-0.544394
H	-1.816672	-0.918686	0.753044
H	-3.832244	-2.755533	-1.164757
C	1.372857	4.178994	-0.343348
C	0.602520	3.558712	0.331857
H	2.073254	4.719923	-0.937617
H	-0.078655	2.994376	0.932225
C	-0.234445	-4.560911	-2.957708
C	-0.389647	-3.488297	-2.448338
H	-0.102461	-5.513367	-3.417302
H	-0.529585	-2.531169	-1.993136
C	3.041418	-0.602563	2.665259
C	3.306257	0.273781	1.892812
H	2.804038	-1.383047	3.352042
H	3.545146	1.049103	1.196202
C	2.761861	-3.687744	-0.567126
C	3.611433	-3.126312	0.062899
H	1.994499	-4.168000	-1.134654
H	4.360424	-2.616104	0.624874
C	-3.048437	1.983399	-0.972944
C	-2.429747	2.984943	-1.193856
H	-3.597898	1.090905	-0.767937
H	-1.866265	3.871079	-1.381064
C	-0.438530	0.817073	2.398345
C	-1.592127	1.139092	2.443006
H	0.593305	0.539305	2.347586
H	-2.625974	1.407181	2.470102
C	-5.415398	1.440483	1.756894
C	-5.116837	0.281176	1.767083
H	-5.684741	2.472159	1.744638
H	-4.840285	-0.749532	1.757893

DEC 20

0 1

C	-0.274663	1.945110	1.344550
C	0.674526	1.327346	1.734308
H	-1.114344	2.507155	0.996175
H	1.527087	0.783917	2.074257
C	0.926345	4.394264	-6.043908
C	1.512634	3.650816	-6.776415
H	0.407064	5.053127	-5.382121
H	2.034818	2.996190	-7.436160
C	1.566049	4.082993	3.926995
C	1.740211	3.700748	5.048854
H	1.417711	4.416907	2.920956
H	1.889255	3.369222	6.051114
C	4.597201	2.000122	1.044952
C	4.114753	2.380249	2.073730
H	5.032261	1.667822	0.126624
H	3.680886	2.724574	2.987482
C	-3.197236	3.124900	4.575416
C	-2.013360	3.061868	4.406555
H	-4.250539	3.173074	4.731694
H	-0.956121	3.006018	4.258833
C	4.984104	1.500033	-2.524413
C	6.174758	1.391683	-2.459892
H	3.921300	1.607010	-2.577988
H	7.235228	1.296877	-2.411272
C	-2.656439	4.725089	1.178831
C	-2.501023	4.901538	0.003979
H	-2.789065	4.548992	2.224993
H	-2.351421	5.067002	-1.040598

C	1.418263	2.406341	-3.067346
C	1.346457	2.221863	-1.884984
H	1.471658	2.585432	-4.120110
H	1.260990	2.056938	-0.830991
C	-0.955046	6.465562	-3.235930
C	-0.242626	5.612629	-2.788297
H	-1.583548	7.231628	-3.629744
H	0.392559	4.850231	-2.387779
C	1.057349	5.521952	0.471398
C	2.095776	4.929377	0.395690
H	0.124344	6.036799	0.532097
H	3.016485	4.390034	0.343220

EICO 1

0 1

C	4.003288	4.769306	10.739661
C	4.991851	5.385470	11.021343
H	3.124578	4.222654	10.475621
H	5.882196	5.929618	11.251014
C	2.371399	8.966396	10.801754
C	2.420419	8.048350	11.569676
H	2.342909	9.783730	10.112753
H	2.476723	7.226375	12.246655
C	8.412986	7.227034	10.734827
C	8.188338	6.383343	9.914545
H	8.617328	7.987210	11.454209
H	7.977660	5.629824	9.184929
C	7.203601	3.581505	7.496350
C	6.736850	3.222372	8.540187
H	7.590767	3.915061	6.557731
H	6.294417	2.917324	9.462778
C	0.749266	4.568399	8.602834
C	0.955124	5.648050	9.078402
H	0.565219	3.609434	8.175025
H	1.147901	6.608827	9.504211
C	6.685641	9.815259	8.856993
C	6.446888	10.980325	8.716408
H	6.917311	8.778822	8.973285
H	6.225392	12.014921	8.586317
C	2.752732	3.525025	1.368561
C	3.644952	3.491153	2.166947
H	1.961677	3.555665	0.653996
H	4.451173	3.475233	2.868316
C	5.874494	0.235196	4.566777
C	6.285253	0.613994	5.626017
H	5.506850	-0.112450	3.628093
H	6.637886	0.969391	6.570063
C	6.713278	6.620252	-0.182531
C	5.861717	5.936391	0.307494
H	7.471065	7.234169	-0.612772
H	5.095518	5.335520	0.745991
C	3.447820	1.812880	8.886043
C	3.297684	1.069374	9.813209
H	3.586107	2.480599	8.060561
H	3.159613	0.405477	10.635804
C	3.491838	7.921647	6.910721
C	3.692502	7.493053	8.012636
H	3.330876	8.309561	5.927016
H	3.870459	7.103947	8.992586
C	8.184431	9.097920	2.959276
C	8.262658	7.931247	2.700712

H	8.102308	10.134646	3.195841
H	8.316078	6.890928	2.465929
C	3.004661	11.302819	7.991537
C	2.165712	12.064946	8.380715
H	3.755479	10.620581	7.650872
H	1.427296	12.750607	8.729411
C	7.970439	7.208368	6.250109
C	6.851416	6.840304	6.470689
H	8.963277	7.538103	6.042883
H	5.848032	6.525282	6.666345
C	4.654833	7.524864	3.187067
C	3.769518	6.773728	3.486711
H	5.454732	8.181831	2.921957
H	2.969861	6.116594	3.754855
C	1.491609	9.491822	4.035643
C	1.267929	10.040869	5.076575
H	1.706744	8.993657	3.116722
H	1.097883	10.530293	6.010606
C	4.131162	3.508878	5.771312
C	3.429389	4.375711	6.211820
H	4.762241	2.731217	5.396916
H	2.809007	5.149105	6.612241
C	6.774090	4.565065	3.749363
C	7.373164	3.536395	3.611605
H	6.223619	5.474445	3.867465
H	7.896991	2.615210	3.495931
C	0.395419	6.089767	4.794456
C	0.641676	5.006121	4.347730
H	0.190919	7.060794	5.188466
H	0.885056	4.044007	3.955375
C	4.881732	10.927928	4.784271
C	5.669704	10.194703	5.310478
H	4.164177	11.568328	4.324101
H	6.355399	9.524992	5.783537

EICO 2

0 1

C	3.865470	4.523555	11.118779
C	4.921625	5.040873	11.349163
H	2.923807	4.074175	10.890331
H	5.865110	5.508408	11.527342
C	2.909731	8.809619	11.008267
C	2.837147	7.970926	11.860358
H	2.978845	9.552349	10.243233
H	2.785227	7.214786	12.609786
C	7.998284	7.176682	10.517253
C	7.718248	6.459970	9.598957
H	8.251318	7.825291	11.324947
H	7.463509	5.821996	8.778731
C	6.128036	4.196057	6.900359
C	5.720696	3.870808	7.980051
H	6.491331	4.473857	5.933392
H	5.345345	3.598153	8.942438
C	0.426757	4.671684	9.345786
C	0.884500	5.738359	9.642554
H	0.022277	3.721889	9.077850
H	1.303007	6.684433	9.914402
C	6.117612	9.960893	9.195349
C	5.825796	11.115920	9.318555
H	6.386553	8.931498	9.094497
H	5.557342	12.141785	9.428421
C	2.936119	3.517482	1.461841

C	4.031881	3.638195	1.930802
H	1.961675	3.411713	1.042919
H	5.010480	3.756717	2.348212
C	4.552372	0.869409	4.115307
C	4.791968	1.299046	5.207490
H	4.334074	0.495843	3.140796
H	5.005888	1.698779	6.175566
C	7.023918	6.387400	0.117870
C	5.860083	6.263726	0.372024
H	8.064961	6.491724	-0.089052
H	4.829077	6.134535	0.617459
C	2.824253	2.022382	8.548566
C	3.059218	1.302779	9.476902
H	2.621846	2.666957	7.718229
H	3.270235	0.659463	10.300477
C	3.483526	7.729389	7.247486
C	3.853767	7.099571	8.198420
H	3.153227	8.282497	6.393854
H	4.168361	6.539121	9.053346
C	9.212181	7.957212	2.489695
C	9.999676	7.063812	2.616475
H	8.501273	8.747394	2.373027
H	10.694419	6.263364	2.733264
C	2.548297	11.211617	8.080087
C	1.581167	11.552901	8.699286
H	3.415643	10.908697	7.532105
H	0.724567	11.857131	9.256184
C	8.129277	7.697444	5.919141
C	6.971759	7.527805	6.178864
H	9.156181	7.842574	5.672134
H	5.938755	7.375683	6.412793
C	4.750515	7.521907	3.502481
C	4.158756	6.558930	3.902771
H	5.282779	8.381026	3.154179
H	3.627969	5.708909	4.277319
C	1.491960	9.020438	4.322001
C	1.049318	9.717922	5.189429
H	1.906797	8.390034	3.566818
H	0.671319	10.334067	5.974677
C	6.436338	10.641171	2.059001
C	6.352915	9.849075	1.164086
H	6.489395	11.337825	2.865637
H	6.277992	9.121412	0.385664
C	7.392979	4.629956	3.395456
C	7.314278	3.452400	3.604524
H	7.466849	5.679571	3.209366
H	7.221833	2.407217	3.799524
C	1.907490	4.607981	6.053081
C	2.151884	3.729831	5.274269
H	1.702355	5.385845	6.757223
H	2.394961	2.953386	4.582366
C	4.520868	10.923974	5.026510
C	5.499396	10.678624	5.673212
H	3.639150	11.119803	4.458034
H	6.361957	10.443852	6.257233

EICO 3

0 1

C	4.162118	5.932192	10.627904
C	5.266015	6.231743	11.013961
H	3.191992	5.669434	10.262347
H	6.254660	6.504739	11.312728

C	1.022392	8.440920	9.767693
C	2.026691	8.612982	10.414405
H	0.150105	8.285268	9.170075
H	2.929588	8.741636	10.968699
C	8.858111	7.772875	10.432590
C	8.598854	6.848114	9.700937
H	9.103328	8.588151	11.075827
H	8.375297	6.021053	9.056352
C	8.793227	3.899355	7.471418
C	7.877598	3.424917	8.098418
H	9.597696	4.316226	6.903732
H	7.060341	2.997830	8.641051
C	1.848870	3.653041	8.867980
C	1.270509	4.513622	8.249061
H	2.388391	2.900031	9.401574
H	0.761173	5.269309	7.690350
C	5.877886	9.496968	8.802755
C	4.926524	10.212509	8.599889
H	6.719232	8.862996	8.989580
H	4.069807	10.821562	8.401775
C	-0.116852	4.762045	4.376430
C	0.797056	3.974645	4.347761
H	-0.917504	5.465364	4.435876
H	1.625584	3.300361	4.340014
C	4.711273	-1.252746	6.879594
C	4.677899	-0.118548	6.469588
H	4.741951	-2.262047	7.224342
H	4.645048	0.890336	6.110500
C	6.907496	8.551570	2.228278
C	7.324877	7.786863	1.392997
H	6.529344	9.227462	2.967309
H	7.707552	7.105123	0.666986
C	4.921951	1.459499	9.684634
C	4.980236	2.454576	10.366487
H	4.875874	0.592790	9.058552
H	5.022329	3.356263	10.940557
C	3.112667	7.017774	5.704277
C	3.087178	7.523694	6.801008
H	3.146743	6.573970	4.732397
H	3.052617	7.967852	7.772886
C	11.665298	5.825008	5.476631
C	10.860952	5.372470	4.698942
H	12.388882	6.220660	6.153772
H	10.138151	4.972669	4.017744
C	1.961954	11.165436	6.668292
C	1.349245	11.336352	7.693971
H	2.512261	10.995217	5.767069
H	0.811266	11.472627	8.605062
C	7.960619	7.382641	5.794748
C	6.780136	7.243164	6.008546
H	9.008937	7.469889	5.609660
H	5.732342	7.129892	6.200307
C	3.766410	6.588444	1.905654
C	2.721709	6.018754	2.112927
H	4.700705	7.081067	1.726993
H	1.807194	5.507673	2.329800
C	0.585570	10.218123	3.574224
C	1.320096	9.355106	3.159540
H	-0.057989	10.982548	3.947707
H	1.959697	8.581510	2.788583
C	4.754750	3.359571	4.920059
C	4.017223	3.535492	5.860476
H	5.438438	3.250063	4.105848

H	3.361131	3.709997	6.689072
C	7.621746	4.755770	3.162274
C	7.944643	3.806235	2.489888
H	7.348079	5.597127	3.766727
H	8.223199	2.966175	1.893758
C	-1.555837	7.256694	7.024783
C	-0.607249	7.776515	6.487821
H	-2.401929	6.802844	7.490587
H	0.236259	8.234744	6.014596
C	4.740102	11.025255	3.946914
C	5.202953	10.562558	4.961311
H	4.320677	11.431916	3.054032
H	5.595237	10.157824	5.871014

EICO 4

0 1

C	1.594720	2.176830	2.917653
C	2.528469	1.776487	2.265569
H	0.754154	2.505331	3.490289
H	3.359708	1.424132	1.692317
C	5.710317	2.493602	0.583227
C	5.560589	1.698308	-0.313081
H	5.833559	3.187609	1.389132
H	5.443851	0.997838	-1.113488
C	-0.695231	2.970987	0.305799
C	-1.847066	2.725186	0.575475
H	0.317986	3.202459	0.048450
H	-2.854193	2.478287	0.838470
C	7.446772	-3.316367	0.335300
C	7.022888	-2.695149	-0.607903
H	7.834663	-3.865700	1.163700
H	6.640059	-2.145692	-1.442629
C	-5.037936	0.866058	1.811564
C	-4.419904	1.273101	2.765944
H	-5.578415	0.494708	0.965645
H	-3.858519	1.634716	3.601831
C	-1.469011	1.563879	4.880843
C	-1.708737	2.586569	5.475120
H	-1.257911	0.667271	4.335450
H	-1.912083	3.486364	6.011830
C	4.771516	4.354135	3.531897
C	5.789673	4.984800	3.677205
H	3.875252	3.785579	3.388964
H	6.684217	5.548613	3.819740
C	2.834515	-1.697773	1.674429
C	3.955884	-2.013319	1.355415
H	1.846445	-1.403130	1.961013
H	4.948807	-2.295545	1.071783
C	-4.308524	3.224340	-2.948788
C	-5.044549	2.292446	-2.733364
H	-3.664940	4.059461	-3.127078
H	-5.684131	1.459736	-2.530892
C	-1.745028	5.562450	-2.006464
C	-1.733661	6.327133	-2.940247
H	-1.742484	4.873595	-1.185916
H	-1.729832	7.011348	-3.759223
C	-0.830675	-1.539137	2.738330
C	-0.852228	-0.598707	1.980705
H	-0.819786	-2.378226	3.398032
H	-0.877110	0.236510	1.310980
C	2.746094	4.088959	-1.250819
C	1.810527	4.679247	-1.733311

H 3.584686 3.568352 -0.837540
H 0.974226 5.198550 -2.150309
C -0.500890 -2.311812 -6.246207
C -1.001868 -1.804531 -5.272871
H -0.076441 -2.748349 -7.122384
H -1.436439 -1.363109 -4.399530
C -2.313848 -0.030158 -2.054716
C -1.850912 -1.085223 -1.690580
H -2.742061 0.903746 -2.355597
H -1.424766 -2.006821 -1.350999
C 2.060000 -2.571827 -1.826778
C 1.598281 -2.588399 -2.942881
H 2.459250 -2.538080 -0.832997
H 1.152082 -2.586306 -3.915780
C 6.052515 -0.334169 -3.456979
C 4.977688 -0.837695 -3.235056
H 6.998090 0.112500 -3.671072
H 4.023875 -1.288228 -3.044317
C -1.455488 -4.158201 0.249383
C -0.646945 -4.555744 -0.554462
H -2.178303 -3.808439 0.958771
H 0.089940 -4.858999 -1.265024
C -4.283659 -7.801805 1.406515
C -3.450737 -7.144010 0.833190
H -5.014388 -8.398459 1.904853
H -2.707773 -6.548913 0.344806
C -5.741415 -1.003024 -1.314533
C -6.922308 -0.791265 -1.181348
H -4.690811 -1.168459 -1.441956
H -7.971524 -0.631600 -1.068093
C -4.240627 -2.774415 2.513523
C -4.342418 -3.973647 2.423656
H -4.169802 -1.709136 2.579131
H -4.424456 -5.035155 2.320824

EICO 5

0 1
C 4.622980 4.178759 11.942645
C 5.647477 4.816179 11.907884
H 3.718570 3.611497 11.948028
H 6.550999 5.385512 11.838592
C 3.007125 8.872901 11.330570
C 3.509208 7.888502 11.815787
H 2.560392 9.733738 10.880261
H 3.954525 7.008200 12.227617
C 8.507301 7.247699 10.688745
C 8.050933 6.544920 9.819867
H 8.918951 7.879674 11.443532
H 7.636489 5.925387 9.051104
C 6.478723 3.951971 7.343853
C 6.144332 3.747220 8.486072
H 6.748823 4.115123 6.322217
H 5.834939 3.606330 9.499654
C 2.595365 1.582372 4.005710
C 2.603499 1.623007 5.212021
H 2.611411 1.576136 2.937327
H 2.617749 1.667674 6.280636
C 7.827996 10.373553 8.747473
C 7.985866 11.555623 8.931943
H 7.683999 9.326056 8.583084
H 8.134523 12.597575 9.106674
C 3.032797 3.223169 0.435099

C	3.956655	3.282603	1.209560
H	2.228981	3.172991	-0.264774
H	4.773748	3.326422	1.901039
C	5.461763	-0.624553	6.319087
C	5.675988	0.401678	6.916220
H	5.269205	-1.536347	5.799660
H	5.863270	1.324446	7.425875
C	7.633152	5.975974	0.227180
C	6.452181	6.078518	0.453770
H	8.677802	5.876226	0.034592
H	5.406239	6.172245	0.659650
C	2.621093	3.087352	8.792480
C	2.629879	1.924483	9.117005
H	2.614169	4.119717	8.505374
H	2.638016	0.895385	9.399342
C	2.433729	6.560750	7.158465
C	2.770480	6.907744	8.265579
H	2.111210	6.255577	6.184382
H	3.048742	7.234121	9.247054
C	9.199946	6.993749	3.285124
C	10.041754	6.212443	3.656348
H	8.446245	7.686504	2.971750
H	10.791418	5.523968	3.976778
C	1.816686	11.029585	8.450246
C	0.758020	11.092005	9.026779
H	2.748865	10.978624	7.925158
H	-0.176374	11.149327	9.539116
C	7.326991	7.511474	6.230165
C	6.176043	7.504534	6.595143
H	8.333264	7.495935	5.872705
H	5.153763	7.483853	6.910799
C	3.498369	7.860494	1.621207
C	2.789392	6.891826	1.751308
H	4.160286	8.695967	1.538058
H	2.169769	6.033370	1.896100
C	1.137665	9.314341	4.070496
C	0.746864	9.669932	5.156150
H	1.501279	8.964239	3.126925
H	0.436924	9.993718	6.126882
C	6.465567	9.769873	2.887250
C	6.825202	9.458965	1.777203
H	6.134981	10.046923	3.867609
H	7.138688	9.150102	0.804439
C	7.022218	3.655453	3.434581
C	6.513882	2.681098	3.935956
H	7.475318	4.514525	2.986799
H	6.069766	1.823489	4.397217
C	0.249902	5.846573	4.250407
C	0.731318	4.739324	4.263447
H	-0.150579	6.837047	4.256438
H	1.160700	3.758584	4.293922
C	4.180345	10.837980	5.700605
C	5.167035	11.016400	6.374284
H	3.295679	10.656667	5.126304
H	6.039397	11.157469	6.979507

EICO 6

0 1

C	3.388059	4.853993	-1.244800
C	4.002528	4.811267	-0.206784
H	2.818642	4.875055	-2.148491

H	4.523805	4.743590	0.723324
C	5.427757	0.199750	-0.829340
C	5.094517	1.350271	-0.979221
H	5.730177	-0.817664	-0.693960
H	4.798955	2.371267	-1.097323
C	4.740722	3.126238	3.353405
C	3.614012	2.995871	2.940167
H	5.734486	3.235777	3.726245
H	2.613715	2.889108	2.574250
C	-0.131380	3.417347	1.642429
C	0.664488	4.226264	1.229400
H	-0.852325	2.702521	1.978860
H	1.393048	4.909013	0.848121
C	-4.679585	2.127407	-2.780683
C	-3.600379	2.654109	-2.904235
H	-5.620822	1.643182	-2.636735
H	-2.641977	3.119438	-2.999527
C	5.258231	-0.607788	2.876057
C	5.852236	-1.650045	2.745781
H	4.744736	0.324192	2.984786
H	6.377253	-2.566921	2.595902
C	-6.864605	-0.811347	-1.174718
C	-6.011881	-0.250895	-0.529852
H	-7.622198	-1.311949	-1.735057
H	-5.255282	0.249252	0.040684
C	-3.637177	5.603039	-0.932935
C	-2.595321	5.351279	-0.378836
H	-4.555292	5.827473	-1.427558
H	-1.679220	5.103898	0.115638
C	-7.044237	-2.674635	3.754103
C	-6.506809	-2.557969	2.680350
H	-7.538674	-2.778133	4.693922
H	-6.026491	-2.456829	1.729988
C	0.289052	3.326865	-2.705162
C	-0.072381	4.420242	-3.067519
H	0.603633	2.354838	-2.382951
H	-0.401621	5.386732	-3.378220
C	0.601167	-0.367516	-1.778729
C	1.733912	-0.076490	-1.472995
H	-0.396417	-0.636773	-2.061832
H	2.743007	0.171808	-1.208405
C	-2.076216	-1.647348	4.062014
C	-3.249183	-1.896009	4.205396
H	-1.042277	-1.409074	3.923598
H	-4.292117	-2.117018	4.297677
C	5.732748	-3.583855	-0.395035
C	6.912139	-3.487762	-0.632187
H	4.687224	-3.658315	-0.175348
H	7.953494	-3.413006	-0.851977
C	1.142273	0.275041	3.616052
C	1.038386	-0.062119	2.461346
H	1.241709	0.579226	4.634203
H	0.962313	-0.364532	1.437822
C	-3.295473	-3.239847	0.891725
C	-3.648122	-2.905109	-0.213432
H	-2.983383	-3.485017	1.883317
H	-3.933582	-2.587802	-1.193058
C	-0.865714	-4.825676	-1.598808
C	0.073227	-4.873224	-2.356799
H	-1.701784	-4.750042	-0.935570
H	0.917276	-4.879078	-3.014076
C	-3.331181	1.064866	1.778906
C	-3.475779	2.018024	1.051133

H	-3.201950	0.219491	2.423316
H	-3.594158	2.863417	0.405638
C	-2.247333	-2.045445	-3.466020
C	-2.786401	-0.964919	-3.459503
H	-1.761842	-2.998141	-3.443714
H	-3.254793	-0.002033	-3.451801
C	1.944543	-3.477735	0.591580
C	2.674824	-2.958193	1.400678
H	1.283624	-3.932653	-0.116421
H	3.330088	-2.479644	2.098132
C	3.203458	-3.887623	-4.400838
C	2.717273	-2.975252	-3.778898
H	3.650191	-4.680674	-4.957464
H	2.285466	-2.167980	-3.224035

EICO 7

0 1

C	-1.762153	-3.090171	1.020188
C	-2.524349	-2.348032	0.448523
H	-1.069713	-3.715539	1.541565
H	-3.205918	-1.698408	-0.059219
C	-5.201826	-1.767261	-1.963717
C	-4.890135	-0.638342	-2.258547
H	-5.469480	-2.766424	-1.686524
H	-4.633418	0.363967	-2.531732
C	1.031976	-2.485248	-1.315931
C	2.083665	-2.371015	-0.732452
H	0.110146	-2.582435	-1.851742
H	2.997023	-2.257235	-0.186692
C	-7.323192	3.091347	0.635522
C	-6.670572	3.179741	-0.375240
H	-7.910309	3.021726	1.523989
H	-6.087859	3.251428	-1.269827
C	5.015130	-1.600338	1.840428
C	4.169144	-2.301459	2.341474
H	5.766573	-0.978819	1.399772
H	3.415535	-2.921919	2.779966
C	0.866547	-3.377770	3.556134
C	1.116854	-4.557589	3.603540
H	0.650413	-2.331176	3.488823
H	1.333070	-5.601042	3.661358
C	-4.971199	-4.947392	-0.218677
C	-5.970935	-5.413276	-0.707599
H	-4.088186	-4.515234	0.205178
H	-6.851494	-5.844134	-1.128708
C	-2.926810	0.963268	1.767638
C	-4.004739	1.404314	1.446425
H	-1.967148	0.572199	2.037857
H	-4.957538	1.795642	1.152704
C	5.467729	-1.426303	-3.549462
C	6.204838	-0.834941	-2.798874
H	4.818651	-1.966190	-4.206000
H	6.845088	-0.313873	-2.119765
C	2.677270	-3.573252	-4.341176
C	2.877119	-3.755141	-5.517561
H	2.493170	-3.398710	-3.300265
H	3.054349	-3.927563	-6.555722
C	0.680233	0.409301	2.813940
C	0.619262	-0.256930	1.808156
H	0.774019	1.015884	3.687148
H	0.581895	-0.845332	0.914762
C	-1.954172	-2.470644	-3.867843

C	-0.916533	-2.813968	-4.379641
H	-2.877868	-2.160579	-3.425543
H	0.008842	-3.113849	-4.823043
C	1.885714	5.281845	-3.731991
C	2.274750	4.329611	-3.101409
H	1.559962	6.123298	-4.301387
H	2.613561	3.484690	-2.537299
C	3.490873	1.126694	-1.352216
C	3.035760	1.773543	-0.438585
H	3.910240	0.533598	-2.138418
H	2.618913	2.334335	0.373845
C	-1.507761	3.581041	-0.375399
C	-0.775388	4.089338	-1.190411
H	-2.149431	3.104551	0.337280
H	-0.100521	4.512974	-1.905849
C	-4.923894	2.833818	-3.757053
C	-3.956604	3.071307	-3.074604
H	-5.771408	2.626909	-4.372301
H	-3.094626	3.292364	-2.477173
C	2.214341	3.701314	2.723277
C	1.364313	4.094893	1.962106
H	2.949949	3.323811	3.401028
H	0.606553	4.418543	1.280405
C	6.760253	1.224651	0.191769
C	7.856163	0.801885	0.470049
H	5.782320	1.580773	-0.061994
H	8.829685	0.445096	0.723184
C	3.788018	0.819514	4.617591
C	3.603137	1.583662	5.533322
H	3.951997	0.129642	3.815318
H	3.444709	2.247379	6.353759
C	-4.705949	0.940214	5.165473
C	-5.106578	0.926863	6.302493
H	-4.350131	0.953750	4.156689
H	-5.460525	0.913236	7.308566

EICO 8

0 1

C	-6.267227	5.552069	3.069243
C	-5.964783	5.541983	1.900708
H	-6.549310	5.570600	4.098351
H	-5.683696	5.527693	0.866971
C	-4.698520	5.099545	-1.790657
C	-3.828870	5.308523	-0.980018
H	-5.431663	4.872398	-2.532701
H	-3.064215	5.468773	-0.248586
C	-5.637079	2.335055	-4.548447
C	-4.747513	2.245232	-3.738816
H	-6.420303	2.402920	-5.269356
H	-3.960287	2.167382	-3.018100
C	1.220871	-0.388065	-2.725513
C	1.768057	0.673049	-2.906223
H	0.766576	-1.337709	-2.540155
H	2.273436	1.603907	-3.050155
C	1.737593	3.037612	-0.087166
C	1.604735	4.104798	-0.635044
H	1.861127	2.088182	0.392040
H	1.483820	5.049391	-1.116170
C	2.884188	-0.254302	1.447640
C	1.716781	-0.500639	1.635745
H	3.914733	-0.026638	1.278842
H	0.683190	-0.732449	1.786227

C	-1.613178	1.725510	-1.372994
C	-2.529018	2.020441	-0.642535
H	-0.788397	1.462572	-2.001689
H	-3.326523	2.271181	0.024423
C	-2.278157	5.101078	2.289066
C	-1.286640	4.698673	1.730710
H	-3.172605	5.439201	2.765704
H	-0.408510	4.334616	1.238994
C	-4.532047	0.852032	2.322911
C	-5.058461	1.936375	2.376139
H	-4.049900	-0.100011	2.271527
H	-5.523395	2.897697	2.434235
C	-5.287298	-6.563023	3.188450
C	-5.175631	-6.042402	2.105976
H	-5.402858	-7.022126	4.144663
H	-5.062314	-5.580805	1.147280
C	-3.867979	-4.067775	-0.916406
C	-4.556124	-4.830221	-1.550872
H	-3.262764	-3.383625	-0.357413
H	-5.164357	-5.498223	-2.119354
C	-1.759351	-2.204470	1.555119
C	-1.873133	-1.196233	0.898639
H	-1.646994	-3.124766	2.089611
H	-1.959600	-0.296633	0.321736
C	4.840871	-1.069207	-1.701938
C	5.949050	-1.007911	-1.226714
H	3.856912	-1.102916	-2.119854
H	6.924357	-0.952517	-0.791736
C	5.553222	2.359553	1.813004
C	5.354936	2.737084	0.683382
H	5.716486	1.991572	2.802584
H	5.160950	3.069543	-0.314704
C	6.501148	-0.688841	3.505857
C	5.443527	-0.714927	4.085690
H	7.427494	-0.659854	2.972924
H	4.505094	-0.750181	4.592525
C	9.380446	-0.306365	0.726888
C	8.552851	0.571879	0.756611
H	10.126882	-1.068566	0.694474
H	7.805019	1.338235	0.790320
C	4.791444	3.457868	-3.231182
C	5.097266	2.326482	-2.942901
H	4.534734	4.457564	-3.501536
H	5.357997	1.323702	-2.670857
C	-1.617289	-5.927605	1.927441
C	-0.636958	-5.532072	1.344584
H	-2.501531	-6.277444	2.416648
H	0.218376	-5.171898	0.812291
C	1.249338	-4.071866	-1.509860
C	1.748082	-3.303753	-0.722332
H	0.793269	-4.755319	-2.196466
H	2.178097	-2.607194	-0.032643
C	-1.344416	-6.036748	-3.144280
C	-0.528974	-6.525014	-3.887455
H	-2.071299	-5.603336	-2.488630
H	0.180902	-6.966763	-4.550530

EICO 9

0 1

C	-1.685197	-1.171494	1.436978
C	-2.299329	-0.808927	2.411639
H	-1.151367	-1.499412	0.569396

H	-2.860764	-0.450701	3.247184
C	-1.022982	3.827324	-3.834058
C	-0.328245	4.221342	-2.928962
H	-1.655187	3.483731	-4.624985
H	0.294902	4.561378	-2.128898
C	-3.341346	-4.463765	0.970934
C	-3.353816	-5.563900	0.473641
H	-3.324735	-3.484942	1.405297
H	-3.376469	-6.535810	0.032964
C	-2.771750	4.065434	0.172893
C	-1.820275	3.541809	0.700176
H	-3.605362	4.539394	-0.296198
H	-0.973568	3.082368	1.168659
C	1.371170	1.078013	-2.248633
C	2.219407	0.437117	-1.674572
H	0.620942	1.662507	-2.739479
H	2.961124	-0.135898	-1.159922
C	0.030422	-2.523958	-1.769163
C	-0.602257	-3.447726	-1.316005
H	0.587530	-1.694062	-2.152539
H	-1.191520	-4.236558	-0.899134
C	1.267374	2.498812	2.580824
C	0.646252	1.465086	2.639789
H	1.814169	3.411647	2.491588
H	0.072509	0.562382	2.678169
C	2.384435	4.137297	-0.322427
C	2.183616	5.284805	-0.006327
H	2.551369	3.119142	-0.606962
H	2.020768	6.302127	0.271531
C	3.110672	-3.015167	0.007024
C	4.027762	-2.400706	0.495559
H	2.279326	-3.531226	-0.422843
H	4.813246	-1.820470	0.927800
C	-2.515925	-0.268856	-2.357106
C	-2.454861	0.833262	-1.868311
H	-2.548233	-1.259161	-2.753346
H	-2.393722	1.804723	-1.423609
C	4.421996	-0.573150	-4.182785
C	4.963894	-1.643792	-4.054782
H	3.919219	0.363408	-4.277126
H	5.425213	-2.600259	-3.923205
C	5.375905	1.080013	0.552197
C	4.422290	1.093405	1.292773
H	6.215635	1.064946	-0.112016
H	3.567269	1.118110	1.934509
C	-4.825955	1.702443	4.204222
C	-4.255692	2.120334	3.225244
H	-5.341941	1.332893	5.066254
H	-3.746187	2.508756	2.368044
C	-3.816623	2.338971	-6.317238
C	-3.581185	1.732002	-5.300743
H	-4.027965	2.863155	-7.222564
H	-3.360069	1.207245	-4.392703
C	6.312384	-5.172148	-3.069551
C	5.707334	-4.627351	-2.179247
H	6.852486	-5.666315	-3.845568
H	5.164638	-4.138033	-1.396645
C	7.589551	0.410918	-2.336805
C	8.496223	0.896201	-1.705587
H	6.785367	-0.019605	-2.898404
H	9.308851	1.320240	-1.159328
C	-6.919986	0.256588	7.195300
C	-6.863235	-0.496127	6.254184

H	-6.981298	0.910464	8.036116
H	-6.799417	-1.147369	5.406850
C	-6.569883	-6.461166	4.171327
C	-5.693014	-6.227153	3.376738
H	-7.340124	-6.681880	4.875870
H	-4.920967	-6.006649	2.669485
C	-6.172312	-2.657427	3.134273
C	-5.928812	-1.503969	2.873263
H	-6.377041	-3.685289	3.350982
H	-5.708131	-0.478498	2.665347
C	-8.204458	2.660091	-3.735732
C	-7.139367	2.520961	-4.283044
H	-9.146822	2.780481	-3.250851
H	-6.193463	2.396324	-4.766058

EICO 10

0 1

C	-2.688822	3.442184	-0.069161
C	-2.117912	3.494294	-1.122096
H	-3.215808	3.385817	0.860213
H	-1.621555	3.513626	-2.068926
C	1.760903	0.062423	-1.278301
C	1.715385	1.118801	-0.715526
H	1.802469	-0.883313	-1.769403
H	1.675118	2.052452	-0.195894
C	-2.201944	-1.170482	-2.105693
C	-1.917583	-0.231977	-1.415748
H	-2.474346	-2.017521	-2.697763
H	-1.683871	0.601011	-0.786963
C	-5.198769	1.207517	0.035746
C	-4.666101	0.213909	0.441881
H	-5.673576	2.094834	-0.322934
H	-4.193315	-0.681232	0.787850
C	-7.027060	-6.217098	-0.301279
C	-5.859961	-6.059905	-0.082791
H	-8.068898	-6.339039	-0.499064
H	-4.819838	-5.903180	0.099659
C	-6.684062	4.784082	-0.301665
C	-5.809043	4.572770	-1.091923
H	-7.455171	4.977379	0.409129
H	-5.019926	4.360290	-1.779962
C	-8.274626	4.413341	-3.814060
C	-8.729699	4.139411	-4.886855
H	-7.860861	4.650046	-2.857312
H	-9.142734	3.901334	-5.840243
C	-5.514362	2.078112	-3.800953
C	-4.538718	1.567969	-3.326196
H	-6.386425	2.536537	-4.215343
H	-3.675135	1.101262	-2.900462
C	1.766482	4.094635	1.744120
C	0.700180	4.035796	1.201093
H	2.714264	4.153706	2.229375
H	-0.250484	3.974230	0.712381
C	-7.332551	-2.410194	-1.286178
C	-6.660509	-1.520179	-1.726020
H	-7.937745	-3.204879	-0.906886
H	-6.064613	-0.719403	-2.108082
C	-8.195966	-2.935096	2.587310
C	-7.859470	-4.076338	2.447016
H	-8.481175	-1.912296	2.708992
H	-7.540959	-5.085363	2.304019
C	-10.435995	-4.823342	-1.314683

C	-10.305447	-4.529875	-0.161179
H	-10.559076	-5.077288	-2.342995
H	-10.170154	-4.258221	0.862945
C	0.120514	-2.778190	0.180548
C	0.799355	-3.407529	0.940638
H	-0.497556	-2.208061	-0.482579
H	1.404447	-3.968328	1.615873
C	-4.045788	-4.188518	-2.398789
C	-3.109806	-4.774550	-2.862653
H	-4.882188	-3.665151	-1.985012
H	-2.281743	-5.304145	-3.274989
C	-8.613105	1.009551	2.402042
C	-8.158618	0.447022	1.446695
H	-9.021917	1.511834	3.249032
H	-7.743533	-0.057504	0.599487
C	-5.008831	2.694524	3.087839
C	-4.131872	3.332287	3.598485
H	-5.778880	2.111628	2.629944
H	-3.340416	3.877414	4.065428
C	-4.462290	-3.167858	1.381691
C	-3.264569	-3.200811	1.361049
H	-5.530180	-3.129906	1.403784
H	-2.196478	-3.228349	1.346808
C	-1.677741	0.815627	2.333437
C	-0.661192	0.330387	1.924504
H	-2.584659	1.244409	2.703466
H	0.237795	-0.110632	1.553731
C	-0.380321	1.915673	-3.891111
C	-1.254064	2.350361	-4.585394
H	0.389240	1.521141	-3.263066
H	-2.042740	2.722672	-5.199274
C	-1.109228	3.582968	5.786547
C	-0.592384	3.533051	4.707611
H	-1.563542	3.616992	6.750073
H	-0.124576	3.485024	3.747728

EICO 11

0 1

C	-2.482010	3.105471	-3.085835
C	-1.951460	4.061924	-2.595204
H	-2.951891	2.242176	-3.507943
H	-1.480119	4.906641	-2.145318
C	0.992884	4.013179	-0.196971
C	0.141702	4.394227	0.554996
H	1.749548	3.668882	-0.865162
H	-0.625511	4.729481	1.219715
C	-1.977108	-2.755262	-2.185874
C	-1.338992	-2.076154	-1.432575
H	-2.554903	-3.350812	-2.856700
H	-0.792311	-1.449197	-0.761526
C	-4.891892	1.380413	-1.376041
C	-4.243088	0.372396	-1.378706
H	-5.445145	2.295160	-1.375439
H	-3.653333	-0.519258	-1.401685
C	-7.131455	-1.783999	0.083384
C	-7.595325	-0.679250	0.094848
H	-6.700216	-2.760869	0.062443
H	-7.984560	0.316004	0.100657
C	-8.492306	2.542221	-2.089373
C	-8.980382	1.507217	-2.444477
H	-8.041533	3.460044	-1.777164
H	-9.403459	0.579513	-2.757772

C	-6.108051	2.488726	-4.891521
C	-5.158111	2.890897	-5.500435
H	-6.953182	2.132036	-4.342275
H	-4.306803	3.239178	-6.040336
C	-3.376309	-0.171688	-4.837058
C	-2.211076	-0.268833	-4.576065
H	-4.417925	-0.075858	-5.051921
H	-1.178378	-0.356850	-4.320369
C	-3.524875	3.992494	1.849960
C	-3.222592	3.478401	0.809814
H	-3.806356	4.435528	2.780440
H	-2.958080	3.016982	-0.117302
C	-7.183823	-1.321791	-4.658283
C	-6.803698	-0.886036	-3.608347
H	-7.522930	-1.704052	-5.594269
H	-6.456551	-0.493361	-2.674032
C	-1.535511	2.157632	4.370634
C	-1.613259	2.397946	5.542019
H	-1.483446	1.930382	3.325507
H	-1.679187	2.604360	6.585898
C	0.463335	1.184658	-2.375145
C	1.322556	0.349795	-2.381201
H	-0.307034	1.928324	-2.376340
H	2.084143	-0.396126	-2.389525
C	-5.532189	-4.021886	-2.311837
C	-5.047862	-4.813252	-1.554436
H	-5.970483	-3.313161	-2.981920
H	-4.619302	-5.514616	-0.874882
C	-3.643735	-3.039301	1.173109
C	-4.154188	-2.969061	2.254365
H	-3.178233	-3.089185	0.211968
H	-4.605358	-2.889558	3.217066
C	-0.851101	0.800463	0.941896
C	-1.778810	0.155745	1.343334
H	-0.039076	1.393880	0.582569
H	-2.612083	-0.409320	1.705205
C	-5.681471	4.899496	-2.217168
C	-5.869593	5.109708	-1.052778
H	-5.504506	4.692212	-3.250225
H	-6.023637	5.285237	-0.011903
C	-1.629964	6.882570	3.589842
C	-1.403293	5.781810	4.003125
H	-1.826378	7.862812	3.220072
H	-1.213857	4.792648	4.362652
C	-8.060634	3.038658	1.417971
C	-6.886238	2.799536	1.411968
H	-9.106221	3.247604	1.418133
H	-5.835677	2.593576	1.414305
C	-5.657335	4.003070	4.740110
C	-4.879393	4.771783	5.227905
H	-6.342706	3.317342	4.291936
H	-4.184364	5.462232	5.649437
C	-5.315862	0.145520	2.847955
C	-4.665386	0.696425	3.690541
H	-5.889342	-0.339093	2.085333
H	-4.076630	1.198017	4.427298

EICO 12

0 1

C	1.299849	1.292583	2.022093
C	1.837207	0.527938	2.773252
H	0.815646	1.973042	1.352924

H	2.326806	-0.150717	3.437732
C	-2.239190	0.046250	0.114861
C	-1.552836	-0.162131	1.074485
H	-2.867830	0.213506	-0.730758
H	-0.930528	-0.349938	1.922959
C	-5.424034	-1.723784	-0.821078
C	-5.741578	-0.680837	-0.325145
H	-5.132342	-2.655604	-1.251180
H	-6.008583	0.250224	0.124294
C	-4.682715	1.785350	2.091164
C	-5.031465	2.783154	1.527852
H	-4.373152	0.890236	2.586796
H	-5.336889	3.677156	1.033558
C	-3.082842	-4.300897	0.286871
C	-2.208006	-3.567515	-0.079715
H	-3.880331	-4.931666	0.614958
H	-1.417905	-2.915481	-0.389491
C	4.590119	-1.083508	-0.311933
C	4.871669	0.037339	0.006315
H	4.327136	-2.084434	-0.577177
H	5.105267	1.043323	0.282779
C	-1.381512	3.636310	0.858659
C	-0.508189	4.384060	0.520398
H	-2.158518	2.967580	1.161518
H	0.274083	5.043643	0.211630
C	1.216972	-1.934110	-0.383764
C	1.177481	-2.797510	-1.214662
H	1.258415	-1.154481	0.346721
H	1.127359	-3.562328	-1.960720
C	0.805221	1.900640	-1.703301
C	1.832201	1.287799	-1.632624
H	-0.109716	2.447828	-1.743893
H	2.736045	0.722913	-1.554013
C	3.566417	-4.484907	0.895417
C	4.746957	-4.653303	0.773998
H	2.511215	-4.344995	0.988663
H	5.795596	-4.820307	0.655764
C	0.239955	-6.226543	0.516004
C	1.094215	-7.048265	0.341836
H	-0.524406	-5.494762	0.669248
H	1.860394	-7.773982	0.186325
C	0.307469	-5.747289	-3.171631
C	0.283067	-5.054676	-4.148078
H	0.335578	-6.351588	-2.290565
H	0.261313	-4.442885	-5.020691
C	3.705764	-6.770160	-1.983057
C	3.763888	-7.648779	-2.794930
H	3.660626	-5.990304	-1.251747
H	3.805581	-8.423873	-3.525692
C	-0.463803	-3.055506	3.029803
C	0.728406	-2.987667	3.128849
H	-1.526878	-3.114391	2.942060
H	1.791394	-2.928232	3.227067
C	-4.359610	-1.837849	3.878245
C	-4.092094	-1.835524	2.710140
H	-4.596341	-1.843121	4.917715
H	-3.847963	-1.829019	1.668957
C	4.172861	3.242196	1.490282
C	4.763347	3.919341	0.698455
H	3.627571	2.635546	2.180370
H	5.272021	4.537861	-0.005663
C	-6.660889	-5.538990	1.096413
C	-6.449820	-4.438514	1.518030

H	-6.859735	-6.517885	0.724458
H	-6.255773	-3.457876	1.894045
C	2.385738	5.166660	-1.535514
C	2.387935	6.360029	-1.435338
H	2.372538	4.100152	-1.615874
H	2.394171	7.422487	-1.349705
C	7.240623	-6.359571	-1.211089
C	8.200899	-5.895103	-0.667582
H	6.380635	-6.770815	-1.694586
H	9.062612	-5.486910	-0.191093
C	4.401643	-1.837961	3.330117
C	4.158620	-2.174258	4.453907
H	4.614960	-1.533543	2.326680
H	3.953401	-2.482434	5.453753

EICO 13

0 1

C	5.984653	0.842480	-0.860939
C	6.324670	1.549598	0.044664
H	5.683565	0.222051	-1.678376
H	6.633122	2.180302	0.847283
C	2.600829	4.204047	0.873791
C	3.175023	3.338357	1.470686
H	2.079002	4.958908	0.325746
H	3.657960	2.553412	2.008274
C	0.098198	5.312589	-1.602875
C	1.117651	5.666385	-2.123466
H	-0.815133	4.979781	-1.157880
H	2.017853	5.966165	-2.614341
C	3.369687	2.570025	-2.362872
C	3.549164	2.204483	-3.490645
H	3.204066	2.875305	-1.351395
H	3.698813	1.861912	-4.492351
C	-3.189394	3.328160	-1.185132
C	-2.570054	3.059735	-0.194542
H	-3.735070	3.550193	-2.075614
H	-2.013783	2.805742	0.684194
C	-0.177305	2.080532	2.408844
C	-1.131964	1.762517	3.060342
H	0.672148	2.368302	1.826251
H	-1.985439	1.493875	3.644347
C	3.044915	-1.169118	-1.955479
C	3.431707	-2.218975	-1.525648
H	2.697527	-0.225980	-2.321801
H	3.755983	-3.152510	-1.122940
C	3.248704	-3.893765	1.623531
C	3.080567	-5.021596	1.257815
H	3.380583	-2.881217	1.940970
H	2.932717	-6.028857	0.941635
C	0.314766	1.606088	-1.550500
C	0.467647	0.744275	-0.730731
H	0.187480	2.368060	-2.289501
H	0.597700	-0.031583	-0.006526
C	-1.112125	-1.808426	1.295911
C	-0.139568	-2.489046	1.132945
H	-1.984480	-1.205726	1.431754
H	0.725355	-3.097771	0.983960
C	2.399739	-0.301178	2.380954
C	3.315365	-0.205980	1.613369
H	1.562334	-0.378468	3.038841
H	4.122747	-0.110381	0.918828
C	5.637934	-0.786188	-4.335607

C	6.412213	0.108474	-4.522205
H	4.936065	-1.569006	-4.151024
H	7.089964	0.918554	-4.677176
C	-3.801251	-1.064352	-1.929634
C	-2.747381	-0.500264	-1.847190
H	-4.734331	-1.576342	-1.992154
H	-1.803971	-0.000782	-1.757813
C	3.826362	5.460717	-4.798069
C	2.946827	6.098913	-5.302254
H	4.612192	4.887963	-4.353059
H	2.162794	6.663375	-5.753701
C	-4.271657	1.830864	-4.254520
C	-4.546084	2.773772	-4.940502
H	-4.022365	0.993496	-3.635698
H	-4.795203	3.608177	-5.555735
C	3.299325	0.386872	-6.797732
C	2.231394	0.865953	-6.544436
H	4.252934	-0.040651	-7.009310
H	1.285813	1.302764	-6.305822
C	-4.295512	0.328604	1.389514
C	-4.467337	-0.278352	2.407858
H	-4.133538	0.859063	0.475956
H	-4.621203	-0.811154	3.318692
C	6.862597	3.481318	-3.377769
C	7.296416	4.049223	-4.339201
H	6.471193	2.967945	-2.525234
H	7.683268	4.561377	-5.190548
C	-4.378016	1.975747	4.948610
C	-3.772853	1.699205	5.943560
H	-4.909779	2.213387	4.054870
H	-3.237506	1.461489	6.834162
C	-0.755182	2.949693	-4.977567
C	0.339708	3.419141	-4.847027
H	-1.739981	2.548586	-5.082031
H	1.315475	3.836968	-4.719778

EICO 14

0 1

C	1.405885	2.545698	0.461805
C	1.996962	2.197394	1.444538
H	0.900581	2.855187	-0.427631
H	2.531795	1.889353	2.316813
C	2.710971	1.244359	-2.603565
C	2.152775	0.191384	-2.730619
H	3.211501	2.182854	-2.473329
H	1.657120	-0.747009	-2.832189
C	0.935278	5.133816	3.176774
C	1.010671	6.006191	3.994021
H	0.881593	4.353886	2.446086
H	1.072216	6.790867	4.713073
C	5.164275	1.153544	0.340199
C	5.664868	1.805624	1.212167
H	4.712306	0.601522	-0.454596
H	6.106970	2.393924	1.987257
C	2.901248	2.621228	5.000135
C	3.955731	2.082627	4.817145
H	1.964555	3.108860	5.155162
H	4.894218	1.594742	4.662488
C	3.808733	0.763324	-6.093077
C	4.678172	0.715048	-6.915514
H	3.052575	0.805154	-5.339224
H	5.464326	0.684286	-7.635673

C	6.688489	1.983679	-2.709492
C	6.252578	1.216436	-3.520551
H	7.058501	2.663667	-1.972182
H	5.852094	0.548222	-4.250163
C	2.463855	7.283521	0.292973
C	2.115537	7.988016	-0.612022
H	2.782257	6.669418	1.108127
H	1.816338	8.609644	-1.426941
C	4.167764	4.237653	-1.075365
C	4.339448	4.534494	-2.224118
H	4.017287	3.950800	-0.057473
H	4.494576	4.787257	-3.251642
C	5.285949	5.588299	1.967336
C	4.534406	5.182964	2.808577
H	5.965072	5.918586	1.211998
H	3.866082	4.811978	3.554345
C	7.821253	2.463471	-6.535185
C	7.815696	2.713450	-7.706314
H	7.821055	2.256204	-5.486119
H	7.820511	2.946328	-8.746369
C	7.770455	4.958380	-0.618017
C	8.226387	4.318370	0.286874
H	7.355183	5.521824	-1.426332
H	8.619071	3.751707	1.102042
C	0.304186	4.036703	-2.965376
C	0.791808	4.859789	-2.244153
H	-0.123370	3.296867	-3.603016
H	1.240979	5.589849	-1.603388
C	5.439391	7.645449	-2.188766
C	6.444921	7.876058	-2.799369
H	4.541965	7.432127	-1.645783
H	7.344801	8.054810	-3.343769
C	2.447574	8.582394	-4.279706
C	1.940882	7.496998	-4.302287
H	2.894336	9.551146	-4.236274
H	1.494640	6.526383	-4.315183
C	6.485493	-0.960624	4.747929
C	6.254082	-0.704315	3.601235
H	6.685367	-1.197912	5.767851
H	6.041077	-0.460345	2.582260
C	4.082701	10.979045	-2.158060
C	3.191097	11.768776	-2.280274
H	4.871912	10.265943	-2.054532
H	2.401374	12.477631	-2.380814
C	8.066584	5.210783	-4.436683
C	9.070349	5.199829	-3.783288
H	7.169146	5.209697	-5.018267
H	9.958714	5.179915	-3.194278
C	7.579957	3.544004	3.949071
C	8.022183	2.430973	3.974213
H	7.167522	4.528189	3.916095
H	8.396735	1.432370	4.003016
C	4.540487	5.621727	-5.703394
C	4.727380	4.461433	-5.939688
H	4.352184	6.648810	-5.477553
H	4.890138	3.425913	-6.151425

EICO 15

0 1

C	-0.428681	-0.946221	2.362900
C	0.528777	-0.437924	1.852612
H	-1.275528	-1.403476	2.821780

H	1.380359	0.010373	1.385283
C	3.078901	1.040716	-4.408574
C	3.533288	0.479555	-3.452440
H	2.684752	1.554478	-5.256278
H	3.926086	0.001917	-2.580205
C	-0.060569	2.956706	3.210887
C	0.242771	4.101131	3.396290
H	-0.321994	1.937832	3.023717
H	0.523735	5.119612	3.559691
C	1.198041	1.857582	-0.954979
C	1.256479	2.711192	-0.114925
H	1.153578	1.082986	-1.690195
H	1.315647	3.458305	0.646960
C	3.695546	2.880922	2.993318
C	4.284286	3.314894	2.043201
H	3.167046	2.519655	3.847757
H	4.798954	3.706939	1.191827
C	4.944281	7.369280	-2.808346
C	5.764984	6.592689	-3.206157
H	4.213329	8.056989	-2.447600
H	6.502515	5.900195	-3.549879
C	6.540514	5.987514	0.404538
C	7.182144	5.800420	1.399222
H	5.960268	6.155686	-0.478216
H	7.733729	5.644846	2.298838
C	1.656790	7.579234	3.785935
C	2.461371	6.961446	4.423825
H	0.932767	8.113542	3.211482
H	3.175801	6.409842	4.995620
C	1.595003	4.299465	-3.575791
C	2.571137	4.777401	-4.080027
H	0.736881	3.868215	-3.110257
H	3.448241	5.200853	-4.518014
C	2.806855	5.885873	-0.489787
C	2.999205	6.196553	0.652315
H	2.645729	5.596197	-1.507479
H	3.164869	6.466969	1.673467
C	6.326099	6.190300	5.368860
C	5.833065	5.364760	4.654391
H	6.764541	6.930085	5.998851
H	5.383364	4.633948	4.013690
C	0.259794	-1.585308	-1.575664
C	0.764813	-1.576833	-2.661595
H	-0.179707	-1.577696	-0.602109
H	1.221157	-1.553714	-3.625490
C	4.685350	2.938870	-1.448828
C	5.876819	3.031218	-1.351757
H	3.621675	2.849668	-1.522762
H	6.937355	3.131368	-1.280992
C	4.988327	0.486051	-7.425056
C	4.625457	-0.119689	-8.392211
H	5.309956	1.033553	-6.565255
H	4.300049	-0.667508	-9.246782
C	6.151904	3.240680	-9.591363
C	6.565469	4.313809	-9.924304
H	5.781300	2.285441	-9.285503
H	6.930780	5.266539	-10.232531
C	6.116741	3.724751	-6.045575
C	5.812945	3.336099	-4.952566
H	6.373258	4.055273	-7.029191
H	5.533558	2.998977	-3.975648
C	2.245370	3.747866	6.313222
C	3.385056	3.856305	6.664220

H	1.234927	3.649426	5.983313
H	4.401537	3.962546	6.968662
C	-1.542146	7.224207	1.596162
C	-0.527135	6.943702	1.026402
H	-2.451887	7.464856	2.096651
H	0.384179	6.690033	0.527959
C	8.860725	4.295449	-3.198489
C	8.872050	4.953728	-2.197949
H	8.838787	3.714023	-4.092789
H	8.863627	5.537040	-1.303316
C	3.568499	-1.164557	-0.119292
C	4.047115	-0.147843	0.295895
H	3.125126	-2.060323	-0.492073
H	4.461660	0.763051	0.669419

EICO 16

0 1

C	0.367803	2.535481	-0.458241
C	1.466129	2.358094	-0.011607
H	-0.615611	2.692358	-0.845104
H	2.444853	2.197920	0.387490
C	1.429784	1.221463	-3.508862
C	1.281749	0.040105	-3.641423
H	1.575296	2.270976	-3.367356
H	1.155004	-1.013878	-3.742028
C	0.277541	3.396462	3.161908
C	0.809189	4.111324	3.963766
H	-0.183634	2.756726	2.439822
H	1.281080	4.768001	4.660690
C	4.364994	0.963115	-1.372130
C	5.202747	1.563902	-0.761129
H	3.594859	0.437663	-1.896169
H	5.929717	2.109638	-0.202870
C	-3.186518	1.514204	-1.869883
C	-2.217790	1.165187	-2.482734
H	-4.047488	1.822205	-1.317911
H	-1.342312	0.870284	-3.021158
C	-3.585886	2.363650	4.475217
C	-2.927416	3.363352	4.424070
H	-4.181575	1.479587	4.501323
H	-2.338093	4.252151	4.353094
C	0.846054	7.189348	0.341240
C	0.879449	5.999652	0.483249
H	0.821727	8.249879	0.232982
H	0.911856	4.936653	0.608337
C	-2.363859	4.703447	0.594879
C	-2.665066	3.620064	1.009372
H	-2.075663	5.668616	0.242505
H	-2.932818	2.660767	1.398592
C	-1.259710	0.009702	2.247816
C	-1.571596	0.108085	1.094700
H	-0.966424	-0.071364	3.273320
H	-1.844990	0.206316	0.065819
C	2.115855	7.645620	4.514309
C	2.584887	7.041293	3.591929
H	1.689883	8.177766	5.336834
H	2.998397	6.490735	2.774394
C	-6.621529	5.510686	2.930357
C	-5.525107	5.258902	2.519182
H	-7.596155	5.736376	3.298060
H	-4.547668	5.027733	2.149873
C	4.105216	4.559174	0.921997

C	4.211449	4.007973	1.980410
H	3.991130	5.025059	-0.032728
H	4.296940	3.498660	2.914003
C	2.687747	0.815773	3.117979
C	3.362059	0.025381	2.520303
H	2.073920	1.515286	3.643647
H	3.950228	-0.687810	1.984434
C	2.396937	-2.214378	-0.638980
C	1.693345	-1.311089	-0.286718
H	3.034168	-3.017744	-0.930434
H	1.076106	-0.502536	0.042445
C	2.611534	4.999159	-2.523293
C	3.515712	4.259291	-2.786879
H	1.811250	5.656343	-2.264082
H	4.314805	3.586332	-3.003909
C	5.781918	-1.947477	0.402031
C	5.867405	-2.889562	1.136043
H	5.691867	-1.097823	-0.240465
H	5.948485	-3.727593	1.789632
C	-2.636653	7.017728	3.696874
C	-1.490631	6.876856	3.374853
H	-3.663458	7.127801	3.967200
H	-0.469627	6.750365	3.081472
C	-0.392026	0.840126	5.679283
C	0.425895	-0.034588	5.656226
H	-1.124426	1.618635	5.678110
H	1.164624	-0.803117	5.626916
C	-0.655046	8.274245	6.703897
C	-0.037797	8.669394	7.650644
H	-1.198455	7.920268	5.853635
H	0.503618	9.019501	8.499365
C	-5.637094	1.715000	1.562554
C	-5.100011	0.660432	1.377217
H	-6.103678	2.659345	1.742980
H	-4.612070	-0.276001	1.225677

EICO 17

0 1

C	1.132982	-2.989334	-1.535512
C	0.700581	-1.871817	-1.488460
H	1.529021	-3.983098	-1.568267
H	0.329960	-0.869496	-1.438097
C	-2.414684	-3.810391	-1.615345
C	-3.519930	-4.225029	-1.406263
H	-1.427592	-3.432309	-1.785002
H	-4.512425	-4.574169	-1.216682
C	1.076232	-2.823645	2.190416
C	1.998891	-3.136096	2.888271
H	0.251545	-2.535969	1.573593
H	2.823953	-3.405603	3.508464
C	-2.193828	-1.120700	0.771364
C	-1.439669	-0.309610	1.231028
H	-2.863705	-1.836085	0.346041
H	-0.757705	0.409777	1.633649
C	5.273515	-2.431723	-0.471360
C	4.234181	-2.611504	0.098906
H	6.187873	-2.253118	-0.995428
H	3.308193	-2.764094	0.613873
C	4.298894	1.681058	1.197479
C	4.698113	1.576607	0.072350
H	3.935638	1.751562	2.199486
H	5.042125	1.469695	-0.932545

C	3.539281	0.757703	-3.523274
C	3.533805	-0.231417	-2.846440
H	3.506129	1.642658	-4.119975
H	3.516654	-1.108768	-2.235539
C	-4.648837	1.258364	2.190440
C	-5.615511	1.928461	2.417567
H	-3.787301	0.658942	1.977834
H	-6.478284	2.518326	2.628006
C	-4.826251	-0.868425	-1.895323
C	-5.240080	-0.121075	-1.055820
H	-4.446194	-1.539785	-2.632428
H	-5.589784	0.548885	-0.300999
C	1.679047	5.553380	-0.637631
C	2.497734	4.821226	-0.159789
H	0.939822	6.187663	-1.074018
H	3.230295	4.160200	0.250323
C	7.082406	-0.824490	-3.158671
C	8.162925	-1.198220	-2.802890
H	6.114817	-0.491972	-3.469528
H	9.130207	-1.525458	-2.496879
C	-2.067901	3.762740	1.925535
C	-1.035536	3.999657	1.364770
H	-2.990292	3.535818	2.412926
H	-0.115805	4.198254	0.855185
C	-0.939723	-8.456710	-2.259695
C	-1.428449	-7.390000	-2.021404
H	-0.512201	-9.409071	-2.475070
H	-1.873238	-6.440231	-1.813115
C	-0.059444	1.863617	-1.147323
C	1.042272	1.607653	-0.750950
H	-1.040613	2.109725	-1.488326
H	2.024700	1.382328	-0.393043
C	1.961344	4.094494	-4.974574
C	1.762445	3.781107	-3.835480
H	2.139866	4.374790	-5.987511
H	1.578949	3.499951	-2.819323
C	-1.926127	5.115497	-1.812785
C	-1.330594	5.081515	-2.851287
H	-2.444561	5.133416	-0.880213
H	-0.787699	5.040559	-3.769287
C	4.922956	-0.997188	3.266335
C	4.645439	-0.773783	4.409701
H	5.161009	-1.188972	2.242152
H	4.400826	-0.568305	5.426773
C	-6.956535	-3.603403	-0.662469
C	-7.280135	-4.748986	-0.534349
H	-6.657586	-2.584042	-0.782272
H	-7.578289	-5.765582	-0.416236
C	2.188924	-6.512955	-1.261620
C	3.208212	-5.919611	-1.052457
H	1.275452	-7.036117	-1.448914
H	4.108755	-5.378229	-0.864392
C	1.004681	1.775244	3.240473
C	1.485894	0.678498	3.196539
H	0.552162	2.741431	3.272057
H	1.908319	-0.302840	3.154641

EICO 18

0 1

C	0.195913	-0.331904	-1.173616
C	-0.462030	0.565818	-1.620287
H	0.774024	-1.136553	-0.768844

H	-1.066835	1.355391	-2.015876
C	2.641131	2.431214	-0.824717
C	3.511612	3.149264	-0.421754
H	1.856534	1.793067	-1.176291
H	4.281418	3.793171	-0.060310
C	-3.612691	-0.518979	-0.138006
C	-4.592714	-1.060564	-0.565308
H	-2.739307	-0.036184	0.248356
H	-5.469717	-1.539643	-0.938695
C	-0.276880	4.697581	-0.698426
C	-1.140607	4.054286	-0.172266
H	0.494012	5.272445	-1.164144
H	-1.910775	3.480573	0.298147
C	-1.050378	-4.366581	-1.987805
C	-1.636541	-3.404956	-1.578947
H	-0.520960	-5.214883	-2.358512
H	-2.161689	-2.547033	-1.214533
C	5.317355	-0.538822	2.229866
C	4.764841	0.202449	1.469162
H	5.811627	-1.194625	2.909826
H	4.272575	0.868281	0.792911
C	1.351828	-3.187486	0.759141
C	2.327839	-2.538625	1.008422
H	0.470543	-3.748967	0.539095
H	3.197994	-1.960865	1.236246
C	-3.534195	2.052191	-2.640161
C	-2.945075	3.059356	-2.914412
H	-4.048972	1.154523	-2.377073
H	-2.429025	3.963368	-3.157746
C	-0.735197	1.251222	1.971236
C	-1.688434	1.097744	2.680584
H	0.106146	1.399669	1.331917
H	-2.537490	0.956602	3.312977
C	-5.354314	0.021521	3.998954
C	-4.724326	-0.770615	3.357825
H	-5.922897	0.719090	4.571324
H	-4.151280	-1.474255	2.790834
C	-7.389356	1.759556	-2.918352
C	-6.877709	1.951923	-1.852322
H	-7.842462	1.592269	-3.868857
H	-6.405585	2.134313	-0.909152
C	2.220644	6.740778	-3.167907
C	2.547020	5.612345	-2.931554
H	1.926020	7.742876	-3.397033
H	2.840718	4.611134	-2.703176
C	-5.738839	4.696808	-4.445162
C	-6.374067	5.568585	-4.965981
H	-5.175461	3.921092	-3.970324
H	-6.948178	6.340720	-5.424681
C	-4.990155	-5.979406	0.638496
C	-4.194216	-5.594267	-0.168880
H	-5.701565	-6.329451	1.351140
H	-3.474181	-5.244213	-0.877489
C	-2.365564	-3.409494	2.016312
C	-1.613241	-2.519565	2.295263
H	-3.033969	-4.202034	1.756362
H	-0.943183	-1.722565	2.532792
C	-2.289168	6.227967	-4.441052
C	-1.113378	6.009416	-4.363549
H	-3.343737	6.393669	-4.494209
H	-0.063878	5.822220	-4.289132
C	-4.839916	2.449410	1.251799
C	-4.745558	3.487509	0.659698

H	-4.930814	1.522144	1.775822
H	-4.661133	4.410633	0.124305
C	-3.701823	6.424018	-1.292817
C	-4.877533	6.509475	-1.508552
H	-2.654943	6.327867	-1.103607
H	-5.927093	6.571866	-1.701255
C	-8.802563	6.426072	-1.408013
C	-8.392592	5.392464	-1.852426
H	-9.176159	7.341117	-1.009152
H	-8.025081	4.469294	-2.246612
C	1.142305	10.055230	-4.789631
C	0.201890	9.314909	-4.768325
H	1.974197	10.720776	-4.816318
H	-0.631168	8.646172	-4.750207

EICO 19

0 1

C	1.057626	2.428330	1.247224
C	0.470027	1.433163	1.567563
H	1.593460	3.310563	0.961634
H	-0.059265	0.548554	1.854335
C	-2.663462	2.944178	2.221715
C	-3.801328	2.860429	1.855179
H	-1.644238	3.013381	2.534043
H	-4.813226	2.805503	1.517069
C	3.381319	-0.409062	0.333317
C	4.163812	-1.179901	-0.145977
H	2.681884	0.281937	0.754474
H	4.850730	-1.872581	-0.579118
C	-0.896179	6.875166	0.728128
C	-0.503560	7.502808	1.669105
H	-1.251713	6.313028	-0.108464
H	-0.160796	8.062734	2.509086
C	3.592454	4.979795	0.437686
C	2.659773	5.721403	0.561443
H	4.419228	4.312153	0.323692
H	1.819503	6.372515	0.670782
C	-2.357273	4.756281	-2.281166
C	-2.372363	4.140935	-1.253393
H	-2.335135	5.294728	-3.201345
H	-2.395026	3.600457	-0.330194
C	0.877813	-3.345997	0.529927
C	1.010153	-4.438358	1.006022
H	0.755059	-2.375182	0.096295
H	1.159052	-5.411856	1.421396
C	-1.640175	-1.699937	2.361773
C	-2.312075	-0.713969	2.253409
H	-1.045919	-2.581774	2.453787
H	-2.905440	0.169131	2.153286
C	6.808897	2.958763	-0.191000
C	5.968424	2.114450	-0.319583
H	7.561375	3.705584	-0.076083
H	5.220825	1.355835	-0.427362
C	5.045715	-5.070756	-1.508110
C	4.008523	-4.635535	-1.096206
H	5.970171	-5.454793	-1.875036
H	3.086234	-4.250585	-0.713971
C	-2.731955	-4.260796	-0.111372
C	-3.925993	-4.358780	-0.105896
H	-1.666340	-4.182366	-0.110110
H	-4.991199	-4.451009	-0.102348

C	3.188932	-7.284822	1.332561
C	2.514076	-7.863706	2.134133
H	3.788426	-6.765193	0.616832
H	1.921969	-8.387076	2.849432
C	-5.981670	3.787645	-0.906399
C	-6.794691	2.952271	-0.630338
H	-5.249410	4.523842	-1.154940
H	-7.515149	2.202174	-0.386847
C	-4.158492	0.415362	-1.080884
C	-3.888582	-0.712813	-0.777378
H	-4.417241	1.415100	-1.354721
H	-3.639470	-1.719706	-0.510219
C	-8.538107	-0.431296	0.755697
C	-7.399209	-0.455815	0.385168
H	-9.550636	-0.413301	1.089581
H	-6.382666	-0.470133	0.049428
C	3.368955	2.837581	-2.480506
C	2.416693	2.192679	-2.145464
H	4.216236	3.419163	-2.764591
H	1.566399	1.616141	-1.848558
C	-7.745428	-4.139110	-0.154868
C	-7.583768	-5.297697	-0.409264
H	-7.893969	-3.105021	0.070251
H	-7.445086	-6.329788	-0.637101
C	8.099166	0.174799	2.153073
C	8.740554	-0.528761	2.878018
H	7.528774	0.805364	1.506782
H	9.309729	-1.155326	3.524970
C	3.828762	-0.899910	-3.631028
C	3.858058	-2.089112	-3.771042
H	3.788550	0.159158	-3.490343
H	3.892115	-3.149309	-3.882569
C	0.609202	-0.843832	-1.971158
C	-0.388699	-0.422100	-1.457742
H	1.505132	-1.208020	-2.429090
H	-1.291010	-0.056122	-1.016745

EICO 20

0 1

C	1.098640	2.511734	2.550715
C	2.179571	2.006783	2.435438
H	0.135108	2.964200	2.648552
H	3.147694	1.572162	2.324151
C	2.548318	5.244999	-5.304312
C	2.894043	4.097294	-5.345083
H	2.245072	6.270057	-5.260332
H	3.180194	3.067938	-5.369135
C	0.913457	5.462562	4.359546
C	0.421148	5.081170	5.383728
H	1.344825	5.791259	3.437404
H	-0.024710	4.739695	6.292260
C	5.468184	3.135217	0.660509
C	5.415177	3.291812	1.847602
H	5.516236	3.002280	-0.399008
H	5.349963	3.427778	2.906218
C	-2.929805	2.702936	3.473051
C	-2.074986	2.342996	4.232345
H	-3.682670	3.013349	2.779931
H	-1.309265	2.018411	4.905837
C	4.408497	1.978836	-2.706553
C	5.514331	1.758594	-3.111946
H	3.421654	2.190740	-2.347515

H	6.499215	1.564685	-3.471614
C	-1.287206	5.461950	1.780305
C	-0.791741	5.495876	0.689439
H	-1.705731	5.421265	2.761574
H	-0.335722	5.530121	-0.277182
C	1.219886	3.533123	-2.150821
C	1.307407	3.131915	-1.024555
H	1.153965	3.900655	-3.151682
H	1.375086	2.781439	-0.015977
C	0.382057	7.268175	-2.698129
C	1.369465	6.896960	-2.129118
H	-0.488406	7.595588	-3.221329
H	2.241278	6.549310	-1.616731
C	2.395597	6.967673	1.271070
C	2.948191	5.904999	1.240020
H	1.886198	7.904650	1.289662
H	3.447797	4.959775	1.203845
C	-1.227559	3.336768	-7.635046
C	-0.110884	3.667494	-7.355762
H	-2.220362	3.042833	-7.889179
H	0.880671	3.967252	-7.089819
C	1.220833	0.913713	-5.383875
C	2.152548	0.386787	-4.847282
H	0.391703	1.384374	-5.864925
H	2.982496	-0.076544	-4.363735
C	-5.515176	3.770490	0.724352
C	-4.439587	4.267660	0.552294
H	-6.475723	3.330790	0.865938
H	-3.474541	4.706926	0.414117
C	3.879525	3.422441	5.285843
C	4.842882	2.745471	5.506554
H	3.008657	4.017519	5.103137
H	5.699026	2.144784	5.713044
C	-2.589852	4.075032	7.303515
C	-2.185283	4.447868	8.367033
H	-2.941699	3.745071	6.349637
H	-1.833947	4.786345	9.314942
C	0.124641	1.611484	7.115988
C	0.934628	1.563732	6.234925
H	-0.601307	1.666846	7.895426
H	1.653847	1.530196	5.446037
C	5.137533	6.070811	-1.389323
C	4.793757	5.509893	-2.390891
H	5.435477	6.555353	-0.486821
H	4.477849	5.014711	-3.284110
C	0.873825	10.690329	-3.617625
C	0.067787	11.485555	-4.007455
H	1.589414	9.969973	-3.285467
H	-0.648166	12.199717	-4.344878
C	-0.203645	8.241991	-6.063412
C	-0.508238	7.170323	-6.503427
H	0.055484	9.194782	-5.654930
H	-0.780477	6.214893	-6.895500
C	3.949992	8.426002	-4.374475
C	3.494945	9.034470	-5.299907
H	4.357176	7.873301	-3.555308
H	3.091808	9.578506	-6.123517

Section 2: Optimized cationic structures

DEC1
1 2

C	3.469658	2.231326	0.067951
H	3.175997	2.785834	0.931997
C	4.829678	-2.073870	-1.172262
H	4.391825	-2.783644	-1.838168
C	5.353504	-1.302055	-0.420684
H	5.844468	-0.619982	0.237292
C	3.833014	1.649085	-0.915764
H	4.188037	1.131607	-1.780325
C	0.593868	3.492377	-1.998175
H	1.654621	3.512307	-2.115629
C	-0.597736	3.499920	-1.869441
H	-1.660614	3.536394	-1.751023
C	-0.038805	0.314207	-0.681928
H	-0.029980	1.154084	-1.364791
C	-0.329115	-0.719510	-0.044971
H	-0.822134	-1.616951	0.305687
C	1.402850	-3.766132	-0.145456
H	0.570068	-3.982080	-0.777195
C	2.341677	-3.574303	0.573744
H	3.197636	-3.437074	1.196868
C	-2.376430	-3.289947	1.285614
H	-3.291087	-3.059775	0.784224
C	0.771762	0.181347	0.731414
H	0.420412	0.773106	1.569889
C	2.042707	-0.209569	0.577305
H	2.642330	-0.800422	-0.108721
C	-1.358329	-3.567978	1.853350
H	-0.460675	-3.851549	2.356358
C	-3.720386	-0.557176	-0.482009
H	-3.696108	-0.182428	0.519042
C	-3.750345	-0.980342	-1.602522
H	-3.802733	-1.359515	-2.598894
C	-4.102862	3.024456	-0.634695
H	-4.395527	2.113722	-1.109675
C	-3.797787	4.054587	-0.104906
H	-3.552699	4.984660	0.357943
C	-2.327582	1.316276	2.237148
H	-2.549066	2.203008	1.681305
C	-2.119075	0.315696	2.863019
H	-1.957179	-0.570480	3.436418

DEC 2
1 2

C	-0.029403	-1.263902	2.689157
C	-1.030638	-1.920709	2.634019
H	0.871082	-0.689656	2.761344
H	-1.927463	-2.500195	2.599375
C	-0.753949	1.784953	-3.017195
C	-0.558594	3.064041	-2.724907
H	-1.039119	1.466657	-4.017569
H	-0.276911	3.651450	-1.856819
C	-4.354501	-2.014591	0.894508
C	-4.071221	-3.124851	0.544895
H	-4.624967	-1.030949	1.209170
H	-3.849575	-4.123680	0.240499
C	-3.177351	1.657369	0.264163
C	-2.469780	1.055000	1.021544
H	-3.826773	2.199690	-0.387118
H	-1.853373	0.505242	1.703567
C	2.772462	0.714454	-2.253685
C	2.723114	0.591720	-1.062265

H	2.847720	0.825818	-3.312565
H	2.710373	0.489175	0.003933
C	-0.020989	-1.522703	-1.193759
C	-1.241923	-1.403644	-0.922702
H	0.997472	-1.872459	-1.122950
H	-2.197066	-1.576730	-0.449231
C	2.858532	1.123301	2.664397
C	3.424284	0.076977	2.520687
H	2.370085	2.061894	2.805996
H	3.934189	-0.853614	2.401376
C	1.274748	3.405827	0.331876
C	0.199205	3.443090	0.859600
H	2.238815	3.381195	-0.127358
H	-0.754992	3.489820	1.336911
C	2.473924	-3.025845	0.731556
C	3.308409	-2.743766	-0.080637
H	1.737249	-3.283742	1.460955
H	4.064608	-2.505607	-0.795477
C	-0.788439	-0.557747	-2.177131
C	-0.594810	0.806376	-1.984652
H	-1.053183	-0.920056	-3.166109
H	-0.313895	1.157725	-0.994212

DEC 3
1 2

C	4.237570	1.174320	-0.420402
C	4.425134	1.094083	-1.601125
H	4.082911	1.245467	0.634444
H	4.627463	1.026306	-2.647094
C	0.989522	4.677113	-0.425756
C	1.950796	4.043793	-0.757531
H	0.155325	5.278431	-0.139708
H	2.823868	3.500297	-1.048628
C	-0.334463	-2.380621	3.164954
C	0.556023	-3.147814	3.396548
H	-1.134464	-1.697261	2.979972
H	1.335169	-3.840930	3.625299
C	1.517297	-1.298726	-0.398574
C	1.342650	-2.500671	0.143067
H	2.506675	-0.892934	-0.600208
H	0.520834	-3.141654	0.441062
C	-0.818931	1.090438	-2.253997
C	-0.792397	1.687842	-1.147526
H	-1.149709	0.822186	-3.241242
H	-1.082433	2.374947	-0.374226
C	-3.983451	-0.913623	-2.075106
C	-4.367535	0.208095	-2.244033
H	-3.666100	-1.921198	-1.919164
H	-4.735245	1.197075	-2.404089
C	-1.751509	-3.800910	-1.040380
C	-2.192300	-3.505798	0.034031
H	-1.375108	-4.093883	-1.995710
H	-2.599635	-3.277986	0.994575
C	0.419188	0.722478	-1.349048
C	0.365906	-0.527420	-0.724293
H	1.390856	1.151609	-1.575783
H	-0.614069	-0.942139	-0.491709
C	-1.893802	0.643371	1.955474
C	-2.739397	0.247806	1.203049
H	-1.143921	1.004195	2.625962
H	-3.501622	-0.074888	0.525502
C	1.684667	0.654188	2.551415
C	1.634290	1.729566	2.024314

H	1.720495	-0.297722	3.035930
H	1.602891	2.693459	1.561442

DEC 4

1 2

C	2.442078	1.278628	1.444467
C	1.383937	1.703004	1.073519
H	3.392914	0.920783	1.774666
H	0.444918	2.107686	0.755522
C	0.911168	-1.654101	0.374732
C	-0.181906	-1.445833	1.229242
H	1.908770	-1.595454	0.807691
H	0.022383	-1.238627	2.274178
C	-1.248737	1.005907	3.640656
C	-2.356047	0.662077	3.339171
H	-0.270825	1.324659	3.926783
H	-3.349880	0.366130	3.082554
C	-2.059937	0.058013	-2.703526
C	-0.924481	0.345035	-2.958033
H	-3.077647	-0.185200	-2.485802
H	0.084611	0.602030	-3.200906
C	4.903093	-1.762785	0.753125
C	4.897394	-0.883666	-0.061201
H	4.952179	-2.544920	1.478196
H	4.916408	-0.104730	-0.792481
C	-5.039244	-0.614357	0.064886
H	-5.411831	-1.613517	0.100892
C	-1.870151	2.905542	-0.484813
H	-1.755086	2.780765	-1.539263
C	-2.015735	3.057027	0.695426
H	-2.149573	3.201576	1.745116
C	-4.661165	0.521370	0.022101
H	-4.338271	1.538904	-0.018027
C	-1.485471	-0.818613	0.610612
H	-1.775843	0.180753	0.330372
C	-1.568281	-2.052172	0.846218
H	-2.028481	-3.019616	0.943856
C	0.839891	-1.937105	-1.016706
H	-0.119471	-1.975681	-1.525364
C	1.983046	-2.128149	-1.670866
H	3.043441	-2.139553	-1.442016
C	2.757878	1.024378	-2.339219
H	2.542998	1.479142	-1.395555
C	3.012778	0.523636	-3.397897
H	3.248835	0.101140	-4.349719

DEC 5

1 2

C	-4.021940	-0.970195	1.152496
C	-4.293097	-1.816470	0.349642
H	-3.811754	-0.219116	1.881347
H	-4.556352	-2.576438	-0.351603
C	-0.816358	-2.530899	-1.401734
C	-1.532637	-1.497862	-1.549794
H	-0.421170	-3.505385	-1.631161
H	-2.246604	-0.837342	-2.010008
C	-5.654678	2.816844	0.059827
C	-6.046440	1.686754	0.112964
H	-5.325557	3.830440	0.008864
H	-6.411790	0.685829	0.163108
C	-1.394115	2.244106	-1.542073

C -2.388606 1.901063 -0.967868
H -0.528627 2.579903 -2.067378
H -3.303277 1.631058 -0.480098
C 0.462006 -0.688658 -0.173384
C -0.697549 -1.507979 -0.247793
H 0.767539 -0.359633 0.818562
H -1.209898 -1.719769 0.685493
C 2.341892 0.524641 -0.957119
C 1.241180 -0.241614 -1.241656
H 2.555426 0.765662 0.082413
H 0.992280 -0.506203 -2.264308
C 2.881615 -3.323196 -0.165567
C 2.309034 -3.480834 0.874447
H 3.407467 -3.210974 -1.087435
H 1.815179 -3.649733 1.805591
C 3.259995 1.027057 -1.948053
C 4.342457 1.688676 -1.573607
H 3.058994 0.835784 -3.000526
H 4.812249 2.005633 -0.650767
C 4.364336 -0.326689 1.852983
C 4.553086 0.796124 2.225612
H 4.208310 -1.335158 1.535362
H 4.740191 1.785294 2.580773
C 0.249215 2.085519 1.875826
C 1.031082 1.655576 2.675478
H -0.455572 2.471862 1.171350
H 1.722483 1.286321 3.399795

DEC 6

1 2

C 2.709866 -2.454792 -0.875470
C 2.718327 -2.710983 -2.046013
H 2.710630 -2.235010 0.170211
H 2.751372 -2.954638 -3.084974
C 3.073844 2.122032 -2.249799
C 3.771909 1.219118 -1.884866
H 2.465036 2.932402 -2.587003
H 4.410259 0.418800 -1.580758
C 1.320538 -0.602913 2.169826
C 0.729021 -1.640956 2.068490
H 1.844148 0.322827 2.281456
H 0.221990 -2.578786 1.983303
C 0.385704 0.010593 -1.606245
C -0.575614 -0.935790 -1.161428
H 1.387555 -0.002085 -2.005112
H -0.473712 -1.996798 -0.933189
C -2.430916 2.068613 2.087094
C -1.765246 1.226108 2.619086
H -3.034354 2.824817 1.634831
H -1.171068 0.480252 3.100965
C -1.442947 -4.081307 0.337990
C -0.377512 -4.442695 -0.075854
H -2.398453 -3.776021 0.708114
H 0.566066 -4.785128 -0.441084
C 1.302281 3.065077 2.015294
C 2.169851 2.886477 1.207345
H 0.539162 3.235598 2.743022
H 2.948853 2.740765 0.489436
C -1.524433 0.008878 -0.686113
C -0.317395 0.764467 -0.624420
H -2.461363 -0.028717 -0.151723

H	0.067623	1.394338	0.174904
C	-4.499957	-1.902529	0.252055
C	-4.191304	-1.421279	1.304832
H	-4.807807	-2.337028	-0.673228
H	-3.947002	-0.984613	2.248041
C	-0.950130	3.311808	-2.198001
C	-0.368287	3.751271	-1.246553
H	-1.476503	2.960153	-3.057662
H	0.152331	4.156521	-0.405776

DEC 7

1 2

C	1.596727	-2.398815	1.827301
C	0.958491	-2.210825	2.823652
H	2.158984	-2.577258	0.933738
H	0.389989	-2.052381	3.711541
C	3.133619	-4.183433	-1.167796
C	2.532351	-3.274616	-1.667221
H	3.671421	-4.996742	-0.737718
H	1.981818	-2.449854	-2.089733
C	3.362632	1.201332	0.631154
C	3.450413	0.656141	1.694002
H	3.265414	1.677976	-0.320894
H	3.528374	0.152197	2.630330
C	1.755858	3.137308	-2.187506
C	1.744067	2.032671	-2.652790
H	1.757537	4.119737	-1.772208
H	1.701431	1.028949	-3.027415
C	-1.953562	0.354666	1.046050
C	-0.698435	0.075182	0.350736
H	-1.845960	0.813414	2.026954
H	0.216468	0.295301	0.898876
C	-0.555721	-0.434158	-0.913012
C	0.723566	-0.623359	-1.486590
H	-1.450386	-0.666679	-1.489541
H	1.462649	-0.346559	-0.709100
C	-2.969917	-4.357271	1.106719
C	-1.893723	-3.833065	1.079904
H	-3.921671	-4.836861	1.125888
H	-0.930265	-3.370191	1.065098
C	-3.215332	0.632356	0.244801
C	-3.166080	-0.527922	0.775219
H	-3.656537	1.397508	-0.370192
H	-3.523044	-1.525262	0.966388
C	-1.946142	2.986632	-2.163393
C	-2.992626	3.563373	-2.248456
H	-1.004235	2.483825	-2.094211
H	-3.920538	4.079451	-2.342937
C	0.569189	3.282263	1.364751
C	-0.447297	3.716888	0.904342
H	1.478554	2.883127	1.757438
H	-1.348084	4.094960	0.475836

DEC 8

1 2

C	0.941329	4.203983	-0.628538
C	0.312058	4.666165	0.280632
H	1.511845	3.804353	-1.439484
H	-0.234174	5.108841	1.084070
C	3.352246	0.934818	1.954082
C	2.686483	1.896924	1.695705
H	3.964978	0.098370	2.207760

H	2.118112	2.772351	1.462716
C	2.523937	1.268913	-2.946662
C	3.075078	1.358026	-1.886629
H	2.059407	1.199539	-3.904881
H	3.569041	1.444803	-0.942939
C	2.165928	-2.034954	-0.858051
C	1.822210	-2.297660	0.331208
H	2.832084	-1.783504	-1.665145
H	1.945331	-2.459189	1.389266
C	-2.225651	1.003221	-1.074830
C	-2.928440	2.114978	-1.217611
H	-2.648851	0.045406	-1.367482
H	-2.802498	3.176035	-1.037855
C	0.654163	-2.333507	-0.681703
C	-0.262992	-1.242675	-0.739214
H	0.276127	-3.298363	-1.005799
H	-1.253783	-1.477790	-1.123187
C	-3.229325	-2.530719	-2.720247
C	-3.379404	-2.888231	-1.586738
H	-3.121371	-2.239774	-3.741436
H	-3.526168	-3.213322	-0.580412
C	0.564638	-2.301404	3.820360
C	0.865265	-1.171185	3.559965
H	0.298085	-3.301750	4.081870
H	1.147299	-0.162197	3.349654
C	-3.584259	-1.006269	1.383044
C	-2.546490	-1.251684	1.928770
H	-4.521817	-0.790262	0.921163
H	-1.635954	-1.459847	2.449303
C	0.018309	0.069264	-0.370426
C	-0.891565	1.089237	-0.535572
H	0.995066	0.314479	0.031384
H	-0.565655	2.086987	-0.248303

DEC 9
1 2

C	0.896725	2.407726	-1.835629
C	-0.049817	2.755310	-1.186795
H	1.754530	2.131146	-2.409847
H	-0.886648	3.084069	-0.605880
C	3.165550	2.035517	2.398091
C	2.453090	2.396049	1.504744
H	3.803412	1.739914	3.201279
H	1.828532	2.725109	0.701210
C	4.554313	1.061123	-2.251012
C	4.517548	1.912954	-1.409495
H	4.622249	0.317285	-3.013273
H	4.505350	2.677471	-0.664196
C	2.603139	-0.804372	0.107811
C	2.067448	-1.914639	0.364604
H	3.354401	-0.032463	0.069775
H	1.933915	-2.914379	0.752764
C	-3.416271	0.025570	-1.147530
C	-2.933431	0.791530	-1.932443
H	-3.860164	-0.666663	-0.466731
H	-2.520131	1.474140	-2.642068
C	1.205317	-0.984848	-0.569348
C	0.072005	-0.382423	-0.009574
H	1.174024	-1.214024	-1.629229
H	-0.782310	-0.196201	-0.657527
C	-1.872497	-3.070891	-0.787707
C	-1.690227	-3.251704	0.382395
H	-2.059212	-2.922369	-1.828303

H	-1.534914	-3.448347	1.420044
C	0.980686	-5.233765	1.296225
C	1.487881	-4.928416	2.337393
H	0.523060	-5.528429	0.377850
H	1.942254	-4.693567	3.274064
C	-2.943996	3.161398	1.040511
C	-2.176558	3.796672	1.706275
H	-3.635181	2.609163	0.442087
H	-1.513182	4.384696	2.301033
C	-0.081410	0.001089	1.355855
C	-1.248655	0.490455	1.755463
H	0.751256	-0.101722	2.052256
H	-1.707857	0.865287	2.661290

DEC 10

1 2

C	-2.294845	-0.845082	-3.108130
C	-1.596234	0.039160	-2.697814
H	-2.916931	-1.617159	-3.508483
H	-0.962781	0.825732	-2.343435
C	-1.023494	3.403290	-0.738657
C	-1.150207	2.423077	-0.060398
H	-0.866554	4.286604	-1.316080
H	-1.227876	1.543708	0.544440
C	-1.841028	-0.645870	2.081244
C	-1.973015	-1.054089	0.963126
H	-1.691710	-0.304649	3.079122
H	-2.080356	-1.435222	-0.027817
C	-5.991168	0.500920	-0.134181
C	-5.010517	0.460833	0.649601
H	-6.992289	0.415093	-0.522426
H	-4.428607	0.265997	1.534683
C	-4.107478	-3.528361	-0.919369
C	-4.794191	-3.150268	-0.013893
H	-3.493640	-3.902265	-1.707782
H	-5.401762	-2.854834	0.811738
C	-8.349361	2.709416	-1.816172
C	-8.207647	1.737323	-2.501720
H	-8.517249	3.568002	-1.205131
H	-8.094647	0.866669	-3.112060
C	-5.228680	3.000313	-4.308213
C	-4.119332	2.792549	-4.712882
H	-6.220474	3.196737	-3.954237
H	-3.139558	2.599538	-5.093251
C	-4.387792	2.223747	-1.106354
C	-4.611790	0.877286	-0.813683
H	-3.742912	2.451815	-1.951136
H	-4.139437	0.137526	-1.447667
C	-4.720550	4.562512	-0.886963
C	-4.968213	3.343872	-0.429305
H	-4.154676	5.038410	-1.679091
H	-5.628027	3.182180	0.418594
C	-6.167013	-1.566130	-3.179803
C	-5.816908	-0.573157	-3.752692
H	-6.452083	-2.461815	-2.668031
H	-5.491162	0.303261	-4.271104

DEC 11

1 2

C	0.473840	2.136983	0.585875
C	0.438268	1.586381	-0.477741

H	0.547151	2.633004	1.527931
H	0.448078	1.103071	-1.432765
C	0.762590	-0.455629	-3.691116
C	0.918859	0.669951	-4.069589
H	0.632878	-1.466121	-3.373688
H	1.076360	1.665378	-4.421062
C	-1.843777	-2.025487	1.854400
C	-1.249059	-1.057392	1.472968
H	-2.340399	-2.900186	2.211564
H	-0.693388	-0.205518	1.137393
C	-3.240736	2.286064	0.281621
C	-3.243136	2.877492	1.546870
H	-2.545920	2.173363	-0.539344
H	-2.495347	3.419495	2.110447
C	-4.409064	0.334094	0.603067
C	-4.786506	-0.677695	-0.297540
H	-3.780370	0.045570	1.444641
H	-4.376432	-1.668885	-0.133739
C	-3.382023	2.313618	-3.410526
C	-2.549447	1.451585	-3.431731
H	-4.105867	3.097840	-3.421867
H	-1.794416	0.694453	-3.475071
C	-4.548026	2.500205	1.810745
C	-4.682774	1.754862	0.485998
H	-5.231155	2.589530	2.644563
H	-5.448878	2.047344	-0.229306
C	-6.167598	-0.562811	-1.017668
C	-5.184558	-0.279126	-1.754441
H	-7.187384	-0.757275	-0.735127
H	-4.634563	0.008424	-2.635349
C	-7.807156	0.878749	1.825850
C	-7.271169	0.022520	2.469161
H	-8.315806	1.642340	1.280006
H	-6.827014	-0.743037	3.065918
C	-2.075506	-2.106995	-1.980713
C	-2.478271	-2.229897	-3.102441
H	-1.709348	-2.021947	-0.979537
H	-2.820092	-2.359006	-4.105613

DEC 12

1 2

C	1.251651	2.944845	-0.148735
C	2.042526	2.249546	0.542080
H	0.641763	3.792724	-0.432778
H	2.693337	1.945830	1.343633
C	-1.783243	0.497313	1.983774
C	-1.160213	1.379512	2.503535
H	-2.348151	-0.289980	1.528247
H	-0.635296	2.167279	2.996440
C	-2.973227	-2.461138	-0.047609
C	-3.303786	-1.482534	-0.656613
H	-2.693440	-3.351480	0.477921
H	-3.616370	-0.618827	-1.201483
C	-2.148019	2.095449	-1.143585
C	-1.916829	1.906159	-2.304268
H	-2.364954	2.266279	-0.110936
H	-1.735776	1.748255	-3.344619
C	-1.922580	-5.961247	0.270672
C	-1.626221	-5.712131	1.403663
H	-2.197516	-6.208164	-0.730133
H	-1.364369	-5.510517	2.418046
C	3.691786	-0.938548	0.840124

C	4.604243	-0.296827	0.403665
H	2.894527	-1.531995	1.234796
H	5.439797	0.249329	0.025958
C	-0.984947	5.541622	-1.233896
C	-0.191815	6.201097	-0.625209
H	-1.703446	4.971710	-1.779939
H	0.498113	6.817526	-0.093160
C	0.365662	-2.252981	1.973643
C	0.667991	-3.165747	1.258385
H	0.087849	-1.452182	2.623590
H	0.925886	-4.002399	0.646682
C	1.735020	1.645390	-0.877580
C	0.891650	0.533659	-0.882331
H	2.493117	1.731566	-1.650185
H	0.131732	0.466815	-0.104852
C	0.108128	-1.524517	-1.750611
C	0.988659	-0.532272	-1.822914
H	-0.759889	-1.804796	-1.159033
H	1.771794	-0.514179	-2.577354

DEC 13

1 2

C	-3.197980	-0.884255	-1.535791
C	-2.987868	-2.054673	-1.687303
H	-3.410494	0.155887	-1.407784
H	-2.837054	-3.099887	-1.844161
C	1.190523	-0.351668	-1.289944
C	0.041591	0.442504	-1.031462
H	2.246134	-0.130634	-1.137335
H	-0.160658	1.479251	-0.809330
C	4.385232	0.843176	0.034369
C	3.608305	1.755627	0.064899
H	5.092772	0.043670	0.000338
H	2.934920	2.585804	0.079206
C	0.866831	3.947957	-1.741705
C	0.689395	4.194462	-0.582712
H	1.033267	3.769321	-2.780832
H	0.545476	4.434994	0.447776
C	4.043449	-2.502070	-1.857199
C	3.765439	-2.747203	-0.717945
H	4.317412	-2.308334	-2.870657
H	3.531308	-2.975918	0.298781
C	-0.405033	-0.827714	-0.565565
C	0.507825	-1.594235	-1.350445
H	-0.933217	-1.079296	0.353584
H	0.792808	-2.629814	-1.439844
C	-2.754919	2.823086	-0.320904
C	-3.125650	2.042284	0.509470
H	-2.440538	3.539133	-1.048544
H	-3.462383	1.355890	1.256348
C	-2.768458	-1.355426	2.148417
C	-1.882425	-0.760182	2.694153
H	-3.569549	-1.884255	1.680529
H	-1.096522	-0.231794	3.190199
C	1.088798	2.095147	2.782851
C	0.034708	2.268461	2.240332
H	2.020631	1.957494	3.285378
H	-0.912276	2.442386	1.775165
C	1.287344	-2.171341	2.274363
C	1.963078	-1.230197	1.968175
H	0.691605	-3.004343	2.576928
H	2.577578	-0.394753	1.706783

DEC 14

1 2

C	-0.627404	2.372758	1.366459
C	0.007846	1.696747	2.125059
H	-1.206881	2.970127	0.696530
H	0.555394	1.087941	2.810323
C	0.790950	0.268654	-1.318789
C	1.048819	-0.100282	-2.429390
H	0.532107	0.591279	-0.331790
H	1.256405	-0.446394	-3.417915
C	2.348397	3.854098	0.344049
C	2.872146	4.677889	-0.450824
H	1.801473	3.472625	1.191349
H	3.212251	5.613918	-0.857980
C	4.396302	1.505754	-1.924701
C	5.602307	0.961330	-1.866173
H	3.663286	1.210482	-2.670407
H	6.501982	1.054758	-1.269880
C	6.006341	4.256945	1.849541
C	6.712416	3.291330	1.790671
H	5.399765	5.131528	1.926840
H	7.359502	2.442413	1.772614
C	2.318422	3.397991	-4.588038
C	3.309641	2.928015	-5.069684
H	1.420723	3.815711	-4.185219
H	4.180872	2.517308	-5.529950
C	6.235250	5.056126	-1.773262
C	5.490778	4.895418	-2.698458
H	6.916945	5.207822	-0.965521
H	4.835499	4.756839	-3.531858
C	-0.557218	4.569657	-2.032621
C	-0.610248	3.395445	-2.265701
H	-0.543703	5.618860	-1.837716
H	-0.667010	2.349385	-2.477018
C	2.833866	3.205001	-0.996504
C	4.055144	2.530103	-0.985985
H	2.087354	2.940796	-1.737320
H	4.792405	2.810571	-0.236626
C	3.374830	0.286795	1.289993
C	3.847057	1.080555	2.053605
H	2.958524	-0.435901	0.622514
H	4.282026	1.770845	2.742724

DEC 15

1 2

C	-1.261837	3.463647	2.025276
C	-1.681155	3.593826	0.910604
H	-0.900807	3.371047	3.025583
H	-2.082109	3.728200	-0.069584
C	-3.959151	0.866032	-0.916481
C	-4.333775	1.408098	0.084683
H	-3.646659	0.384599	-1.818010
H	-4.686546	1.901810	0.963504
C	2.270071	3.274080	0.708822
C	3.441553	3.066033	0.568314
H	1.231818	3.490787	0.846491
H	4.491371	2.906756	0.456532
C	-1.137912	0.192159	1.363568
C	0.122872	-0.080758	1.038380

H	-2.036363	0.575209	0.886999
H	0.839945	-0.480955	1.751997
C	3.352499	-0.099289	2.626073
C	3.267594	-1.294203	2.644982
H	3.430372	0.965772	2.618364
H	3.200193	-2.359034	2.679044
C	-3.361964	-2.602714	0.240578
C	-2.882446	-3.662755	-0.044746
H	-3.830512	-1.674194	0.483633
H	-2.483090	-4.621074	-0.294258
C	0.684404	-3.398451	0.472354
C	1.766931	-3.581026	-0.006350
H	-0.286130	-3.259702	0.896476
H	2.730215	-3.774042	-0.423215
C	2.300565	0.806934	-1.980342
C	1.945637	-0.349644	-2.330812
H	2.704995	1.801930	-2.046941
H	1.785761	-1.197459	-2.973746
C	-1.310839	-0.452527	-3.473640
C	-1.536326	-1.294192	-2.650412
H	-1.125990	0.280566	-4.227496
H	-1.771784	-2.047424	-1.926992
C	0.532079	0.168565	-0.300240
C	1.813963	-0.094627	-0.791833
H	-0.199725	0.586025	-0.989730
H	2.550157	-0.522071	-0.118905

DEC 16

1 2

C	-0.938110	2.179200	0.797943
C	0.139332	1.851158	0.388076
H	-1.903646	2.472428	1.151596
H	1.103651	1.566729	0.028159
C	1.164724	3.060758	-2.724310
C	1.233340	2.075190	-3.401769
H	1.092252	3.936984	-2.115423
H	1.281252	1.199952	-4.008373
C	1.701725	3.246531	3.282604
C	2.608434	3.878340	3.745062
H	0.891661	2.692172	2.856465
H	3.412308	4.440372	4.162656
C	3.792990	1.910065	-0.468124
C	3.868221	1.757255	0.717418
H	3.703927	2.053397	-1.522407
H	3.925990	1.631730	1.775363
C	-3.714576	1.717026	-2.916402
C	-2.617498	1.900702	-2.472938
H	-4.690500	1.546155	-3.308507
H	-1.641220	2.068354	-2.071625
C	-2.271483	1.813996	4.463212
C	-1.922658	2.938948	4.247643
H	-2.573861	0.810308	4.656632
H	-1.611191	3.939128	4.036223
C	2.273707	5.188699	0.104189
C	1.767028	6.169697	-0.360022
H	2.725756	4.310904	0.512669
H	1.306838	7.037733	-0.774409
C	-1.787835	6.075481	2.341815
C	-0.602444	5.918317	2.418238
H	-2.843846	6.201086	2.255635
H	0.453901	5.767705	2.472131
C	-1.738164	5.225612	-2.370492

C	-1.481074	5.320916	-1.204432
H	-1.966971	5.120788	-3.406250
H	-1.251803	5.396997	-0.162862
C	-4.195864	3.687710	0.483830
C	-4.397928	3.523328	1.653149
H	-3.995108	3.816612	-0.557909
H	-4.563570	3.362296	2.694460

DEC 17

1 2

C	1.590704	1.203729	1.532837
C	1.954112	0.844329	0.448452
H	1.287310	1.510965	2.511639
H	2.314645	0.530783	-0.507941
C	0.393037	2.881545	-2.567491
C	-0.782179	3.246910	-2.865664
H	1.464045	2.802464	-2.667906
H	-1.541899	3.752464	-3.435895
C	-0.367853	2.129201	4.438861
C	0.442821	2.938747	4.789783
H	-1.075832	1.392881	4.123398
H	1.170559	3.644084	5.125525
C	3.921814	1.810027	-2.517784
C	3.440463	0.929983	-3.172576
H	4.363831	2.595239	-1.944657
H	3.044114	0.141558	-3.772909
C	-0.906979	2.749547	-0.355559
C	-0.711286	2.256763	-1.680442
H	-1.335041	2.053567	0.366131
H	-0.990467	1.221408	-1.852025
C	-4.142412	4.015394	3.229331
C	-3.668916	3.466145	4.182617
H	-4.595509	4.514462	2.401925
H	-3.268187	2.985391	5.047591
C	2.932698	5.309160	-0.887008
C	2.786460	4.348757	-0.185711
H	3.083893	6.168085	-1.502374
H	2.669711	3.483759	0.431987
C	-0.612494	4.033522	0.093781
C	-0.881855	4.352970	1.402269
H	-0.158348	4.762875	-0.567745
H	-1.332770	3.604394	2.051223
C	-2.792467	0.544381	2.098348
C	-1.793105	-0.075285	1.866563
H	-3.689230	1.081438	2.318978
H	-0.908129	-0.638029	1.663585
C	-0.958287	5.902131	3.228268
C	-0.604414	5.641598	1.979786
H	-1.456232	5.393190	4.044417
H	-0.109105	6.392400	1.366959

DEC 18

1 2

C	1.101195	-2.539866	0.384301
C	-0.136675	-2.756120	0.480297
H	2.166478	-2.656343	0.524182
H	-1.044532	-3.247449	0.785785
C	0.271320	-0.567753	-4.172576
C	0.063300	0.523798	-3.724337
H	0.455504	-1.526208	-4.605014
H	-0.116437	1.506129	-3.342735

C	0.111356	0.129690	1.456169
C	0.027557	1.421604	1.762623
H	0.197559	-0.633253	2.224276
H	-0.059640	2.358583	1.223928
C	-3.173033	-1.638734	1.720369
C	-2.863204	-2.033257	2.808589
H	-3.465447	-1.284063	0.755145
H	-2.618208	-2.394512	3.782963
C	4.721928	-2.388990	0.656609
C	4.461165	-2.563008	1.812552
H	4.979405	-2.232126	-0.367213
H	4.264319	-2.728108	2.848222
C	0.684776	3.280298	-1.281243
C	-0.502085	3.430070	-1.357276
H	1.744492	3.154888	-1.226659
H	-1.555591	3.578909	-1.447075
C	2.957627	0.466525	-1.529926
C	3.434838	0.626214	-0.442228
H	2.538657	0.328696	-2.503868
H	3.891145	0.760847	0.513502
C	-2.994858	1.919242	2.723213
C	-2.810411	3.076338	2.472671
H	-3.175186	0.891265	2.953005
H	-2.666888	4.116534	2.280489
C	-2.838650	0.295906	-1.738737
C	-3.029655	0.944738	-0.748986
H	-2.681209	-0.262537	-2.635522
H	-3.217804	1.523880	0.131250
C	0.203983	-1.536309	-0.427822
C	0.081807	-0.232177	0.079925
H	0.146504	-1.650335	-1.504913
H	-0.039519	0.577981	-0.636528

DEC 19

1 2

C	0.304289	0.572754	-0.941108
C	-0.816923	1.408453	-1.219894
H	0.349063	-0.409064	-0.476529
H	-1.887110	1.292533	-1.321659
C	4.247162	-0.244111	-1.106173
C	4.837809	0.738000	-1.456564
H	3.737228	-1.128206	-0.786016
H	5.391319	1.594069	-1.774082
C	-1.856782	-2.087527	0.045220
C	-2.658955	-1.636003	-0.722765
H	-1.148034	-2.504868	0.727791
H	-3.392781	-1.240180	-1.391882
C	1.263848	1.629384	-0.921382
C	0.074016	2.351341	-0.645724
H	2.318435	1.711381	-0.708783
H	-0.114087	3.161506	0.053431
C	1.353286	-0.872285	-3.309203
C	0.297738	-1.401194	-3.099645
H	2.299891	-0.422666	-3.517987
H	-0.635759	-1.890462	-2.923683
C	3.685398	0.190948	3.640933
C	4.164438	0.270915	2.545824
H	3.284218	0.121169	4.627872
H	4.623215	0.335894	1.583215
C	1.663539	-2.754052	-0.023156
C	1.900148	-2.176119	1.000246
H	1.464866	-3.284906	-0.928027

H	2.139751	-1.680812	1.917087
C	-4.490647	1.358342	-1.332551
C	-4.260150	1.444495	-2.505463
H	-4.713783	1.275850	-0.289277
H	-4.089701	1.532170	-3.555552
C	0.407969	0.971264	2.420266
C	-0.773421	0.797944	2.308822
H	1.460940	1.106230	2.556309
H	-1.831832	0.645938	2.244378
C	-4.484424	1.108914	2.486574
C	-4.387586	-0.014684	2.082738
H	-4.590124	2.100099	2.868294
H	-4.310680	-1.020732	1.733742

DEC 20

1 2

C	0.976421	1.724873	1.123014
C	1.379431	2.158112	2.412161
H	1.128896	0.853432	0.505026
H	2.104920	1.742977	3.105590
C	-0.629632	4.132568	-4.688393
C	-0.999645	3.048093	-4.339831
H	-0.311744	5.100676	-5.006588
H	-1.349150	2.080982	-4.055912
C	0.951901	3.137894	0.910172
C	0.849720	3.468629	2.295720
H	1.329846	3.725913	0.076998
H	0.855147	4.379490	2.871619
C	4.517300	2.497271	1.283531
C	4.596405	3.371297	2.099717
H	4.467347	1.708350	0.562080
H	4.712275	4.135053	2.836590
C	-2.054994	1.166185	3.047437
C	-2.058491	2.213677	3.628203
H	-2.083832	0.220764	2.553052
H	-2.095999	3.133252	4.168841
C	3.180562	0.325458	-1.347520
C	3.293449	-0.639865	-0.646782
H	3.098513	1.181340	-1.982517
H	3.409165	-1.519321	-0.052942
C	-1.699058	3.498671	0.129276
C	-1.206009	4.058467	-0.811631
H	-2.162279	3.003655	0.956933
H	-0.807001	4.573226	-1.661733
C	2.201926	3.791215	-2.470515
C	3.138364	4.057413	-1.770254
H	1.376941	3.570073	-3.118584
H	3.983414	4.298411	-1.162988
C	-0.717516	7.146252	-2.659008
C	0.464347	6.991662	-2.541442
H	-1.767695	7.297883	-2.774125
H	1.519001	6.865548	-2.437245
C	1.210408	6.601515	0.794811
C	2.353776	6.471581	1.127943
H	0.199825	6.742691	0.478680
H	3.378384	6.398068	1.415374

EICO 1

1 2

C	4.391113	5.326055	10.246044

C	5.531264	5.670584	10.385625
H	3.370684	5.020604	10.142717
H	6.552096	5.956146	10.524836
C	2.282537	9.658657	10.508928
C	3.118761	8.814731	10.661735
H	1.539687	10.416465	10.398312
H	3.861854	8.061492	10.813984
C	9.409128	6.587980	10.081143
C	9.005294	5.541360	9.660222
H	9.786230	7.508565	10.466582
H	8.643253	4.600645	9.301976
C	8.018680	1.994943	8.708727
C	6.951449	2.389837	9.084073
H	8.967666	1.625526	8.389279
H	5.998356	2.721925	9.438335
C	0.750191	4.504979	9.765383
C	0.871613	5.696221	9.721682
H	0.642307	3.444441	9.821773
H	0.982421	6.758381	9.701192
C	6.718708	8.551038	8.597122
C	6.008050	9.517082	8.603985
H	7.357784	7.693150	8.614348
H	5.376878	10.378633	8.632585
C	3.047046	3.492477	2.190247
C	4.194034	3.839192	2.187166
H	2.027920	3.174628	2.184438
H	5.218029	4.146701	2.167235
C	6.512785	1.828135	4.042106
C	6.765541	2.095396	5.182668
H	6.293509	1.564851	3.031463
H	6.989880	2.308943	6.206712
C	4.175039	8.241293	0.833838
C	3.705490	7.153803	1.019544
H	4.585657	9.207907	0.641990
H	3.296104	6.177252	1.176177
C	3.267477	1.720478	9.450078
C	3.370311	1.957795	10.619319
H	3.177579	1.505602	8.408912
H	3.463570	2.156137	11.663530
C	2.227904	7.457026	6.814134
C	3.182377	7.326021	7.527764
H	1.366147	7.544502	6.188040
H	4.017567	7.203592	8.186188
C	8.281084	9.185722	5.438159
C	8.319299	8.453345	4.489878
H	8.259588	9.833927	6.286614
H	8.369176	7.793310	3.650527
C	3.119675	10.958924	7.093245
C	3.427919	12.004419	6.594808
H	2.846343	10.031549	7.551399
H	3.688201	12.947632	6.167985
C	7.483833	5.694291	6.572071
C	6.436161	5.210694	6.897358
H	8.420409	6.132912	6.305076
H	5.506663	4.767307	7.187948
C	5.011165	6.975802	4.382151
C	4.004819	7.887724	3.962071
H	5.111328	5.907732	4.481752
H	2.940445	7.733196	3.800374
C	1.554030	10.000863	2.613492
C	1.504904	10.423358	3.733479
H	1.590698	9.633332	1.611944
H	1.462701	10.821857	4.723824

C	3.491269	3.349056	5.905742
C	2.826703	3.917870	6.727042
H	4.092357	2.832674	5.186838
H	2.227563	4.415912	7.461378
C	7.572153	5.133312	2.849937
C	7.256678	5.862716	1.952380
H	7.838740	4.460790	3.637704
H	6.977333	6.496814	1.139702
C	0.532409	6.601576	3.437547
C	1.172805	5.764318	4.008597
H	-0.055089	7.338585	2.936460
H	1.732721	5.015180	4.529562
C	4.722824	9.064947	4.308627
C	5.371603	8.117434	5.150930
H	4.509353	10.120615	4.342929
H	5.682343	8.196894	6.190899

EICO 2
1 2

C	4.097708	5.526044	10.558401
C	5.128466	6.254277	10.579944
H	3.513609	4.620241	10.606098
H	6.171324	6.518969	10.655740
C	3.559532	7.476441	8.997945
C	3.801262	7.031571	10.313819
H	2.990136	8.396937	8.880819
H	3.377324	7.622288	11.119454
C	8.703017	7.118624	9.720121
C	8.361087	6.082483	9.224615
H	9.022922	8.036997	10.160295
H	8.059286	5.155802	8.785101
C	6.223424	3.874666	7.170562
C	5.855850	3.378837	8.198626
H	6.561969	4.289410	6.241887
H	5.525457	2.913098	9.100562
C	0.474518	5.911127	9.113283
C	0.339925	6.888460	8.433047
H	0.571615	5.027187	9.705054
H	0.204172	7.743717	7.805983
C	6.179629	9.374334	8.818597
C	5.700385	10.120605	9.624524
H	6.609301	8.714891	8.094352
H	5.282952	10.795669	10.338486
C	3.171474	3.438733	1.964627
C	4.339033	3.700316	2.029949
H	2.133279	3.202312	1.897813
H	5.379873	3.940929	2.084774
C	4.731017	0.880191	4.253957
C	5.164616	0.830440	5.369251
H	4.345929	0.930587	3.259691
H	5.553503	0.778251	6.360877
C	7.151302	6.346178	0.706606
C	5.963537	6.371715	0.553506
H	8.210754	6.333619	0.839520
H	4.905682	6.384666	0.414494
C	2.407479	2.759440	9.114274
C	2.284056	2.340734	10.230933
H	2.504488	3.115259	8.108267
H	2.160571	1.938035	11.211903
C	3.720823	7.424745	6.654665
C	4.006114	6.834900	7.813316
H	3.213316	8.322080	6.312188
H	4.561688	5.900471	7.854380

C	9.621459	7.848769	2.985327
C	10.383580	6.927748	2.910057
H	8.946850	8.675027	3.048955
H	11.071964	6.115472	2.842811
C	2.307856	10.850742	8.784022
C	1.497413	10.532732	9.607921
H	3.026073	11.137486	8.044120
H	0.769618	10.271699	10.344147
C	7.603756	7.504212	5.961931
C	6.638859	7.640177	5.262616
H	8.473084	7.375629	6.569409
H	5.801174	7.756131	4.605842
C	3.663868	8.162153	3.067214
C	3.454336	6.998444	3.268361
H	3.881347	9.191703	2.881556
H	3.277050	5.954873	3.422783
C	0.742306	8.896282	5.154556
C	0.424773	9.683855	6.000290
H	1.032688	8.214974	4.383805
H	0.138186	10.391720	6.746637
C	6.623812	10.706781	2.711658
C	6.583696	9.691214	2.077276
H	6.658112	11.622078	3.257908
H	6.548790	8.785912	1.507198
C	7.759852	4.525991	3.762334
C	7.417485	3.384656	3.893729
H	8.085666	5.536941	3.641399
H	7.102034	2.370606	4.011712
C	1.991151	4.575630	5.945527
C	2.893438	3.895567	5.543457
H	1.180109	5.161047	6.320320
H	3.680395	3.269030	5.177753
C	3.706402	11.035587	5.375528
C	4.791306	10.785197	5.818628
H	2.741064	11.255649	4.976130
H	5.764794	10.573496	6.202006

EICO 3
1 2

C	4.383527	5.882568	9.918952
C	5.327195	6.621878	9.978605
H	3.571448	5.189677	9.859339
H	6.186791	7.257973	10.041424
C	1.765158	8.011988	8.723959
C	2.893248	8.388661	9.327193
H	1.085917	7.320596	9.217223
H	3.749396	9.028484	9.138029
C	8.597239	8.604923	9.811378
C	8.629174	7.522248	9.298927
H	8.582011	9.569302	10.266491
H	8.649805	6.555745	8.840384
C	8.887068	4.285849	7.601867
C	7.853985	3.802448	7.969526
H	9.802205	4.731247	7.275097
H	6.934162	3.357227	8.282987
C	0.966623	4.067459	9.157673
C	1.336950	4.469397	8.090616
H	0.646727	3.688656	10.103467
H	1.676258	4.808341	7.134022
C	5.847404	9.803785	7.895216
C	5.428614	10.826976	8.358754
H	6.253154	8.904211	7.478753
H	5.086907	11.751823	8.767558

C	-0.140639	4.928531	4.729762
C	0.678922	4.224424	4.212243
H	-0.886663	5.538097	5.191104
H	1.391872	3.578389	3.749297
C	1.814344	1.502528	6.091804
C	2.779963	0.962561	5.632490
H	0.945201	1.967334	6.500839
H	3.637740	0.473863	5.226302
C	6.466517	7.045747	2.035667
C	6.032334	6.019858	2.476157
H	6.855771	7.957527	1.641076
H	5.670157	5.096912	2.875885
C	4.308530	2.244071	8.899842
C	4.920135	2.533197	9.888469
H	3.758164	1.964786	8.026918
H	5.468304	2.784558	10.768673
C	3.411139	6.307129	5.377430
C	3.962736	6.230799	6.439806
H	2.935858	6.366136	4.419919
H	4.465146	6.143801	7.381578
C	11.543854	7.112723	7.300915
C	11.336512	6.686044	6.201236
H	11.731886	7.496472	8.278263
H	11.146295	6.294183	5.225841
C	2.087960	9.389242	6.616186
C	1.372215	8.522784	7.455778
H	3.102282	9.684675	6.868977
H	0.381208	8.224300	7.110750
C	7.994912	7.716060	5.906010
C	7.304456	6.743392	5.786331
H	8.638053	8.559889	6.025169
H	6.719040	5.855511	5.674703
C	2.687608	7.527293	2.101279
C	1.562249	7.125805	2.198832
H	3.698913	7.861919	2.012039
H	0.567303	6.744938	2.264602
C	1.203281	10.375457	5.775534
C	1.673603	9.413201	5.109376
H	0.702885	11.309047	5.961011
H	1.914242	8.828429	4.233968
C	6.441430	3.214655	4.861382
C	5.260748	3.274841	5.058142
H	7.496887	3.174412	4.694962
H	4.212090	3.309251	5.249944
C	9.401621	4.825647	3.546917
C	10.169026	3.967651	3.878345
H	8.711666	5.584707	3.247406
H	10.858809	3.204943	4.162735
C	-1.630912	6.474319	7.602845
C	-2.223572	7.370049	7.071629
H	-1.113192	5.662091	8.067979
H	-2.783614	8.153385	6.611210
C	4.584543	10.372627	3.807242
C	5.085336	9.389072	4.274490
H	4.159645	11.254586	3.383380
H	5.560811	8.517045	4.672795

EICO 4
1 2

C	2.087491	2.675779	2.720306
C	2.909777	2.415285	1.887698
H	1.361672	2.896440	3.471350
H	3.663173	2.181486	1.163757

C	5.777396	2.233062	-0.217792
C	5.831221	1.037237	-0.169024
H	5.745546	3.301398	-0.240768
H	5.914348	-0.026914	-0.128240
C	-1.012693	2.648055	0.632446
C	-2.059756	2.069498	0.722497
H	-0.091034	3.184051	0.539333
H	-3.006295	1.574362	0.786366
C	0.706910	-4.466365	0.387615
C	1.777965	-4.181767	-0.069249
H	-0.245515	-4.732342	0.796517
H	2.747413	-3.950649	-0.454757
C	-4.242276	-1.157108	2.381113
C	-3.893596	-0.760828	3.457247
H	-4.562932	-1.524023	1.427960
H	-3.592091	-0.411048	4.419434
C	-0.912447	1.136736	4.010865
C	-0.203698	0.300248	4.493908
H	-1.538829	1.882840	3.574048
H	0.429007	-0.441696	4.928233
C	4.747755	5.436380	1.267799
C	5.059275	5.985770	0.250218
H	4.471117	4.958430	2.181311
H	5.340670	6.495333	-0.644294
C	2.822779	-0.995683	2.983197
C	2.362411	-2.066767	2.703193
H	3.217464	-0.035855	3.237567
H	1.963964	-3.023700	2.442951
C	-5.128122	1.625686	-1.124255
C	-5.633773	1.226660	-0.114185
H	-4.689015	1.997499	-2.024528
H	-6.102099	0.880035	0.780289
C	-2.611563	3.894923	-2.453407
C	-2.352682	3.427733	-3.525788
H	-2.842703	4.317150	-1.500709
H	-2.119709	3.026216	-4.486870
C	0.225451	-0.439791	0.693386
C	0.862155	-0.556546	-0.547063
H	0.673883	0.239375	1.417399
H	0.431692	-1.194815	-1.311990
C	1.906564	4.453936	-1.041470
C	0.963897	4.234990	-1.749475
H	2.742606	4.687716	-0.416234
H	0.115318	4.055335	-2.375512
C	1.422652	2.094211	-4.390763
C	0.583373	1.247959	-4.272699
H	2.167761	2.849411	-4.504943
H	-0.171189	0.498969	-4.172946
C	-2.140360	0.046082	-2.388995
C	-2.258520	-0.973471	-3.008716
H	-2.034404	0.970166	-1.860456
H	-2.360364	-1.885623	-3.555062
C	2.410657	-0.319288	-0.556269
C	1.749570	0.657611	-0.998859
H	3.267909	-0.922694	-0.307379
H	1.561617	1.617966	-1.452706
C	5.113756	-2.679020	0.378225
C	5.321501	-2.679508	-0.802063
H	4.934286	-2.681863	1.431527
H	5.532214	-2.691438	-1.848721
C	-1.279565	-4.697897	-2.440821
C	-2.421843	-4.398225	-2.237308
H	-0.267408	-4.984635	-2.618921

H	-3.438155	-4.124140	-2.048123
C	-2.866942	-4.560011	1.993325
C	-2.737340	-5.442379	1.192933
H	-3.006645	-3.781314	2.711426
H	-2.626906	-6.232563	0.483531
C	-5.211771	-2.194012	-1.032417
C	-5.320879	-3.208579	-0.403807
H	-5.130651	-1.276900	-1.574320
H	-5.417527	-4.109811	0.160357
C	-0.961074	-1.123218	1.105265
C	-1.562700	-2.032360	0.347483
H	-1.369502	-0.869020	2.081016
H	-1.478195	-2.551719	-0.599621

EICO 5
1 2

C	4.735817	3.868728	11.436110
C	5.782041	4.210202	10.962240
H	3.810483	3.554744	11.866213
H	6.711068	4.521198	10.535625
C	4.531175	8.585745	9.877637
C	4.869102	7.663723	10.565125
H	4.211919	9.413369	9.280404
H	5.156550	6.841495	11.184117
C	7.669655	6.291848	8.454926
C	7.640438	5.211739	7.935071
H	7.713605	7.262848	8.901009
H	7.614271	4.251415	7.467412
C	4.365004	4.675407	6.673847
C	4.273981	3.855773	7.717749
H	4.346087	4.309347	5.650779
H	4.300237	3.934408	8.798776
C	3.481750	1.639447	4.580774
C	2.864674	1.581638	5.607084
H	4.005670	1.697647	3.650994
H	2.310822	1.529187	6.520157
C	8.414296	9.881472	7.869262
C	7.682326	10.040172	8.804267
H	9.073605	9.747647	7.039944
H	7.048638	10.189055	9.650042
C	3.374728	3.643473	1.490085
C	4.557429	3.733141	1.659311
H	2.323786	3.556738	1.325678
H	5.613606	3.806193	1.799939
C	5.932132	1.358854	7.548541
C	6.643412	1.541126	8.496021
H	5.292600	1.173472	6.712045
H	7.271792	1.675755	9.347980
C	8.071558	6.278627	1.123086
C	6.942106	6.359611	0.732010
H	9.080383	6.201136	1.462768
H	5.935702	6.432205	0.383391
C	1.494767	2.916981	9.072020
C	2.146374	1.943156	9.322815
H	0.914531	3.788225	8.859095
H	2.722039	1.073394	9.551445
C	1.322933	6.514469	7.612221
C	1.648119	6.864689	8.711722
H	1.040301	6.193210	6.631267
H	1.945445	7.175133	9.689096
C	8.322278	8.360520	4.263941
C	8.560745	7.524661	5.089640
H	8.124472	9.103005	3.519218

H	8.768802	6.778685	5.825672
C	2.706034	11.363482	7.721660
C	2.046463	10.835004	8.569682
H	3.276540	11.858727	6.968121
H	1.448965	10.385652	9.330884
C	4.669889	7.024247	5.920150
C	4.517525	6.065389	6.926111
H	4.714609	6.701832	4.884572
H	4.507721	6.406970	7.959364
C	4.480129	8.345901	2.390612
C	3.847301	7.329379	2.453635
H	5.058396	9.241301	2.302338
H	3.291337	6.417267	2.503817
C	1.355597	9.393031	4.246190
C	0.675923	9.536502	5.222517
H	1.964325	9.258247	3.378003
H	0.068576	9.683822	6.087605
C	7.230579	11.089911	1.684151
C	7.413702	10.017696	1.182423
H	7.081763	12.059493	2.103308
H	7.578386	9.059318	0.738317
C	7.030613	4.556350	4.125516
C	7.085469	3.477672	4.644860
H	7.004987	5.509548	3.640809
H	7.153101	2.517148	5.106206
C	0.979090	5.837215	4.068546
C	1.483877	4.768814	4.270727
H	0.524854	6.788963	3.896829
H	1.917740	3.805389	4.439330
C	4.079804	8.448310	6.221630
C	5.334781	8.381517	6.318526
H	3.088368	8.870692	6.183634
H	6.348157	8.720975	6.452508

EICO 6
1 2

C	2.666329	4.942673	-0.660032
C	3.482394	5.053090	0.210251
H	1.937893	4.857911	-1.435766
H	4.215046	5.147775	0.981537
C	4.284071	0.212632	-0.902971
C	4.096455	1.339739	-0.539198
H	4.443935	-0.788026	-1.243588
H	3.943345	2.349374	-0.220430
C	5.943139	3.016221	2.168414
C	4.933143	2.986831	2.811180
H	6.850753	3.044303	1.608164
H	4.034726	2.951290	3.386375
C	1.104315	1.908579	2.866657
C	1.309713	2.793868	2.084493
H	0.941501	1.109844	3.556932
H	1.528576	3.585314	1.397355
C	-4.867398	2.159109	-2.387463
C	-4.000691	2.929207	-2.691048
H	-5.639505	1.469908	-2.122727
H	-3.233318	3.619669	-2.964109
C	4.036039	-0.378837	2.967396
C	3.416888	-0.968795	3.806835
H	4.592468	0.165016	2.233834
H	2.882890	-1.486733	4.572189
C	-5.305848	-1.368150	-1.472643
C	-5.525237	-0.970698	-0.363698
H	-5.109221	-1.715520	-2.462883

H	-5.712098	-0.622882	0.629274
C	-3.220111	3.419581	1.001840
C	-2.390045	3.521923	1.860463
H	-3.956286	3.343729	0.229878
H	-1.650663	3.622844	2.625003
C	-4.164503	0.378726	2.960041
C	-4.374571	-0.781090	3.176419
H	-3.985283	1.415137	2.769620
H	-4.563642	-1.814257	3.368103
C	-0.164483	3.175284	-2.508068
C	-0.646580	3.579794	-1.487600
H	0.274341	2.804576	-3.408935
H	-1.091014	3.937437	-0.583159
C	1.039866	0.163986	-4.133575
C	2.071029	0.606816	-3.712610
H	0.115431	-0.225319	-4.502641
H	2.990127	0.994180	-3.328901
C	0.097221	-2.081546	3.083843
C	-0.810064	-1.393085	3.458305
H	0.899558	-2.705125	2.747911
H	-1.637415	-0.799846	3.784728
C	2.140947	-4.286867	0.958744
C	3.055043	-3.799937	1.561657
H	1.336147	-4.725447	0.408974
H	3.866851	-3.366853	2.104289
C	-1.060000	0.440812	0.301178
C	0.514938	0.137219	-0.380503
H	-0.807815	1.161207	1.081798
H	0.672088	1.052219	-0.956047
C	-2.912898	-3.962094	1.829497
C	-3.816082	-3.806902	1.057046
H	-2.119654	-4.098166	2.531288
H	-4.626960	-3.660826	0.376810
C	-0.942781	-4.370308	-1.371574
C	-0.040392	-4.461336	-2.155802
H	-1.750107	-4.313896	-0.671425
H	0.771509	-4.550368	-2.845114
C	-2.048435	-0.598339	0.280714
C	-2.185681	0.389486	-0.582018
H	-2.419942	-1.510338	0.723313
H	-2.750885	0.876729	-1.361200
C	-2.190063	-1.545637	-3.601188
C	-2.685530	-0.525152	-3.987561
H	-1.755778	-2.462589	-3.263096
H	-3.140739	0.380398	-4.325427
C	0.908310	-1.210327	-0.665782
C	1.427444	-0.634451	0.403197
H	0.853291	-2.060798	-1.325609
H	2.093636	-0.662223	1.251885
C	3.531151	-3.417355	-2.191265
C	3.240513	-2.715987	-3.118425
H	3.793113	-4.041304	-1.365424
H	2.988574	-2.085959	-3.942840

EICO 7
1 2

C	-0.777027	-0.966651	1.523574
C	-1.306225	0.238294	0.980172
H	-1.001414	-2.000986	1.275560
H	-2.155261	0.515645	0.375657
C	-4.495740	-0.679357	-0.454264
C	-4.625971	0.453774	-0.824221
H	-4.382899	-1.695823	-0.143274

H	-4.765561	1.465304	-1.144183
C	0.868897	-1.700080	-1.408556
C	1.732368	-1.931825	-0.608976
H	0.117520	-1.501719	-2.144427
H	2.522734	-2.120393	0.086454
C	-5.212217	4.210976	-0.346138
C	-5.142559	4.087095	-1.535563
H	-5.285350	4.338673	0.710910
H	-5.093996	3.984054	-2.597456
C	4.133931	-0.757976	2.062635
C	3.918024	-1.854067	2.497684
H	4.358480	0.208391	1.663221
H	3.738948	-2.836732	2.874314
C	0.942258	-3.545052	3.417780
C	0.687135	-4.108633	2.391494
H	1.172151	-3.041815	4.332484
H	0.459707	-4.621085	1.482277
C	-2.736296	-3.940573	0.760821
C	-2.327832	-3.669781	-0.333105
H	-3.115568	-4.195464	1.725430
H	-1.989844	-3.432685	-1.320031
C	-3.213284	2.492906	2.765237
C	-4.177149	1.959185	2.293212
H	-2.356033	2.970075	3.188666
H	-5.034371	1.482185	1.870861
C	4.285370	-1.308740	-2.896301
C	5.162452	-0.706413	-2.344440
H	3.517538	-1.858429	-3.397963
H	5.947575	-0.179469	-1.846510
C	1.801688	-3.590153	-4.842725
C	1.575032	-2.462327	-5.176132
H	2.013748	-4.597486	-4.562744
H	1.376034	-1.454136	-5.467104
C	0.400311	-0.381434	2.063133
C	0.069415	0.573903	1.060402
H	1.306093	-0.734612	2.528978
H	0.717629	1.095713	0.360813
C	-2.421003	-1.500706	-3.281614
C	-1.707945	-2.234852	-3.906098
H	-3.071359	-0.850357	-2.736145
H	-1.063725	-2.875374	-4.469308
C	0.366521	0.950884	-4.110743
C	1.552672	0.987922	-3.942544
H	-0.689593	0.913411	-4.273337
H	2.612944	1.012417	-3.814295
C	3.091890	1.517392	-0.600615
C	2.934085	2.496071	0.074380
H	3.259736	0.642503	-1.196449
H	2.804207	3.365410	0.680209
C	-1.570047	3.276013	-0.798171
C	-0.520272	3.033497	-1.324932
H	-2.519167	3.506536	-0.361212
H	0.406078	2.816220	-1.813653
C	-3.808374	1.567976	-4.475438
C	-3.081123	2.330667	-3.905913
H	-4.453444	0.894790	-4.994809
H	-2.429653	3.008346	-3.399152
C	0.660518	2.832750	3.246718
C	0.295176	3.486919	2.310793
H	0.986539	2.254778	4.085419
H	-0.028796	4.070419	1.476346
C	6.651251	1.635609	0.198196
C	7.079260	0.608955	0.641385

H	6.280492	2.553857	-0.197726
H	7.469501	-0.300865	1.039146
C	1.949837	-0.272227	5.323230
C	0.761052	-0.350340	5.453095
H	3.010227	-0.206448	5.215126
H	-0.297072	-0.429200	5.577792
C	-2.784750	-1.130196	3.855629
C	-2.251657	-2.189881	4.029686
H	-3.262405	-0.184424	3.708291
H	-1.780663	-3.134326	4.192716

EICO 8
1 2

C	6.054438	1.544232	-1.041290
C	5.947956	2.507498	-0.335860
H	6.165576	0.688896	-1.676334
H	5.858834	3.363529	0.296104
C	2.883289	4.658076	1.192244
C	3.322545	3.756337	1.848579
H	2.485324	5.465998	0.617223
H	3.700242	2.952586	2.441515
C	0.280311	5.535341	-1.333926
C	1.318078	5.604810	-1.929265
H	-0.653264	5.478206	-0.818718
H	2.225806	5.692967	-2.486434
C	2.472396	2.226726	-1.472118
C	2.969763	1.859745	-2.568149
H	2.871185	2.690081	-0.574166
H	3.215061	1.473156	-3.543344
C	-2.641135	3.166017	-1.145355
C	-2.242084	3.119964	-0.015369
H	-3.008755	3.188065	-2.150379
H	-1.890747	3.076871	0.994780
C	-0.024829	2.687811	2.825410
C	-0.913264	2.081306	3.354758
H	0.773372	3.238498	2.375260
H	-1.697136	1.542172	3.847179
C	2.101503	-1.481426	-2.743333
C	2.326948	-1.820754	-1.616019
H	1.923687	-1.185104	-3.755057
H	2.531379	-2.147773	-0.617975
C	2.899841	-3.519503	1.697571
C	2.591725	-4.357612	0.899382
H	3.178215	-2.782625	2.417745
H	2.331064	-5.117301	0.196577
C	0.852961	2.056683	-1.386791
C	0.496063	0.978404	-0.860614
H	0.339032	2.927644	-1.781616
H	0.287070	0.028677	-0.396511
C	-1.181756	-1.005897	1.516218
C	-0.381302	-1.875330	1.315422
H	-1.917034	-0.253192	1.701130
H	0.333260	-2.657446	1.167556
C	2.325581	0.216842	1.830362
C	3.357717	0.255928	1.220835
H	1.407387	0.184034	2.377583
H	4.283993	0.313521	0.690765
C	5.728181	-1.401981	-3.317263
C	6.597597	-0.773742	-3.851491
H	4.947545	-1.964662	-2.854041
H	7.380795	-0.224078	-4.325101
C	-2.304730	-0.289185	-1.904182

C	-1.642353	-0.841240	-2.736871
H	-2.913297	0.180592	-1.160208
H	-1.070128	-1.361111	-3.472201
C	3.563456	5.696430	-5.007790
C	2.668628	6.489300	-5.084034
H	4.371107	4.998104	-4.956706
H	1.883820	7.208537	-5.157246
C	-4.047562	1.806688	-4.297152
C	-3.790313	2.888392	-4.742172
H	-4.282332	0.840490	-3.909695
H	-3.569012	3.846002	-5.157694
C	3.421288	-0.043306	-5.780783
C	2.417255	0.564630	-6.026333
H	4.321209	-0.580366	-5.566797
H	1.524755	1.108859	-6.246721
C	-4.799627	0.828533	0.849626
C	-4.698999	-0.359809	0.964131
H	-4.897060	1.885582	0.737146
H	-4.623839	-1.419340	1.071719
C	6.015975	2.951499	-4.231918
C	5.567199	2.630494	-5.295554
H	6.426290	3.234457	-3.287659
H	5.176800	2.347455	-6.247964
C	-3.818913	0.056522	4.532799
C	-2.973534	-0.297349	5.303880
H	-4.581956	0.359439	3.849733
H	-2.235673	-0.617009	6.005905
C	-0.275798	2.528394	-4.469582
C	0.585718	3.329893	-4.701453
H	-1.069521	1.839821	-4.270588
H	1.343989	4.053655	-4.919507

EICO 9

1 2

C	-2.687351	-1.477175	1.187204
C	-3.201214	-0.420696	0.950312
H	-2.229995	-2.419566	1.403880
H	-3.673890	0.519051	0.771442
C	0.327557	4.346748	-2.849752
C	0.783381	4.647702	-1.782693
H	-0.074893	4.082640	-3.803394
H	1.183826	4.930433	-0.830892
C	-4.827586	-3.789577	0.020583
C	-4.450270	-4.605499	-0.771678
H	-5.168528	-3.075536	0.739696
H	-4.139464	-5.339941	-1.480551
C	-2.103862	4.990183	0.441264
C	-2.204360	4.248139	1.377423
H	-2.017999	5.657073	-0.388173
H	-2.305634	3.591365	2.215632
C	-0.076716	0.996175	-1.594622
C	0.241450	0.006580	-0.661457
H	0.701175	1.441701	-2.206763
H	-0.588340	-0.406204	-0.089980
C	-0.682075	-2.443770	-2.655221
C	-1.729890	-2.541204	-2.078944
H	0.256903	-2.363692	-3.163786
H	-2.665269	-2.643852	-1.567602
C	0.454290	1.965540	2.068376
C	-0.269700	1.049682	2.341997
H	1.094204	2.794773	1.851609
H	-0.913189	0.235284	2.598858

C	2.537375	5.251055	1.544028
C	1.374406	5.452076	1.749119
H	3.580726	5.097694	1.381127
H	0.337915	5.632227	1.935118
C	0.166736	-3.709470	0.724280
C	0.271628	-3.459121	1.891618
H	0.053130	-3.934284	-0.313479
H	0.337321	-3.248483	2.936890
C	-1.540265	1.001932	-2.142312
C	-1.335597	1.870709	-1.252849
H	-2.067662	0.456582	-2.908543
H	-1.515876	2.705360	-0.592832
C	2.458911	-1.507382	-4.338218
C	2.972401	-2.144854	-3.462153
H	1.996602	-0.949193	-5.123326
H	3.431564	-2.715212	-2.682267
C	2.621511	-0.105946	-1.044808
C	1.546135	-0.520231	-0.389926
H	2.923023	0.594386	-1.816183
H	1.628664	-1.292050	0.371730
C	-3.687960	1.512731	3.872217
C	-4.504940	2.268228	3.427945
H	-2.975217	0.831869	4.286816
H	-5.236555	2.949615	3.054716
C	0.723675	1.535657	-5.161154
C	-0.364379	1.108644	-5.426777
H	1.700917	1.908154	-4.938096
H	-1.332398	0.729295	-5.675374
C	4.739104	-2.686267	-0.205569
C	3.811066	-3.347358	0.163527
H	5.585084	-2.116383	-0.517854
H	2.991233	-3.935507	0.513636
C	4.194044	1.718882	-3.756886
C	3.636488	2.688130	-3.325674
H	4.699412	0.862449	-4.146240
H	3.136680	3.559413	-2.960389
C	-0.951395	-1.185179	4.985643
C	-1.940390	-1.726163	4.577439
H	-0.077125	-0.704516	5.364687
H	-2.832191	-2.207637	4.231913
C	-2.475967	-5.193935	3.939562
C	-2.886692	-5.181321	2.814786
H	-2.112314	-5.216689	4.942342
H	-3.262335	-5.179141	1.814478
C	-5.502779	-2.964157	3.644078
C	-5.474121	-1.773305	3.517662
H	-5.527193	-4.023802	3.766876
H	-5.447916	-0.710111	3.422830
C	-3.424209	-0.890186	-4.768370
C	-3.981778	0.162233	-4.896687
H	-2.938104	-1.833636	-4.651708
H	-4.498563	1.086572	-5.028596

EICO 10

1 2

C	-3.142912	3.168362	0.485010
C	-2.606800	3.383133	-0.566302
H	-3.639967	2.983453	1.414932
H	-2.131668	3.596822	-1.502230
C	2.074852	1.456009	-2.073546
C	1.621941	1.932028	-1.071561
H	2.499150	1.039081	-2.959378
H	1.241567	2.361255	-0.168881

C	-1.891706	-1.521363	-1.480535
C	-2.739113	-0.535231	-1.066621
H	-2.266938	-2.542167	-1.526848
H	-2.416351	0.499276	-1.001444
C	-5.048086	0.024156	-0.311556
C	-4.057117	-0.880614	-0.732598
H	-4.791523	1.077158	-0.235280
H	-4.337781	-1.930064	-0.799670
C	-8.168348	-3.549384	0.143899
C	-7.324448	-4.380896	0.323928
H	-8.923683	-2.807201	-0.005659
H	-6.573954	-5.124508	0.476373
C	-7.703043	2.657853	-0.529153
C	-6.688314	3.202172	-0.861572
H	-8.605059	2.164933	-0.234807
H	-5.782099	3.688034	-1.151488
C	-8.621604	0.988453	-3.497859
C	-8.413375	-0.143114	-3.832856
H	-8.805502	2.000867	-3.212588
H	-8.222736	-1.146454	-4.144511
C	-4.881751	1.373055	-3.609477
C	-3.710149	1.577702	-3.755956
H	-5.930228	1.192858	-3.503443
H	-2.674228	1.792319	-3.904161
C	0.868902	3.349782	2.443593
C	0.111678	4.102131	1.900181
H	1.545168	2.683952	2.931751
H	-0.558838	4.778479	1.419120
C	-5.995999	-2.845218	-2.876964
C	-5.369069	-2.169543	-3.643446
H	-6.556917	-3.435900	-2.183207
H	-4.819814	-1.559276	-4.326770
C	-7.652966	-1.024754	3.955613
C	-7.984253	-2.064464	3.460017
H	-7.357495	-0.099839	4.400788
H	-8.282289	-2.991354	3.020337
C	-10.208370	-0.265343	-0.422161
C	-9.843094	-0.306469	0.718393
H	-10.540589	-0.224893	-1.436081
H	-9.527974	-0.356754	1.738489
C	-0.513852	-1.296160	-1.861656
C	0.238491	-2.292544	-2.289642
H	-0.108755	-0.288401	-1.777728
H	0.150169	-3.356617	-2.470742
C	-3.772035	-4.872622	-0.937341
C	-3.566624	-4.439517	0.161027
H	-3.957030	-5.269834	-1.910679
H	-3.391116	-4.049759	1.141269
C	-6.171398	-0.554063	0.601169
C	-6.530110	-0.357486	-0.593050
H	-6.271726	-0.800373	1.644067
H	-7.198134	-0.284244	-1.435114
C	-5.860081	2.128300	2.667907
C	-4.994111	1.613066	3.317783
H	-6.616455	2.598187	2.077317
H	-4.209679	1.175120	3.899229
C	-4.193567	-2.157749	3.107478
C	-3.161976	-1.705220	2.696669
H	-5.115812	-2.540497	3.487845
H	-2.233223	-1.296092	2.355551
C	-0.470628	0.393432	1.442857
C	0.254769	-0.559594	1.422809
H	-1.092771	1.261614	1.464499

H	0.914659	-1.397970	1.409197
C	-0.464455	3.787860	-3.529181
C	-1.416488	4.302969	-4.042494
H	0.392498	3.340591	-3.071790
H	-2.252759	4.771326	-4.511721
C	-1.860750	0.552676	5.102533
C	-1.574580	1.627824	4.659443
H	-2.106543	-0.404036	5.505874
H	-1.299365	2.585864	4.275562

EICO 11
1 2

C	-2.294194	3.237806	-3.505799
C	-1.626432	4.028147	-2.900106
H	-2.882130	2.527964	-4.049287
H	-1.016702	4.727397	-2.371951
C	1.054697	3.197221	-0.268344
C	0.396807	3.982421	0.353791
H	1.647611	2.505523	-0.825404
H	-0.173435	4.702437	0.901306
C	-2.688242	-2.650807	-0.197451
C	-1.873912	-2.032105	0.427429
H	-3.395524	-3.201741	-0.779526
H	-1.143111	-1.483933	0.982737
C	-4.840063	0.915496	-1.884465
C	-3.836919	0.275833	-2.039620
H	-5.742068	1.475047	-1.744425
H	-2.931620	-0.276756	-2.177375
C	-6.724360	-1.589490	-0.379962
C	-7.703187	-1.813821	0.273699
H	-5.859722	-1.387920	-0.975165
H	-8.576663	-2.016518	0.852150
C	-8.198209	2.667040	-1.404163
C	-8.397913	1.505587	-1.624232
H	-8.023110	3.705043	-1.226096
H	-8.574308	0.473003	-1.833640
C	-6.039090	3.184556	-4.484133
C	-5.300287	3.876811	-5.124173
H	-6.710621	2.568780	-3.926877
H	-4.648494	4.493659	-5.701494
C	-3.589356	0.112985	-5.406188
C	-2.408695	-0.080993	-5.340507
H	-4.642196	0.289242	-5.466498
H	-1.355569	-0.246742	-5.292663
C	-2.676407	2.524835	1.675887
C	-2.639599	2.647923	0.352816
H	-2.185125	3.263188	2.305465
H	-2.988242	2.165708	-0.553930
C	-7.786959	0.096283	-4.935460
C	-7.113363	-0.693851	-4.337949
H	-8.391401	0.789406	-5.476807
H	-6.515173	-1.418122	-3.827762
C	-1.252388	2.926910	4.947837
C	-2.278615	3.298624	5.441729
H	-0.334625	2.583202	4.523706
H	-3.182863	3.629668	5.902044
C	-0.297407	0.637347	-2.177037
C	0.061632	-0.464348	-1.870117
H	-0.619386	1.616487	-2.466677
H	0.381267	-1.448038	-1.607444
C	-4.420942	-2.995533	-3.453100
C	-5.219017	-3.651841	-2.846409
H	-3.725189	-2.412259	-4.018097

H	-5.935518	-4.242826	-2.320552
C	-4.176480	-0.901837	2.582499
C	-5.242044	-0.230535	2.539562
H	-3.535729	-1.744491	2.769672
H	-6.291127	-0.007185	2.655627
C	0.032593	0.594376	2.276503
C	-0.157851	-0.132591	3.210289
H	0.215371	1.252900	1.452853
H	-0.293740	-0.779175	4.048805
C	-4.953223	4.494016	-1.247735
C	-5.321527	4.428128	-0.108086
H	-4.637285	4.538912	-2.268758
H	-5.652041	4.374551	0.907443
C	-2.485753	6.058939	1.668141
C	-1.840982	6.087386	2.678028
H	-3.067988	6.038064	0.771565
H	-1.268569	6.127351	3.578645
C	-8.783725	0.764770	3.242385
C	-8.589678	1.098343	2.107484
H	-8.987485	0.471618	4.248485
H	-8.440468	1.393238	1.088893
C	-6.040462	3.405846	3.495193
C	-5.088726	4.134493	3.467522
H	-6.907521	2.779985	3.516276
H	-4.251022	4.798752	3.428569
C	-4.060128	0.410932	1.735511
C	-3.375500	1.472973	2.338726
H	-4.076979	0.328970	0.653317
H	-3.360502	1.502126	3.427958

EICO 12

1 2

C	1.274121	1.804850	2.573489
C	1.887357	0.848480	2.956643
H	0.719043	2.655130	2.235424
H	2.442580	0.007124	3.315163
C	-1.901341	0.154523	-0.102251
C	-1.111926	0.114288	0.884160
H	-2.848071	0.049151	-0.607908
H	-0.771180	-0.055346	1.890229
C	-5.115329	-1.305498	-1.131424
C	-5.478875	-0.399950	-0.435699
H	-4.802398	-2.118274	-1.748971
H	-5.798662	0.408448	0.186141
C	-4.475843	1.834555	2.371927
C	-4.927024	2.744360	1.736668
H	-4.085428	1.023272	2.947498
H	-5.332653	3.563651	1.185839
C	-3.296967	-4.129851	-0.116760
C	-2.479746	-4.396062	-0.952225
H	-4.052077	-3.918718	0.612111
H	-1.771806	-4.677721	-1.699245
C	4.025490	-1.689088	0.343538
C	4.224536	-0.507876	0.285966
H	3.868027	-2.746171	0.412535
H	4.418281	0.544121	0.263690
C	-1.483718	3.838809	1.083220
C	-0.575253	4.320525	0.467682
H	-2.308812	3.425057	1.623402
H	0.226859	4.755721	-0.086483
C	0.422832	-1.567409	-1.108465
C	1.470192	-2.244084	-1.675130

H	-0.384309	-2.110362	-0.629738
H	2.275817	-1.678248	-2.137684
C	-0.476669	0.670914	-0.426135
C	0.453881	-0.173615	-1.083380
H	-0.313770	1.740377	-0.503482
H	1.288193	0.319789	-1.582222
C	3.439264	-5.373766	1.218968
C	4.615395	-5.143009	1.214958
H	2.393870	-5.597486	1.222220
H	5.664509	-4.945467	1.210687
C	-0.103450	-6.704378	0.073241
C	0.747049	-7.011280	-0.713079
H	-0.873764	-6.454717	0.767888
H	1.511220	-7.298297	-1.404108
C	1.589056	-3.678780	-1.661346
C	2.644458	-4.275772	-2.190693
H	0.810869	-4.263126	-1.171999
H	3.575843	-4.016440	-2.680673
C	4.160744	-7.327384	-1.845808
C	3.980902	-7.721924	-2.962615
H	4.317369	-6.975306	-0.848634
H	3.829017	-8.088641	-3.952977
C	-0.109273	-3.291214	1.911259
C	0.997217	-3.039605	2.298407
H	-1.098662	-3.509079	1.570338
H	1.980444	-2.829402	2.664767
C	-2.777519	-1.683714	3.511692
C	-3.572050	-1.617005	2.617309
H	-2.072957	-1.756455	4.309925
H	-4.294494	-1.566432	1.830820
C	4.610969	2.913392	1.496487
C	5.391353	3.173551	0.625333
H	3.916136	2.682269	2.275200
H	6.098283	3.423106	-0.134548
C	-6.666945	-3.526699	1.377833
C	-6.150140	-3.839499	2.411605
H	-7.144416	-3.247470	0.465742
H	-5.704680	-4.115019	3.340891
C	2.342401	3.020154	-1.494729
C	1.611123	3.081113	-2.441938
H	3.009669	2.994140	-0.658179
H	0.970345	3.157449	-3.291883
C	6.235511	-4.467848	-2.212213
C	6.242340	-3.338295	-1.813512
H	6.230497	-5.474768	-2.567677
H	6.258568	-2.333687	-1.454521
C	4.326318	-1.843105	3.838615
C	3.626997	-2.154460	4.759721
H	4.953345	-1.563171	3.021523
H	3.025321	-2.438932	5.593718

EICO 13

1 2

C	6.054438	1.544232	-1.041290
C	5.947956	2.507498	-0.335860
H	6.165576	0.688896	-1.676334
H	5.858834	3.363529	0.296104
C	2.883289	4.658076	1.192244
C	3.322545	3.756337	1.848579
H	2.485324	5.465998	0.617223
H	3.700242	2.952586	2.441515
C	0.280311	5.535341	-1.333926

C	1.318078	5.604810	-1.929265
H	-0.653264	5.478206	-0.818718
H	2.225806	5.692967	-2.486434
C	2.472396	2.226726	-1.472118
C	2.969763	1.859745	-2.568149
H	2.871185	2.690081	-0.574166
H	3.215061	1.473156	-3.543344
C	-2.641135	3.166017	-1.145355
C	-2.242084	3.119964	-0.015369
H	-3.008755	3.188065	-2.150379
H	-1.890747	3.076871	0.994780
C	-0.024829	2.687811	2.825410
C	-0.913264	2.081306	3.354758
H	0.773372	3.238498	2.375260
H	-1.697136	1.542172	3.847179
C	2.101503	-1.481426	-2.743333
C	2.326948	-1.820754	-1.616019
H	1.923687	-1.185104	-3.755057
H	2.531379	-2.147773	-0.617975
C	2.899841	-3.519503	1.697571
C	2.591725	-4.357612	0.899382
H	3.178215	-2.782625	2.417745
H	2.331064	-5.117301	0.196577
C	0.852961	2.056683	-1.386791
C	0.496063	0.978404	-0.860614
H	0.339032	2.927644	-1.781616
H	0.287070	0.028677	-0.396511
C	-1.181756	-1.005897	1.516218
C	-0.381302	-1.875330	1.315422
H	-1.917034	-0.253192	1.701130
H	0.333260	-2.657446	1.167556
C	2.325581	0.216842	1.830362
C	3.357717	0.255928	1.220835
H	1.407387	0.184034	2.377583
H	4.283993	0.313521	0.690765
C	5.728181	-1.401981	-3.317263
C	6.597597	-0.773742	-3.851491
H	4.947545	-1.964662	-2.854041
H	7.380795	-0.224078	-4.325101
C	-2.304730	-0.289185	-1.904182
C	-1.642353	-0.841240	-2.736871
H	-2.913297	0.180592	-1.160208
H	-1.070128	-1.361111	-3.472201
C	3.563456	5.696430	-5.007790
C	2.668628	6.489300	-5.084034
H	4.371107	4.998104	-4.956706
H	1.883820	7.208537	-5.157246
C	-4.047562	1.806688	-4.297152
C	-3.790313	2.888392	-4.742172
H	-4.282332	0.840490	-3.909695
H	-3.569012	3.846002	-5.157694
C	3.421288	-0.043306	-5.780783
C	2.417255	0.564630	-6.026333
H	4.321209	-0.580366	-5.566797
H	1.524755	1.108859	-6.246721
C	-4.799627	0.828533	0.849626
C	-4.698999	-0.359809	0.964131
H	-4.897060	1.885582	0.737146
H	-4.623839	-1.419340	1.071719
C	6.015975	2.951499	-4.231918
C	5.567199	2.630494	-5.295554
H	6.426290	3.234457	-3.287659
H	5.176800	2.347455	-6.247964

C	-3.818913	0.056522	4.532799
C	-2.973534	-0.297349	5.303880
H	-4.581956	0.359439	3.849733
H	-2.235673	-0.617009	6.005905
C	-0.275798	2.528394	-4.469582
C	0.585718	3.329893	-4.701453
H	-1.069521	1.839821	-4.270588
H	1.343989	4.053655	-4.919507

EICO 14
1 2

C	0.313260	1.750717	0.969247
C	0.945102	1.526412	1.962830
H	-0.265934	1.942135	0.093111
H	1.501647	1.330939	2.856503
C	2.775410	0.674568	-1.800242
C	3.622496	-0.172159	-1.780175
H	2.020707	1.430650	-1.816744
H	4.362358	-0.940331	-1.741284
C	1.580465	4.665752	3.343970
C	2.655969	5.044718	3.711941
H	0.621896	4.318791	3.026210
H	3.613116	5.371075	4.056019
C	3.744491	3.251024	0.668824
C	4.972482	2.952793	0.560197
H	2.690121	3.059833	0.792380
H	5.820479	2.289655	0.495481
C	2.910761	1.646769	5.161542
C	3.479550	0.719239	4.659773
H	2.403795	2.467576	5.618148
H	3.986138	-0.109123	4.214928
C	4.315711	2.467301	-4.471347
C	4.394390	3.666667	-3.916027
H	4.290275	1.433550	-4.149177
H	4.398378	4.564186	-4.531549
C	4.637909	5.041125	-1.896216
C	4.494068	3.808032	-2.487776
H	4.676933	5.906583	-2.549546
H	4.464567	2.899274	-1.896429
C	2.026631	6.942401	0.466524
C	2.113226	7.267045	-0.684620
H	1.944401	6.658796	1.494459
H	2.187790	7.556536	-1.712040
C	4.699198	4.463872	0.626874
C	4.773098	5.302111	-0.529063
H	4.908389	4.968482	1.565377
H	4.962170	6.350272	-0.304414
C	6.661077	4.425987	3.695115
C	5.889171	3.544404	3.948562
H	7.354257	5.207923	3.474645
H	5.193707	2.766352	4.189246
C	7.635596	2.509363	-5.508045
C	7.436836	1.554718	-6.203390
H	7.817932	3.369704	-4.901159
H	7.276792	0.703630	-6.826340
C	7.924851	6.490732	0.226598
C	8.144192	5.322299	0.379402
H	7.738006	7.533103	0.086660
H	8.354581	4.283279	0.523787
C	0.492517	3.359827	-2.781594
C	0.860949	4.102203	-1.915570
H	0.151304	2.705347	-3.553211
H	1.171023	4.795714	-1.161342

C	5.109347	9.039908	-1.311269
C	5.847101	8.507324	-2.091543
H	4.452039	9.523964	-0.623270
H	6.503508	8.033459	-2.791183
C	3.032477	7.432648	-4.469146
C	2.201342	6.595213	-4.256840
H	3.771941	8.175816	-4.683808
H	1.458961	5.851568	-4.063577
C	5.568491	-0.619060	1.703087
C	4.423093	-0.384343	1.442350
H	6.588931	-0.833021	1.930229
H	3.403057	-0.186690	1.195378
C	5.942178	9.606431	-5.504482
C	5.856681	8.844153	-6.424185
H	6.024575	10.299871	-4.697418
H	5.789842	8.179316	-7.256942
C	7.256117	5.990663	-4.446652
C	7.804864	5.766585	-3.404735
H	6.768910	6.188363	-5.377408
H	8.290411	5.591334	-2.468955
C	8.248503	1.845035	1.592179
C	8.628380	1.455135	0.524679
H	7.908921	2.199604	2.542247
H	8.988182	1.094068	-0.412975
C	4.423117	5.518933	-7.209231
C	5.332732	4.744662	-7.302966
H	3.601247	6.195204	-7.128112
H	6.132582	4.040657	-7.383930

EICO 15

1 2

C	0.370610	-1.822845	-0.098315
C	1.209463	-1.373274	0.629092
H	-0.385055	-2.247443	-0.721042
H	1.965653	-0.985370	1.276993
C	2.010948	2.776014	-3.702702
C	2.598712	1.815378	-2.882811
H	1.283153	2.465641	-4.445474
H	3.325353	2.144181	-2.141574
C	0.288494	1.535951	4.111778
C	0.279604	2.710989	3.879355
H	0.271074	0.491077	4.326486
H	0.276548	3.761035	3.680902
C	0.923451	2.079738	-0.043083
C	1.471505	3.114675	0.212999
H	0.438716	1.145297	-0.225216
H	1.943229	4.034734	0.483697
C	3.690091	3.145934	2.850234
C	4.622382	3.767244	2.422376
H	2.848467	2.605403	3.229730
H	5.452924	4.331007	2.051539
C	6.802383	6.498378	-3.394930
C	6.747447	5.300444	-3.399890
H	6.876422	7.563728	-3.380095
H	6.734065	4.230262	-3.386763
C	7.357437	5.790145	0.354241
C	7.596433	5.859888	1.526096
H	7.158036	5.739842	-0.693955
H	7.803082	5.935022	2.570577
C	0.680443	6.253118	2.311527
C	1.691405	6.080031	2.932272
H	-0.216642	6.414243	1.756436

H	2.591074	5.926484	3.490240
C	1.923764	4.236162	-3.115630
C	2.829636	4.090942	-3.976580
H	1.255306	4.727653	-2.423911
H	3.600008	4.362058	-4.682332
C	3.753046	6.629807	-1.380220
C	3.160011	6.636261	-0.338209
H	4.307695	6.622764	-2.293095
H	2.626567	6.658023	0.591066
C	5.335884	7.291972	3.870421
C	5.202391	6.325791	4.565450
H	5.466623	8.155461	3.257909
H	5.061985	5.466860	5.183267
C	2.859024	-0.390979	-2.060575
C	2.307860	0.418550	-2.957995
H	3.516117	-0.313291	-1.197417
H	1.633035	0.049672	-3.727621
C	5.109655	3.373618	-0.857572
C	5.790039	2.394727	-0.983529
H	4.526151	4.262186	-0.733191
H	6.429393	1.544084	-1.067644
C	2.509611	1.690147	-7.042406
C	1.448665	2.128745	-7.386928
H	3.456775	1.290468	-6.749980
H	0.503595	2.501765	-7.717563
C	4.191700	4.918279	-7.245457
C	5.085202	5.448826	-6.648205
H	3.403699	4.445720	-7.790052
H	5.884776	5.922893	-6.121670
C	5.703008	2.182058	-5.253308
C	5.802715	1.303893	-4.444209
H	5.622140	2.964471	-5.980195
H	5.925892	0.516845	-3.733719
C	2.255990	4.340743	6.323272
C	2.460938	5.510311	6.477293
H	2.051903	3.301238	6.196966
H	2.643530	6.551672	6.620703
C	-0.815785	5.465553	-1.022991
C	-0.248974	6.485668	-1.291056
H	-1.326236	4.562942	-0.773578
H	0.247372	7.400746	-1.524284
C	9.080960	2.273519	-2.883885
C	9.012247	2.880947	-1.854234
H	9.163725	1.741722	-3.804578
H	8.960609	3.425309	-0.937161
C	4.823738	-1.060051	0.945438
C	4.681884	0.104721	1.191663
H	4.960695	-2.101755	0.756788
H	4.577970	1.143871	1.430935

EICO 16

1 2

C	0.645105	2.476644	-1.174737
C	1.360523	2.207590	0.021497
H	-0.311249	2.089668	-1.523909
H	1.356633	1.462988	0.801253
C	1.117189	0.669680	-3.804541
C	1.197376	-0.093144	-2.883926
H	1.054936	1.325008	-4.644552
H	1.277523	-0.790995	-2.076441
C	0.448477	4.017223	3.303197
C	1.497889	4.594216	3.363676

H	-0.497586	3.519776	3.280763
H	2.424008	5.123163	3.423968
C	4.058788	0.834969	-0.918632
C	4.638731	1.790298	-1.350262
H	3.556013	-0.028220	-0.538106
H	5.165362	2.636811	-1.730313
C	-2.899655	1.433336	-1.848169
C	-2.272861	0.611491	-2.455551
H	-3.481038	2.146012	-1.304983
H	-1.726680	-0.114956	-3.015465
C	-3.307472	2.692015	4.018633
C	-2.853463	3.700801	4.482268
H	-3.729113	1.804270	3.598986
H	-2.442260	4.599130	4.893200
C	1.306824	7.328423	-0.359984
C	0.629573	7.022481	0.581100
H	1.899537	7.639021	-1.191317
H	0.005909	6.773063	1.417298
C	-1.894996	4.804746	-0.261396
C	-2.131972	4.017339	0.610880
H	-1.700132	5.532959	-1.017113
H	-2.371552	3.333183	1.397684
C	-0.827560	0.512985	2.417935
C	-1.372978	0.475310	1.350013
H	-0.357363	0.538597	3.380586
H	-1.871266	0.435356	0.403716
C	2.079467	7.588435	5.059779
C	2.198838	8.188350	4.030174
H	1.957550	7.058946	5.979141
H	2.303269	8.739497	3.122710
C	-5.593586	5.414787	2.430667
C	-5.607919	5.771027	1.287220
H	-5.579943	5.098748	3.450388
H	-5.640290	6.097941	0.272112
C	4.101610	5.163080	0.901134
C	4.286051	4.008707	1.167047
H	3.945676	6.196348	0.684836
H	4.457062	2.981084	1.408087
C	2.842501	1.244110	3.067872
C	3.460615	0.334542	2.589453
H	2.298076	2.059029	3.496272
H	4.026946	-0.473376	2.173907
C	2.326621	-2.361115	-0.181978
C	1.475347	-1.805456	0.452891
H	3.091142	-2.866185	-0.730477
H	0.710386	-1.332283	1.031245
C	1.478708	3.620490	-0.052141
C	1.297262	3.710084	-1.460840
H	1.346604	4.367184	0.725018
H	1.169523	4.508999	-2.172737
C	6.001871	-1.648043	0.608591
C	5.549320	-2.528300	1.282569
H	6.415211	-0.868700	0.008009
H	5.163771	-3.321041	1.883721
C	-2.307178	6.839176	2.975022
C	-1.326869	7.080939	3.621083
H	-3.189940	6.618519	2.414333
H	-0.444592	7.297245	4.184499
C	-0.282417	1.385339	5.881555
C	0.813825	0.901714	5.888161
H	-1.262149	1.812540	5.880627
H	1.786180	0.461752	5.908280
C	-0.759840	5.933178	7.025788

C	-0.106911	4.964725	6.760612
H	-1.340412	6.793474	7.273659
H	0.471137	4.096507	6.532158
C	-5.193057	2.020772	1.129049
C	-4.974318	0.854191	0.965719
H	-5.403090	3.058875	1.281645
H	-4.805927	-0.188425	0.814754

EICO 17

1 2

C	3.299736	-3.567357	-3.897705
C	3.148922	-2.878274	-4.866141
C	2.219708	-0.549817	-1.995488
C	1.770038	0.326336	-2.679858
C	0.539009	1.851941	-0.046855
C	-0.747291	1.328715	-0.167072
C	-1.045648	0.049629	0.703414
C	-0.877911	-0.197678	-0.516871
C	0.852212	3.234954	-0.239215
C	2.110384	3.621312	-0.072747
C	0.627473	1.631346	3.303757
C	1.289625	0.645402	3.139041
C	-3.574072	0.280290	-2.578639
C	-3.102538	-0.451016	-3.402673
C	-1.187726	-3.313540	-2.357051
C	-0.184826	-3.017620	-2.945169
C	4.312247	1.098306	1.364550
C	4.463083	0.899824	0.191694
C	-2.431005	-6.136824	-0.007627
C	-1.282685	-6.187361	0.327803
C	0.426008	-3.156634	0.953013
C	0.859878	-3.090178	2.068392
C	-0.884587	2.585892	-3.684595
C	-0.062955	3.453500	-3.773737
C	5.652008	-0.586592	-3.139582
C	6.540683	0.086311	-2.700623
C	4.860629	-2.732976	-0.488695
C	3.893060	-3.310740	-0.079821
C	2.590841	-6.349331	-1.135097
C	1.583756	-5.961968	-1.655210
C	4.050810	-1.868849	3.242776
C	3.756060	-1.675342	4.387971
C	-4.291499	1.067535	1.008254
C	-4.279689	2.189149	0.585236
C	-3.903341	-2.355057	-0.073481
C	-3.261608	-2.912736	0.770971
C	-2.266100	3.852360	3.164372
C	-1.520652	4.726775	3.503436
C	-2.372080	5.161947	-0.187403
C	-2.437028	4.952193	-1.365521
H	3.427206	-4.194405	-3.041177
H	3.027251	-2.279895	-5.741075
H	0.717473	-2.782218	-3.471079
H	-2.071213	-3.582443	-1.818107
H	0.047453	-3.235339	-0.044137
H	1.265254	-3.043326	3.055353
H	-0.837292	-0.768064	-1.429882
H	-1.233279	-0.109558	1.749502
H	5.719959	-2.221986	-0.865808
H	3.032149	-3.827309	0.285660
H	4.198372	1.260418	2.413684
H	4.624389	0.714927	-0.850122
H	1.363241	1.103350	-3.291509

H	2.648502	-1.330910	-1.400839
H	-4.324985	0.063166	1.372214
H	-4.273437	3.189596	0.208461
H	-2.693011	-1.112252	-4.134363
H	-4.007888	0.914149	-1.834963
H	0.058403	3.927651	-0.512474
H	3.056203	3.155288	0.188490
H	4.872822	-1.201633	-3.537396
H	7.342395	0.682969	-2.326946
H	-2.944865	3.081716	2.868157
H	-0.871325	5.511043	3.823940
H	-0.258238	-6.248385	0.620299
H	-3.457381	-6.108180	-0.296203
H	-1.568697	1.995359	-0.415360
H	1.350592	1.177830	0.216122
H	-1.624393	1.815452	-3.618979
H	0.660248	4.232313	-3.872563
H	-2.320292	5.348258	0.863737
H	-2.486295	4.783211	-2.418400
H	4.323265	-2.047099	2.223861
H	3.513981	-1.508020	5.413634
H	-4.472178	-1.868407	-0.834665
H	-2.690670	-3.436812	1.506129
H	0.681472	-5.629976	-2.120410
H	3.488070	-6.707811	-0.682328
H	0.027313	2.502241	3.463809
H	1.896972	-0.227352	3.019810

EICO 18
1 2

C	-0.708345	0.234353	-2.769899
C	-0.759562	1.338771	-3.497611
H	-0.633149	-0.743675	-3.241610
H	-0.845723	2.413053	-3.377358
C	2.691514	1.042517	-2.123272
C	2.715565	1.407496	-0.982839
H	2.683058	0.720609	-3.140335
H	2.752227	1.718217	0.037942
C	-4.212591	0.457688	1.074575
C	-4.709866	-0.633348	1.069044
H	-3.818910	1.452401	1.094939
H	-5.160175	-1.602260	1.069341
C	-0.014620	3.997501	-0.733022
C	-1.160380	4.119127	-0.398510
H	1.005142	3.931297	-1.050195
H	-2.179287	4.248766	-0.094527
C	-3.259447	-2.829093	-2.856761
C	-3.583887	-1.983079	-2.072567
H	-2.987149	-3.580936	-3.563797
H	-3.886341	-1.236563	-1.368395
C	1.852709	0.611155	2.743355
C	2.372945	-0.210717	2.044020
H	1.389735	1.338823	3.373026
H	2.856961	-0.931740	1.423426
C	-0.791913	-0.831301	-0.543820
C	-0.788207	0.293114	-1.337465
H	-0.723555	-1.814812	-0.998555
H	-0.867400	1.277438	-0.877936
C	-4.151718	1.809853	-2.544935
C	-3.658761	2.902381	-2.517033
H	-4.632184	0.856153	-2.587332
H	-3.249968	3.890760	-2.500718
C	-1.067014	2.668228	2.741261

C	-1.622357	2.031851	3.591222
H	-0.584690	3.244836	1.980327
H	-2.121681	1.483105	4.358117
C	-0.694601	-1.674466	3.189321
C	-1.942541	-1.665259	2.964355
H	0.198450	-1.660830	3.788913
H	-2.992689	-1.625160	3.191694
C	-6.891644	-0.966517	-2.290813
C	-7.110613	0.123742	-1.846067
H	-6.712547	-1.938477	-2.691223
H	-7.313870	1.102256	-1.468137
C	2.736289	5.750400	-2.248402
C	3.333661	4.723786	-2.405681
H	2.203638	6.668795	-2.120759
H	3.878897	3.816193	-2.535828
C	-5.072266	4.297686	-5.578608
C	-5.019369	5.380248	-5.069005
H	-5.128872	3.338469	-6.041514
H	-4.969764	6.342255	-4.608909
C	-3.900561	-4.431184	2.016957
C	-3.728380	-4.137091	0.868774
H	-4.062780	-4.720321	3.031106
H	-3.589498	-3.888803	-0.161407
C	-1.067205	-1.838829	1.705191
C	-0.953221	-0.719703	0.830059
H	-1.005041	-2.827305	1.255103
H	-1.025226	0.272270	1.275766
C	-1.353857	4.827641	-4.694945
C	-0.239843	4.874402	-4.254837
H	-2.349213	4.795118	-5.086443
H	0.757158	4.923933	-3.871226
C	-4.155788	4.206280	1.787357
C	-4.739066	3.907348	0.784139
H	-3.644421	4.475639	2.684091
H	-5.274970	3.648294	-0.104701
C	-3.137559	6.615351	-2.035305
C	-4.257038	6.387380	-1.672686
H	-2.139164	6.832293	-2.351500
H	-5.256690	6.192144	-1.351402
C	-7.742267	4.089979	-1.213993
C	-7.081326	3.991453	-2.208418
H	-8.344202	4.187423	-0.338315
H	-6.495110	3.918358	-3.099740
C	0.070456	8.408181	-1.893721
C	0.143324	8.216305	-3.073463
H	-0.002567	8.595646	-0.846036
H	0.202504	8.053621	-4.126494

EICO 19
1 2

C	0.907530	2.035990	2.755430
C	0.070405	1.191735	2.600809
C	-3.002042	2.687627	2.299169
C	-3.868638	2.426698	1.513551
C	-6.115687	-0.236793	1.500286
C	-5.975275	-0.798847	0.451968
C	1.855924	-1.854202	2.816392
C	1.960267	-2.868998	2.186775
C	-1.582715	2.896751	-1.134541
C	-1.272985	3.302359	-2.219272
C	1.521259	1.297917	-1.171907
C	2.437528	1.778044	-0.460113
C	2.191117	0.253414	-0.174868

C	2.983671	-0.722244	-0.769899
C	3.767397	-0.564634	-1.960427
C	4.396438	-1.626376	-2.442489
C	0.511755	-2.217405	-2.409962
C	0.036777	-2.277595	-1.310637
C	-0.468513	0.495023	-4.136767
C	0.491058	0.893248	-4.734567
C	5.910846	1.492395	-0.062770
C	5.946454	2.434431	-0.801988
C	-3.224154	-1.133367	-2.029841
C	-3.577650	0.006635	-2.147076
C	-2.383708	-1.198368	1.387335
C	-1.783396	-1.985206	2.063966
C	5.459115	-2.040750	1.494953
C	6.391651	-1.971057	0.745778
C	-2.320403	-4.767227	-1.540806
C	-2.879497	-4.548427	-0.504041
C	3.920790	-4.695107	-0.383366
C	2.857579	-4.636947	-0.933553
C	-4.911368	-3.814158	2.522291
C	-4.672634	-4.967251	2.739614
C	-5.252075	3.331026	-1.698073
C	-6.113234	2.510121	-1.559830
C	3.024808	4.755806	1.456430
C	4.122140	4.304864	1.627643
C	-0.644575	5.347680	1.423143
C	-0.338444	5.766164	2.502606
H	1.644283	2.797188	2.894668
H	-0.683586	0.442913	2.467662
H	-2.231653	2.936671	2.994567
H	-4.645524	2.217123	0.809912
H	1.645958	-0.007938	0.724371
H	3.001184	-1.705989	-0.303034
H	-0.929509	4.984035	0.460316
H	-0.077121	6.150460	3.463075
H	5.107806	3.923156	1.774020
H	2.045712	5.169311	1.331071
H	-1.005167	3.651182	-3.191868
H	-1.883520	2.556888	-0.164196
H	0.904623	-2.148862	-3.400006
H	-0.420328	-2.326768	-0.343423
H	-1.264217	-2.688049	2.677069
H	-2.944322	-0.509818	0.792068
H	5.998813	3.280619	-1.450455
H	5.900337	0.648170	0.592931
H	4.866145	-4.759170	0.109077
H	1.905086	-4.589258	-1.414893
H	-1.831681	-4.973429	-2.465656
H	-3.388543	-4.372234	0.421102
H	2.063558	-3.778832	1.635517
H	1.745357	-0.954144	3.382084
H	-4.489217	4.067419	-1.818271
H	-6.887315	1.786184	-1.433412
H	-3.917200	1.015627	-2.249278
H	-2.930896	-2.155454	-1.909433
H	-6.247067	0.263553	2.433623
H	-5.845136	-1.298531	-0.482078
H	3.078879	2.521823	-0.011817
H	0.717526	1.207661	-1.885014
H	-5.136170	-2.789086	2.323558
H	-4.475798	-5.996074	2.939589
H	7.237928	-1.907982	0.098007
H	4.632745	-2.117706	2.169154

H	3.835439	0.406821	-2.441459
H	4.496691	-2.676400	-2.187009
H	1.326364	1.248564	-5.295141
H	-1.330095	0.139243	-3.608529

EICO 20

1 2

C	1.214620	0.117598	1.985352
C	2.365369	0.410388	1.821200
H	0.190735	-0.150620	2.125994
H	3.393377	0.668576	1.676506
C	2.754378	5.477007	-4.724234
C	3.026354	4.308922	-4.705217
H	2.510664	6.518431	-4.771567
H	3.262663	3.266004	-4.708154
C	2.372581	6.969178	4.413930
C	1.501803	6.224464	4.765270
H	3.143495	7.650127	4.129981
H	0.724772	5.566247	5.095715
C	5.356385	2.568079	0.982667
C	5.267851	2.615781	2.177639
H	5.447521	2.521366	-0.083402
H	5.194148	2.643868	3.242989
C	-1.992303	2.100403	1.545989
C	-1.760228	2.112942	2.721805
H	-2.222944	2.088372	0.503774
H	-1.568929	2.137017	3.774085
C	4.811187	1.864899	-2.600123
C	5.898674	2.356249	-2.704695
H	3.839175	1.426535	-2.538094
H	6.869532	2.788414	-2.802375
C	1.318794	3.885260	2.309596
C	0.554003	4.736897	1.467135
H	1.194533	2.925430	2.784166
H	-0.339071	4.531259	0.881302
C	1.484109	3.483745	-1.619993
C	1.375198	2.451057	-1.019392
H	1.582136	4.398220	-2.168098
H	1.293838	1.528928	-0.483204
C	0.679472	7.224845	-1.918145
C	1.864817	7.396540	-1.860729
H	-0.379194	7.093802	-2.002458
H	2.920350	7.553531	-1.817824
C	1.584941	5.688562	1.226585
C	2.328392	4.519928	1.537632
H	1.704460	6.544275	0.579700
H	3.212532	4.101147	1.060373
C	-1.663151	3.181537	-4.666257
C	-0.613753	3.749377	-4.779428
H	-2.601284	2.678439	-4.587853
H	0.328273	4.242887	-4.902406
C	1.634930	0.886074	-4.542711
C	2.644007	0.310006	-4.833372
H	0.727279	1.396928	-4.306933
H	3.536652	-0.210138	-5.100334
C	-2.444607	5.333619	-0.593411
C	-1.850115	4.576041	-1.307152
H	-2.981951	6.017082	0.026159
H	-1.333049	3.919241	-1.973644
C	3.173355	3.069850	5.260362
C	3.056225	1.902320	5.016997
H	3.274928	4.109357	5.486003

H	2.959050	0.860207	4.805330
C	-2.063808	5.650379	2.844973
C	-1.460648	6.669102	3.032188
H	-2.601602	4.741891	2.679089
H	-0.932839	7.578541	3.218279
C	-1.306959	3.957624	5.894525
C	-0.339644	3.269359	6.056102
H	-2.169931	4.573404	5.770902
H	0.528037	2.667647	6.217305
C	5.139849	5.849926	-0.368390
C	4.651217	5.410772	-1.370692
H	5.608641	6.231158	0.510937
H	4.242452	5.022603	-2.280229
C	-0.129828	10.745012	-2.475349
C	-0.979502	11.257012	-1.804810
H	0.624189	10.293560	-3.082834
H	-1.728332	11.730528	-1.211304
C	-2.260396	8.184039	-3.780344
C	-2.292383	7.041424	-4.139911
H	-2.218451	9.202681	-3.459596
H	-2.326764	6.024393	-4.463676
C	2.127124	9.182538	-5.512389
C	1.044639	8.702011	-5.332628
H	3.083776	9.619018	-5.692542
H	0.072243	8.285789	-5.174343
